# Polymers in Physics, Chemistry and Biology: Behavior of Linear Polymers in Fractal Structures

**DOI:** 10.3390/polym16233400

**Published:** 2024-12-02

**Authors:** Hector Eduardo Roman

**Affiliations:** Department of Physics, University of Milano-Bicocca, Piazza della Scienza 3, 20126 Milano, Italy; hector.roman@unimib.it

**Keywords:** linear polymers, self-avoiding random walks, fractals, reptation, proteins and evolution, deposition of thin polymeric films, packing of DNA chains within the cell

## Abstract

We start presenting an overview on recent applications of linear polymers and networks in condensed matter physics, chemistry and biology by briefly discussing selected papers (published within 2022–2024) in some detail. They are organized into three main subsections: polymers in physics (further subdivided into simulations of coarse-grained models and structural properties of materials), chemistry (quantum mechanical calculations, environmental issues and rheological properties of viscoelastic composites) and biology (macromolecules, proteins and biomedical applications). The core of the work is devoted to a review of theoretical aspects of linear polymers, with emphasis on self-avoiding walk (SAW) chains, in regular lattices and in both deterministic and random fractal structures. Values of critical exponents describing the structure of SAWs in different environments are updated whenever available. The case of random fractal structures is modeled by percolation clusters at criticality, and the issue of multifractality, which is typical of these complex systems, is illustrated. Applications of these models are suggested, and references to known results in the literature are provided. A detailed discussion of the reptation method and its many interesting applications are provided. The problem of protein folding and protein evolution are also considered, and the key issues and open questions are highlighted. We include an experimental section on polymers which introduces the most relevant aspects of linear polymers relevant to this work. The last two sections are dedicated to applications, one in materials science, such as fractal features of plasma-treated polymeric materials surfaces and the growth of polymer thin films, and a second one in biology, by considering among others long linear polymers, such as DNA, confined within a finite domain.

## 1. Introduction

The study of polymers is tantamount to new scientific and technological developments during the last decades. Among them, linear polymers play an important role in this review, which focuses on the theoretical and simulation approaches of such structures embedded in complex environments, either in the form of single chains or a many polymer system such as a melt. The core of this work concerns statistical properties of single chains, modeled by self-avoiding walks (SAWs), confined within scale-invariant structures such as deterministic and random fractals. Accurate methods are discussed to generate long SAW chains in arbitrary geometries, of which the reptation algorithm plays a major role. The interest in the latter is justified by the many potential applications it can deliver to deal with both static as well as dynamic chain configurations. This review on SAWs complements the existing literature on the subject and should be useful to polymer scientists involved in complex modeling for the interpretation of experimental results.

Linear polymers are constituted by units which may display a broad type of structures, ranging from quite simple repeated ones (homopolymers) to different complex molecular units (heteropolymers) as in the case of proteins. For many applications, simplifications are introduced consisting of what is called coarse-grained modeling. These simplified models are still quite difficult to study theoretically and numerically due the long-range connectivity of the single monomers and their repulsive interactions at short distances.

To alert the reader to recent contributions from the vast polymer community on modeling and simulations aspects, we have selected papers published during 2022–2024 with the aim of illustrating as clearly as possible the impact and relevance of polymers in physics, chemistry and biology. The papers collection is confined to Section 2 aimed at illustrating the ‘state-of-the-art’ in these research areas, and each paper is discussed in sufficient detail so that the non-expert reader can obtain basic knowledge on the subject at first sight without the need to rely on external sources. Also, the subjects are clearly catalogued so that the reader can rapidly move on to the point of particular interest. We expect this format to be useful to obtain a broad idea of the issues of interest in polymer science today.

In addition to the state of the art (Section 2) and the theory of SAWs in fractal structures (Section 3), the overview includes for completeness three more sections: experimental aspects of polymers (Section 4), applications in materials science (Section 5) focusing on the use of plasma techniques, and applications in biology (Section 6) reviewing very recent results (from 2024) on the genome and aminocyanines. The experimental section is included to provide a basic description on linear polymers, such as, e.g., polyethylene, polypropylene, and plasma-treated polymeric surfaces, which is necessary to understand the experimental results to be discussed in Section 5 and Section 6.

Applications in Section 5 cover recent results on the wetting properties of plasma-treated polymeric materials surfaces, which is a subject of current interest in the plasma community. This section brings in the concept of fractal scaling, which is essential to understand the hydrophilicity/hydrophobicity properties of these materials, making contact with many of the issues discussed in Section 3. A further intriguing application is the modeling of thin films formation by attaching linear polymers on plasma-treated surfaces, which is essentially based on an implementation of the reptation algorithm considered in Section 3. Last but not least are the applications in biology dealing with long polymer chains, such as the double-helix of the genome. Issues related to modeling the transcription of DNA and how to deal with many linear chains in confined geometries, such as the nucleus of a cell, are also discussed. The overview ends with the concluding remarks in Section 7.

## 2. State of the Art: Polymers in Physics, Chemistry and Biology

In the following, we discuss results published during 2022–2024 on polymers with the aim of covering, as thoroughly as possible, the theoretical aspects of current interest on these complex systems in the fields of physics, chemistry and biology. The selected papers are organized for convenience into eight main subsections with a brief description of the content in each case.

- Polymers in Physics:
  – (Section 2.1) Coarse-grained models and MD simulations: single polymer, melt, porous and fibrous materials, bottlebrushes, copolymers, surfactant, crystals, anomalous dynamics, translocation, polymerization, rubbers, polyelectrolytes, polymer through capillaries, charged polymers, bioplastics, branched polymers, synthetic hydrogels.
  – (Section 2.2) Structural properties, self-assembly, smart materials: block polymers, amphiphilic diblock copolymers, chains on graphene, knotted chains, additive manufacturing, conjugated polymers, plasma techniques.
- Polymers in Chemistry:
  – (Section 2.3) Structural properties: polymer electrolyte and fuel cells, rubber, thermoplastic polymers, star-branched molecules, dendrimers.
  – (Section 2.4) Quantum mechanical calculations and MD simulations: solubility of polymers, pyrolysis for polystyrene, insulating materials, expoxy resins, oilfield scales inhibitors.
  – (Section 2.5) Environmental issues, batteries, multifunctional materials: aging, gas desorption, biodegradable polymers, gas separation membranes, polymers electrolytes for Li batteries, polymer actuators, 3D printing.
  – (Section 2.6) Rheological properties, viscoelasticity: fluid dynamics, epoxy-matrix composites, entangled polymers, thermoplastics, viscoelasticity, orientational relaxation, polymers rheology.
- Polymers in Biology:
  – (Section 2.7) Macromolecules, proteins, structures and dynamics: large-scale molecular simulations, actin filaments, electric field effects on GLP-2 peptide, soft vesicle, 2D tethered semiflexible polymers, entangled polymer networks, chirality of polymer knots, model polymers vs. globular proteins, polyelectrolyte pore translocation, protein adsorption dynamics, nucleosome dynamics.
  – (Section 2.8) Biomedical applications: 3D polymer hydrophilic networks, skin repair issues, material models for limb orthoses.

### 2.1. Polymers in Physics: Coarse-Grained Models and MD Simulations

Single polymer: A simple linear polymer model is a self-avoiding walk (SAW) defined on a lattice (see Section 3 for more details). The lattice helps us to count the non-local contacts (between non-consecutive nearest neighbors along the chain) for a single chain so that one can study the effects of interactions between monomers if they become close in space [1,2,3,4]. If the interaction is attractive, the chain will eventually collapse below some critical temperature, which is called $T_{\theta }$. In [5], the authors compute all possible SAWs up to 27 steps (N=28 monomers), requiring about $4\times 10^{17}$ different configurations on the simple cubic lattice. They find $T_{\theta }≃3.713\left(2\right)$, in units of the contact energy, which is comparable to accurate very long-chains calculations. They suggest a systematic approach to improve further on these results.

Polymer melt: If we move from a single-chain problem to a many-chain scenario, like in a polymer melt, the situation becomes even more intricate. These systems possess complex viscoelactic behavior due to interchain interactions. Rouse (1953) proposed a tractable model to describe such melts. Despite its popularity and widespread use [6,7,8,9,10], it has many drawbacks. In [11], the author reviews several aspects of the Rouse model, suggesting its inadequacy to describe realistically such complex polymer solutions. Other competing models, such as the Kirkwood–Riseman model, for concentrated solutions are reviewed together with a selection of computer simulation results to test the theoretical predictions. In particular, the so-called reptation model is discussed in association with the highly entangled chains regime. The Kirkwood–Riseman model is extended to include interchain hydrodynamic interactions, yielding pseudoviral series for the dependence on the concentration and molecular weight of the self-diffusion coefficient and shear viscosity [12].

**Figure 1:**
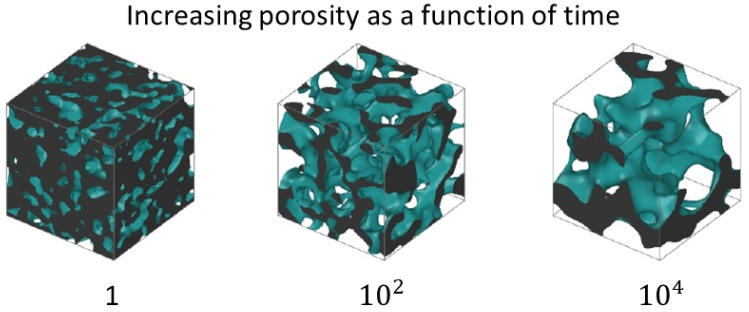
Molecular dynamics simulations of the quenching at temperature *T* of a melt of $N=10^{3}$ linear polymer chains with M=20 monomers each. Along the chain, nearest-neighbor monomers interact via a harmonic potential, and the remaining intra- and inter-chain monomers interact via a Lennard–Jones potential. Here, $T≃\left(1/4\right)\;T_{g}$ of the glass transition temperature $T_{g}$, and time increases from left to right (1, $10^{2}$, $10^{4}$) a.u. (adapted from [13]).

Porous materials: Viscoelastic phase separation and the mechanical properties of a coarse-grained polymer melt model have been studied using MD simulations [13], applying an arrested phase separation strategy by quenching the melt at different times (Figure 1). The resulting systems consist of interpenetrating bicontinuous gas–solid phases, yielding a type of glassy structure characterized by a power-law relaxation decay. In the elastic regime, both the Young and shear moduli of the emerging porous structure attain low values compared to dense glassy systems. For large deformations, the stress–strain curves display a highly intermittent behavior characterized by repeated avalanches of plastic events. The work examines for the first time the role that polymer chain connectivity plays in determining the elastic and plastic responses of these glassy materials (see also [14,15,16,17]).

**Figure 2:**
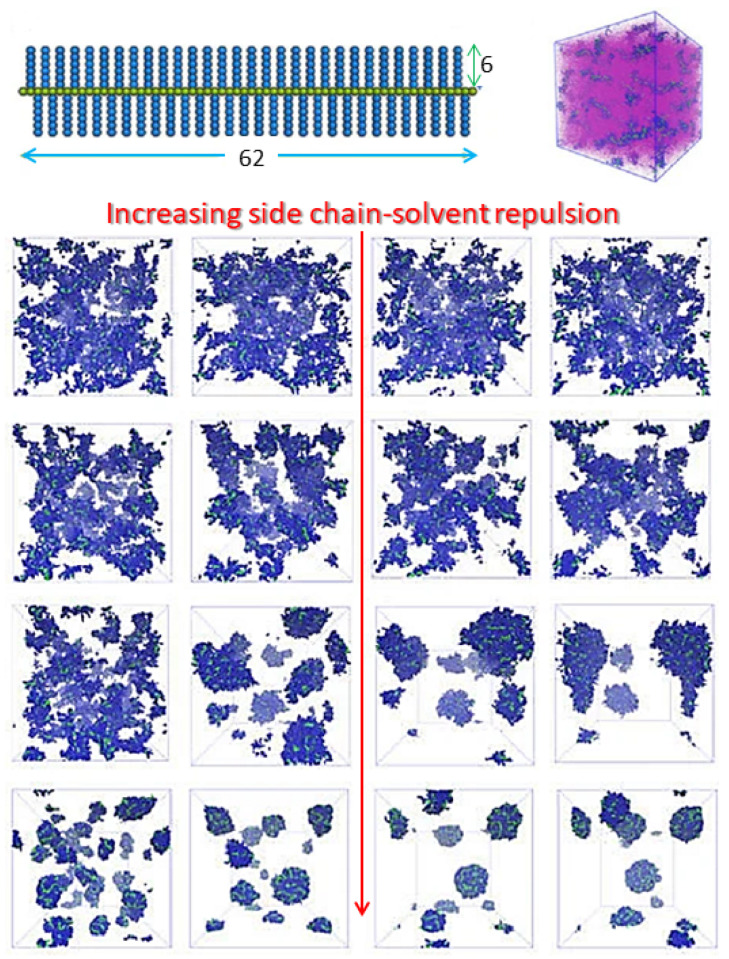
Mesoscale modeling of agglomeration of molecular bottlebrushes. (**Upper panel**) Bottlebrush architecture displaying the backbone beads (green circles) and the side chain ones (blue circles). In this example, the former are 62 and the side chain length is 6. On the right side, a snapshot of the equilibrated system of 77 bottlebrushes in the case of the lowest repulsion parameter between side chain beads and solvent molecules, the latter considered to be a good solvent. (**Lower panel**) Bottlebrushes structures at increasing repulsion parameter (poorer solvents) at four simulations time steps (from left to right): T = ($10^{5}$, $10^{6}$, $2\times 10^{6}$, $3\times 10^{6}$) (adapted from [22]).

Bottlebrushes: Moving onto different polymer shapes, we consider next the case of molecular bottlebrushes (MBBs), which possess a well-defined linear backbone upon which side chains are densely grafted, constituting a type of branched macromolecules [18,19,20,21]. In [22], the authors aim to study the aggregation of MBBs in solvents of various qualities using dissipative particle dynamics (see Figure 2). They study the number of MBB clusters and their size distribution, allowing them to define a mean aggregation number. A low volume fraction of bottlebrushes is considered for different types of solvents and comparing different cutoffs criteria to define which bottlebrushes belong to a cluster. It is found that both short-lived transients and long-lived agglomerates can be present in the system. This work can be extended to study agglomerates of more complex macromolecules as well.

Amphiphilic copolymers: Going a bit up on polymer structure complexity, one finds the large family of amphiphilic copolymers, which comprise both hydrophilic and hydrophobic parts. The copolymers can be built into blocks, i.e., strings of one type of monomers, chemically linked in a linear or branched fashion, which in addition can carry a net charge as in the case of block polyelectrolytes [23,24,25,26,27,28]. In [29], the coarse-grained dissipative particle dynamics (DPD) simulation method is reviewed and its applications to the field of self-organization and self-assembly of amphiphilic copolymers are discussed. Of particular interest are polyelectrolytes with special attention to implementing explicit electrostatics in DPD numerical calculations. The importance and suitability of this coarse-grained method for understanding these complex polymer systems are stressed, and several relevant works are critically discussed.

**Figure 3:**
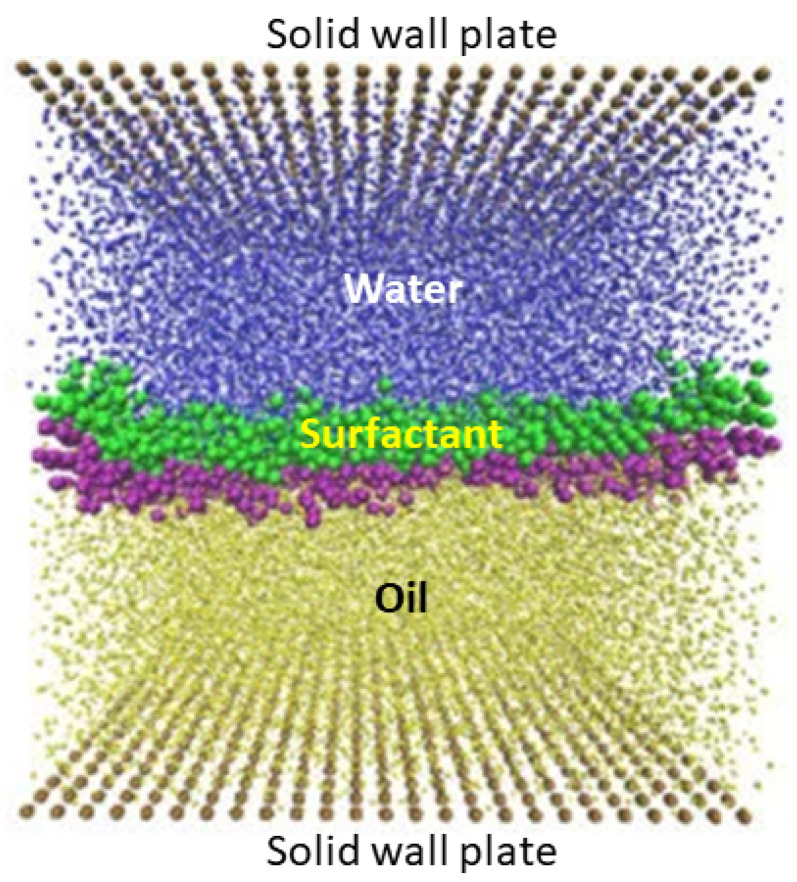
Simulations of a surfactant at the water–oil interface with 50% of water and oil between two solid walls under Poiseuille flow. As is apparent, all the surfactant molecules, representing sodium dodecylsulfate and octaethylene glycol monododecyl ether, remained at the water/oil interface with the hydrophilic head (green) beads facing toward the aqueous phase, whereas the hydrophobic tail (purple) beads tended to face the oil phase (adapted from [33]).

Surfactant at water–oil interface: The physical phenomenology taking place at the interface between immiscible fluids is essential to understand the behavior of the systems in the presence of oil–water or oil–gas flows. Applications range from multilayer coating and phase separation to industrial oil mobilization. Coarse-grained modeling methods, such as DPD, are suitable to simulate multifluid systems on large spatial and temporal scales, which are much larger than those achieved using MD simulations [30,31,32]. In [33], DPD methods are employed to study the flow of oil and water in a narrow slit under different flow conditions, in which large surfactant molecules can be included in the simulations (see Figure 3). In the case of Poiseuille flow, a critical shear rate occurs beyond which the surfactants desorbed, forming micelles and thus destabilizing the interface, while under a Couette flow, they remain stable over the whole range of shear rates studied.

Polymer crystals: Arrays of homopolymers ordered in a crystalline structure are relevant to a number of physical and chemical systems such as solar cells, semiconductors, biological materials, or conventional plastics. Understanding polymer crystallization is still controversial despite the large amount of research performed over the years [34,35,36,37]. In [38], the authors present the results of large-scale Monte Carlo (MC) simulations based on a system of 54 chains of 1000 monomers (hard spheres) each by developing an MC method that is able to correctly sample polymer conformational space in an efficient fashion. This is particularly hard to achieve for very long chains at very high concentrations. The simulations start from a purely random configuration, which rapidly develops into an hexagonal closed packed (HCP) polymorph and is followed by a random hexagonal close packed (rHCP) morphology. The latter finally goes into a stable face-centered cubic (FCC) crystal of very high perfection. In [39], the authors also studied local and global order in dense packed linear, semiflexible polymers of hard spheres by extensive MC simulations as a function of volume fractions (see also [40,41,42]). They analyze the way in which packing density and chain stiffness affect the ability of the chains to self-organize at short and long length scales. The critical volume fraction for the phase transition is determined, and the different crystal morphologies are obtained, yielding a complete phase diagram as a function of packing density and equilibrium bending angle.

**Figure 4:**
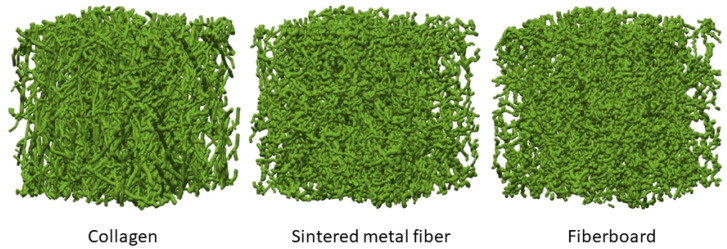
Simulations of fibrous materials. Shown are three cases with increasing (from left to right) tortuosity, using von Mises–Fisher directional probability distribution functions of random walks, aimed at modeling: Collagen, sintered metal fiber, and fiberboard, respectively (adapted from [47]).

Fibrous materials: Modeling fibrous materials is based on the available statistical databases of their structure, often obtained in the form of 2D distribution functions, to predict actual 3D fiber orientations (see, e.g., [43,44,45,46]). In [47], the authors suggest new methods to generate more accurate 3D fiber orientation probability distributions from 2D data and identify a way to determine suitable parameters entering the Mises–Fisher random walks approach (Figure 4). The new method succesfully accounts for the observed projection distortion and orientation distributions in real fibrous materials. The problem of dealing with fiber–fiber overlaps, emerging when the fibers are randomly generated in the simulation domain, is considered, and efficient solutions are suggested to alleviate it.

Anomalous dynamics: Polymer melts often display short-time anomalous dynamics behavior in obvious contrast to their single-molecule counterparts (see, e.g., [48,49,50,51]). In [52], short homopolymer chains are considered by studying MD simulation chain trajectories in a melt. Microscopic molecular mechanisms yielding the observed anomalous behavior are discussed in terms of subdiffusive dynamics obtained from the analysis of van Hove distribution functions and other structural properties.

**Figure 5:**
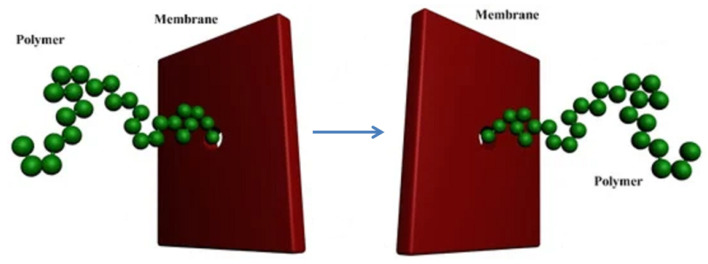
A linear polymer attempts to cross a membrane (from left to right) through a small pore, typically of nanometer size (adapted from [56]).

Translocation through a nanomembrane: Linear polymers can cross nanometer-sized pores when an external force is applied [53,54,55]. This translocation problem is relevant for understanding transport through nanomembranes, where typically the polymer moves under the presence of an electrostatic potential (see Figure 5). In [56], an analytical expression describing polymer flux through a nanopore is derived by assuming a linear dependence of the friction coefficient on the number of chain monomers *N* attraversing the pore and a parabolic behavior for the open-free (Gibbs) energy.

**Figure 6:**
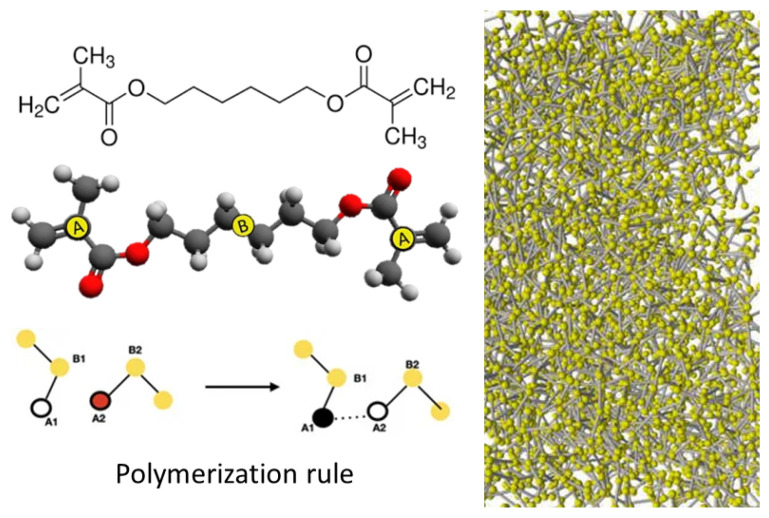
Polymerization of 1,6-hexanediol dimethacrylate (HDDMA) aimed at studying the gel-point transition. (**Left panel**) Structural formula of HDDMA (top image), molecular structure (middle image), and polymerization rule (bottom image). In the latter, a new bond is created between the atoms (A1, A2) if a reaction occurs, and two angle potentials, (B1-A1-A2) and (A1-A2-B2), are added to the chain, where the reactive atom is drawn in red and the potentially reactive one in white, while the inactive one is drawn in black. (**Right panel**) Snapshot of the polymeric structure for the initial radical concentration of 3% (adapted from [57]).

Polymerization (HDDMA): The radical polymerization process of acrylate compounds is studied in [57] to investigate the gel-point transition of 1,6-hexanediol dimethacrylate (HDDMA) [58,59,60] using a coarse-grained force field that reduces significantly the computer time. This model allows the simulation of much larger system sizes and smaller radical concentrations than in previous works (see Figure 6). Polymerization is modeled using reactive classical MD extended upon the implementation of a non-equilibrium MD scheme. A full cluster analysis of the resulting network substructures is performed, which is in good agreement with previously published all-atom calculations.

**Figure 7:**
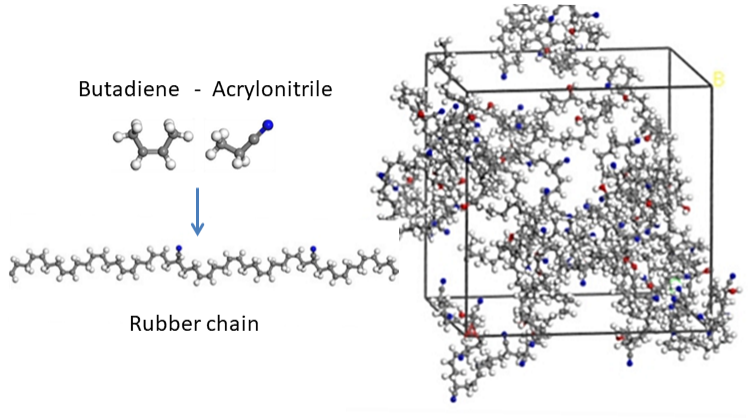
Molecular dynamics simulations of the formation of rubber. (**left**) The two molecular units, butadiene (**left**) (C brown sphere, H white sphere), and acrylonitrile (**right**) (N blue sphere), which are allowed to polymerize to yield a rubber chain. (**right**) A full optimization of a system of 5 chains, containing 65% of butadiene molecules of 50 units each, modified with hydroxyl groups (OH-) (O red sphere). The cubic system obeys periodic boundary conditions (PBC) and has a cell size of about 30 Å (adapted from [65]).

Nitrile butadiene rubber: Nitrile butadiene rubber (NBR) has excellent oil resistance and mechanical properties, being used to seal hydraulic systems. The problem arises when NBR deteriorates after undergoing thermal cycles, the action of oxygen and other chemicals, in addition to mechanical stress, leading to a loss of elasticity with deleterious consequences to the container working conditions. To gain insight into NBR aging (see, e.g., [61,62,63,64]), MD simulations have been implemented [65], using well-known force field (COMPASS) potentials [66] (see also [67]), where hydroxyl groups and carbonyl groups are introduced into the rubber molecular chains that mimic oxidative aging (see Figure 7). It becomes clear that elevated temperatures can dramatically increase the mobility of rubber molecular chains and fractional free volume, while strain compression results in further packing of the chains. A comprehensive picture thus emerges that helps understanding the effects of oxidative aging on rubber materials at the very molecular level.

Liquid wood: One of the major players to environmental pollution comes from the construction industry, mainly due to its fossil-fuel consumption and in loco production of particulate pollution. A possible solution would be using new materials adhering to environmental requirements, such as being of natural origin and therefore biodegradable [68,69,70]. In [71], the authors study the polymeric biocomposite commonly known as ‘liquid wood’ (LW), which is a good candidate for that purpose, and in addition, it is expected to be used for the restoration of degraded wooden moieties in civil structures, such as historical buildings, monuments, etc. Computer simulations are performed to study the mechanical behavior of LW by assuming that structure and functionality can be well described by a multifractal object, possessing different scaling properties at different spatial scales. A comparison with experimental data is discussed in support of the theoretical approach.

**Figure 8:**
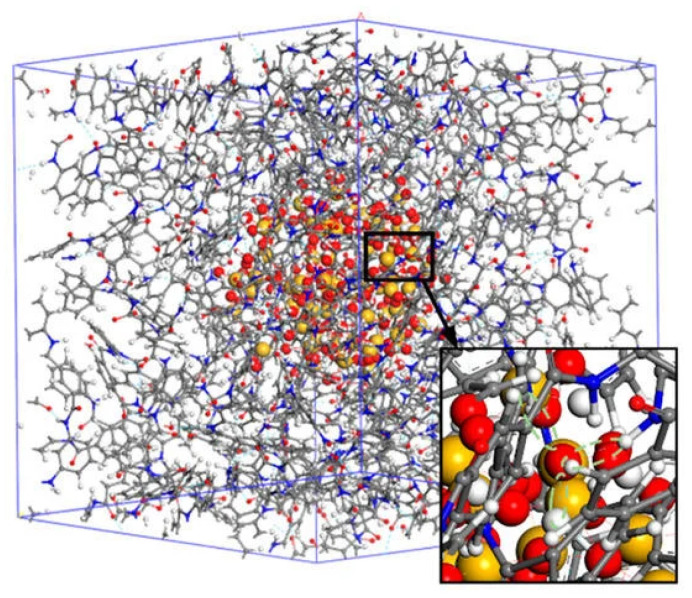
Snapshot of the simulations of poly-m-phenyleneisophthalamide (PMIA) network (C brown dots, H white dots, O blue dots), containing nano-silica particles (${\mathrm{SiO}}_{2}$) (Si yellow spheres). The inset shows a local zoom of hydrogen bonding in the ${\mathrm{SiO}}_{2}$/PMIA model (adapted from [74]).

Aramid/${\mathrm{SiO}}_{2}$ mixtures: Aramid, meta-aramid, and aramid-mica papers play a role in building insulating materials [72,73]. In [74], mixtures of poly-m-phenyleneisophthalamide (PMIA) with ${\mathrm{SiO}}_{2}$ (silica) nanoparticles (NP) have been studied by MD simulations (see Figure 8). Among these papers, PMIA is commonly used due to its suitable characteristics, but the range of applicability is limited by its rather poor thermal conductivity (see also [75]). It is indeed found that the thermal expansion coefficient of the ${\mathrm{SiO}}_{2}$/PMIA mixture decreases, both in the glass and rubber states, while the thermal stability is increased, suggesting that the addition of silica NP improves the thermal and structural properties of PMIA by apparently hindering the chain’s mobility.

**Figure 9:**
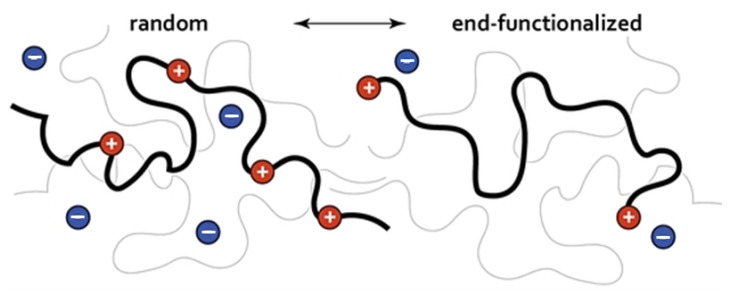
Mixtures of linear positively charged (red circles) polymers (linear segments) and mobile negative counterions (blue circles). The **left panel** illustrates the case of randomly charged polymers, while in the case depicted on the **right panel**, the polymers are charged only at their ends. The gray thin lines represent similar linear polymers present in the mixture (adapted from [79]).

Charged polymers: The mechanical properties of charged polymers can vary quite significantly depending on the way they become charged, but the microscopic mechanism responsible for the observed correlations between structural and dynamical properties remains largely unknown [76,77,78]. In [79], the authors study by means of all-atom MD simulations the structure and diffusion behavior of both randomly and end-functionalized ionic poly(dimethylsiloxane) (PDMS) melts in the presence of bromide (${\mathrm{Br}}^{-}$) counterions dispersed in the melt (see Figure 9), finding that the density of the ionic PDMS melts is larger than for neutral melts. The counterions’ main role is to slow down PDMS diffusion, while the counterion structure has a weak temperature dependence displaying an Arrhenius type of diffusion coefficient. The results contribute to our theoretical understanding of the correlations between the structure and dynamical properties of ion-containing polymers (see also [80]).

Bioplastics: The large accumulation of plastics waste in the global environment is endangering both wildlife and human health worldwide [81,82,83,84]. The biocompatible polymer polyethylene vanillate (PEV) is considered a good candidate to replace the widely used polyethylene terephthalate (PET), reaching us in our daily lives. In [85], the authors compare the structural dynamics and physical data of both plastics using all-atom MD simulations of a single polymer chain made of N=100 monomers. It is found that PEV flexibility is larger than for PET, predicting glass transition temperatures $T_{g}$ of about 353 K and 345 K, respectively, which is consistent with current experimental evidence. In addition, the work suggests that both plastics share similar physical and chemical properties enabling the replacement of PET by its bio-counterpart.

**Figure 10:**
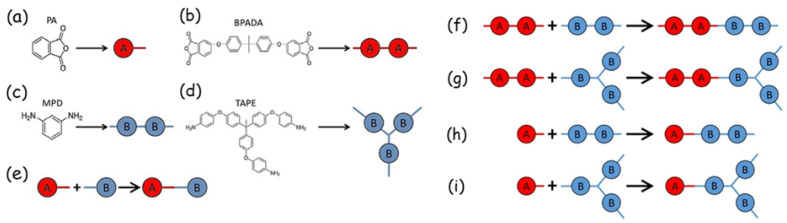
(**a**–**d**) The four types of monomers constituting a branched polyetherimide (PEI), used as models in the MC simulations, include: phthalic anhydride (PA), 4,4′-bisphenol A dianhydride (BPADA), m-phenylenediamine (MPD), and tris[4-(4-aminophenoxy)phenyl] ethane (TAPE) (the latter containing three ${\mathrm{NH}}_{2}$ terminals). The B bead represents a full functional group containing one amine, and the A bead represents a functional group containing a carboxylic anhydride. (**e**) The condensation reaction between an amine group and a carboxylic anhydride one, taking place in the polymerization of PEIs, is represented by the formation of a bond between beads A and B. (**f**–**i**) The polymerization of branched PEIs is represented by the following four reactions: (**f**) BPADA + MPD (see (**b**,**c**) above); (**g**) BPADA + TAPE (see (**b**,**d**) above); (**h**) PA + MDP (see (**a**,**c**) above); (**i**) PA + TAPE (see (**a**,**d**) above) (adapted from [89]).

Branched polymers: The molecular weight distribution (MWD) and configurational architecture are two important indicators of polymer characteristics. Indeed, they have a strong impact on material properties such as the dynamic modulus, fracture resilience, gel and glass transition temperatures, and viscosity [86,87,88]. MC simulations have been implemented [89] to efficiently deal with the challenging problem of obtaining the MWD of the branched polymer polyetherimide (PEI), which was formed via a step-growth polymerization (see Figure 10). The MC simulation results are compared with Flory–Stockmayer theory predictions, showing that the latter works well only below the gel point. The MC method can be used to accurately predict MWD in all cases, becoming a promising technique to deal with polymeric materials at the macroscopic level.

**Figure 11:**
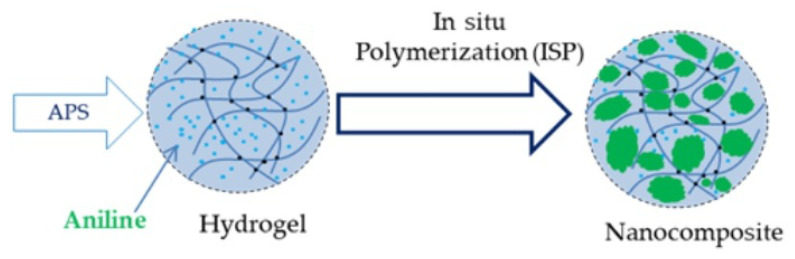
Illustration of the polymerization of aniline. Aniline molecules (green dots) are located inside a porous hydrogel, and by application of an oxidant (e.g., APS [90]), they start aggregating (larger green particles) by the process known as in situ polymerization (ISP), yielding a nanocomposite (adapted from [90]).

Synthetic hydrogels: The production of synthetic hydrogels is mainly based on the use of cross-linked polyacrylamide (cPAM) materials, keeping with their simple aqueous synthesis, great chemical stability and reproducible hydrogels formation (cf. Figure 11).

Used in the oil industry as a chemical method for water control, cPAM enhances crude oil flow by acting on water, which would otherwise block oil flow; thus, oil recovery can be significantly improved [91,92,93]. In [90], the authors consider hydrogels made of cPAM and conducting materials obtained from polyanilines (PANIs). Combinations of these two materials produce composites showing enhanced properties, intermediate between those of cPAM and PANIs. Typically, a gel composite is formed by radical polymerization, which is followed by incorporating the PANIs into the network by the oxidative polymerization of anilines (Figure 11). Such composites find a variety of technological applications, e.g., in photothermal/electromechanical actuators, supercapacitors, and motion detection sensors.

### 2.2. Polymers in Physics: Structural Properties, Self-Assembly, Smart Materials

Block copolymers: Block copolymers (BCPs) are attracting a great deal of attention due to their self-assembling characteristics, with potential use in the realm of nanopatterning, as they can yield a wide range of nanostructures well below the optical lithography resolution limit [94,95,96]. Self-assembly is a sort of microphase separation taking place due to the amphiphilic [28,29] nature of BCPs, which in combination with liquid phase infiltration (LPI) processes can open a path to the creation of novel nanomaterials with particular functional features. In [97], the authors provide an overview on the mechanisms underlying LPI processes and the role the associated infiltration parameters play in determining the nanomaterial properties. A bunch of applications in the field of photonics, plasmonics and electronics are comprehensibly highlighted.

**Figure 12:**
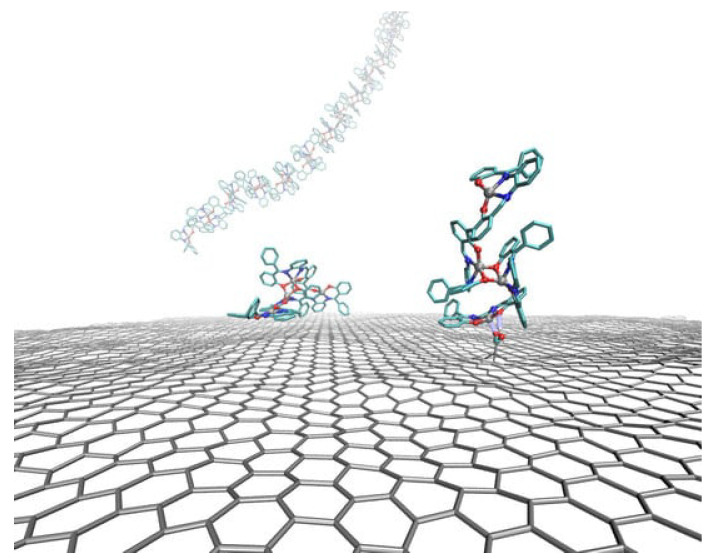
Complex polymer chains interacting with graphene (brown network). The colors in the polymer chains represent: N (Blue), O (red), C (green), Zn (brown) (adapted from [101]).

Bis-salphen chains on graphene: Molecular self-assembly is becoming a powerful tool to easily and accurately obtaining nanopatterns on very large length scales [98,99,100]. In [101], the authors apply density functional theory (DFT) and MD simulations to study the self-assembly of bis-(Zn)salphen compounds which interact with both pristine and functionalized graphene (see Figure 12). They demonstrate how graphene sheets, and also carbon nanotubes (CNTs), can act as condensation centers of bis-salphen chains, which intially stick to the carbon-based large exposed surface, followed by a translational motion along the surface favored by their weak interactions with π–π carbon dangling bonds. The process ends when they become entangled with other chains, leading to their self-assembly. For other applications of graphene-related material interfaces, see the review in [102].

**Figure 13:**
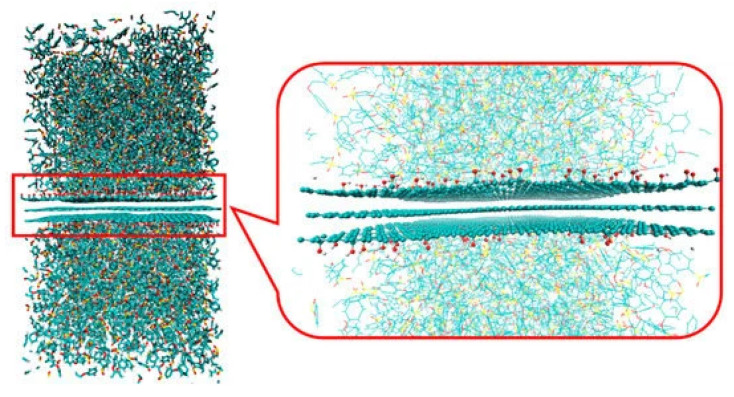
Snapshot of an ensemble of PEI chains (green dots) near a functionalized graphene sheet (dark green) containing -OH dangling bonds (red dots) fixed at random locations on the carbon structure (adapted from [103]).

Polymer/graphene interfaces: In [103], the authors use MD simulations to study the stability of thermoplastic polymer/graphene oxide interfaces. For the former, they consider polyethersulfone (PES) and PEI, while graphene is oxidized in different ways, finding that PES becomes strongly stabilized in the opposite fashion as PEI does (see Figure 13). It is concluded that the observed different orders of stability are due to a balance between electrostatic interactions, in which atoms attract each other under a strong charge bias, thus stabilizing the interface, while excluded-volume effects between functional groups destabilize the interface as they prevent the π−π stacking of aromatic rings [104,105,106].

Knoted polymer chains: In [107], the authors consider the issue of knoted polymer chains. This phenomenon is actually quite common in our daily life, but it is also relevant to polymer chains at the microscale, in particular in the case of biopolymers such as DNA and proteins, as well as for synthetic polymers. They combine MD simulations for a single polymer chain with multiparticle collision dynamics (MPCD) for solvent molecules to study the interesting problem of untying a trefoil knot polymer chain, which translocates through a narrow channel. This study may shed light into the translocation behavior of knotted polymer chains through a capillary (see also [108,109,110,111,112]).

Porous polyimide: In [113], the authors review properties of porous polyimide (PI), known for its applications as an interlayer insulating material displaying low dielectric constant, κ, aimed at improving transmission speeds between chips in large-scale integrated circuits. Several physical and chemical methods for reducing the dielectric constant of PI-based materials are mentioned and discussed. The work emphasizes key technical problems and challenges encountered in optimizing porous polyimide materials aimed at pushing the lower limit for κ even further (see also [114,115,116]).

Additive manufacturing: Thanks to recent advances in material synthesis and additive manufacturing (AM) technologies (see, e.g., [117]), the advent of metamaterials, known also as lattice materials since they are based on cellular architectures, has become possible. Inspired by nature, they possess a variety of multifunctional attributes [118,119,120]. In [121], the authors review a collection of recent developments on promising polymeric metamaterials (PMMs). The review covers different areas such as the design, fabrication and testing of PMM, stressing the need for their timely developments similarly to the ones carried on triply periodic minimal surfaces (TPMSs) lattices. This point of view extends to future research on the fabrication of PMMs under complex loading scenarios in order to understand their behavior. The achievement of this goal can be greatly facilitated by relying on machine learning algorithms to alleviate many of the current difficulties encountered in the fabrication of PMMs using AM techniques.

Conjugated polymers: Conjugated polymers have undergone large developments as organic semiconductors, competing with silicon-based inorganic materials which have dominated the semiconductor industry over the years [122,123,124]. In [125], the authors review several properties of conjugated polymers related to their structures, glass transition phenomena at $T_{g}$, and crystallization scenarios, which is aimed at understanding their relationships for optimizing their performances. For instance, it is known that in conjugated polymers, side-chain interactions affect $T_{g}$. Also, their growth processes and crystallization paths have an impact on mechanical and electrical properties. The review provides a thorough discussion on advanced characterization techniques such as X-ray diffraction, atomic force microscopy, and thermal analysis, helping to understand how molecular ordering and polymer–crystal interfaces take place. It lays a firm conceptual foundation for further research and development on conjugated polymer-based materials.

Block copolymers and brushes: Self-consistent field theory (SCFT) is a powerful theoretical technique to deal with a many-body system in which the complex interactions scenario is treated within a mean-field approach [126,127,128]. In [129], the authors introduce a simple 3D SCFT algorithm based on real-space methods with adaptive discretization, which increases the accuracy and efficiency of the numerical calculations, and apply it to the problem of polymeric materials surfaces. The algorithm performance is tested on two very distinct polymeric systems: block copolymer (BCP) [97] films and polymer brushes (see also [22]). The key point is the use of a finer contour discretization at grafting chain ends, thereby increasing spatial resolution in regions influenced by external forces, yielding one of the most accurate SCFT treatments of 3D polymeric systems so far.

**Figure 14:**
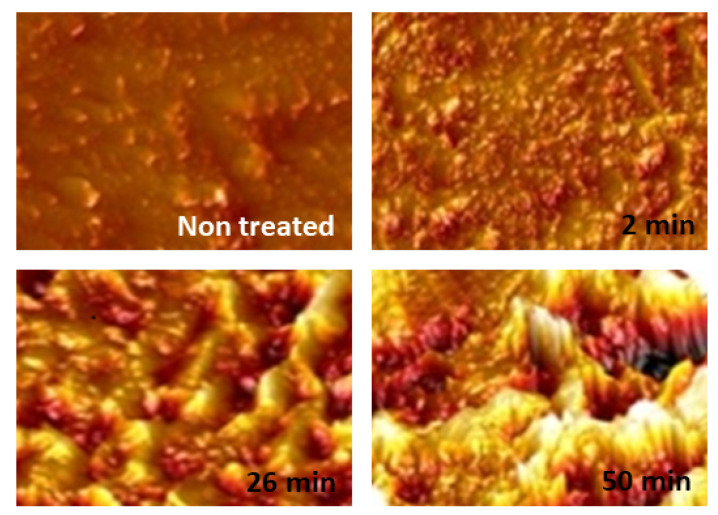
Images of polytetrafluoroethylene (PTFE) surfaces obtained using atomic force microscopy (AFM). The PTFE surfaces have been treated with a radio frequency plasma torch for different exposure times as indicated in the figure: 0 min (original surface), 2 min, 26 min, and 50 min. The resulting root-mean-square roughnesses are 22 nm, 33 nm, 68 nm, and 150 nm, respectively (adapted from [135]).

Plasma techniques: We close with a brief discussion on plasma techniques, specially in view of their applications to polymeric materials [130,131,132,133,134]. In [135], the author presents a historical overview of the development and achievements of cold plasma technology (see Figure 14). The study includes a variety of plasma sources such as low-pressure glow discharges and atmospheric pressure plasmas used for dielectric barrier devices. Key operational parameters such as pressure, input gas type and flow rate, applied electric field, and type of discharges are critically examined. The review also discusses applications of cold plasmas to polymeric materials surface modifications and associated properties such as wettability and adhesion. A number of plasma techniques are considered which are relevant to polymeric materials and films. Further applications cover the biomedical sector and the crucial role of plasmas for water purification, gas separation, and energy production. It includes applications to bioplastics and the possibility of developing self-healing materials using this useful technology. A recent book summarizes some of the most important applications of low energy plasmas and the more advanced plasma-assisted supersonic jet deposition (PASJD) technique [136].

### 2.3. Polymers in Chemistry: Structural Properties and Solubility Issues

Polymer electrolyte for fuel cells: Market emerging electric vehicles partially rely on polymer electrolyte membrane fuel cells (PEMFCs) with an increased use of alternative sulfonated hydrocarbon-based proton exchange membranes (PEMs) [137,138,139,140], as they largely overcome many of the drawbacks associated with perfluorosulfonic acid polymers. In addition, PEMs have higher chemical and mechanical stability and significantly lower production costs. In [141], the authors employ MD simulations to study the effects of side chains length in sulfonated polystyrene grafted poly(arylene ether sulfone)s (SPAES), which are used as proton exchange membranes, where side chains length improve proton transport in hydrophilic environments.

Nitrile butadiene rubber: NBR is a non-natural rubber obtained from the synthesis of acrylonitrile (ACN) and butadiene ($C_{4}$$H_{6}$). It is mainly used for fabricating seals in general and hoses to transport fuel/oil in the aeronautical/automotive industry, since it has a wide temperature range of stability (−40 °C to 108 °C) (see also Figure 7 and [65]). The development of new bio-gasoline/diesel fuels requires a careful examination of NBR response to different structural and chemical stress factors [142,143,144]. In [145], the authors investigate the swelling response of NBR to different acrylonitrile (ACN) contents to validate a prediction model of its oil-resistant features. They base their analysis on a modified Flory–Huggins interaction parameter $\chi_{\mathrm{HSP}}$, being a function of the Hansen solubility parameter (HSP). The NBR swelling response can be evaluated quite accurately, suggesting that the new model can be used to keep the oil resistance under control even in extreme application conditions.

**Figure 15:**
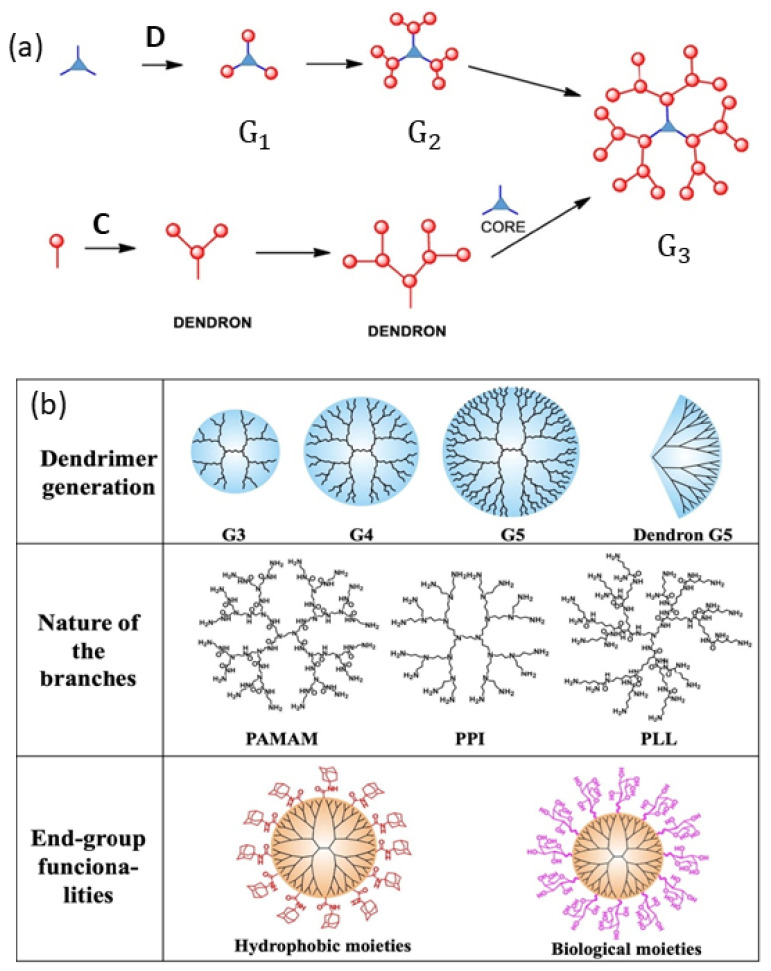
(**a**) Illustration of a divergent synthesis (first two steps) of dendrimer $G_{3}$ from dendrimers $G_{1}$ and $G_{2}$ (D-sequence) and a convergent path via dendrons formation (C-sequence) (adapted from [150]). (**b**) The main parameters determining the structural properties of dendrimers [151].

Dendrimers: Dendrimers consist of a central core molecule from which many branches can emerge yielding a hierarchical topology, and they constitute a prominent category of synthetic materials [146,147,148,149]. They possess a structural organization with a globular morphology at the nanoscale which reminds us of proteins (see [150] and Figure 15a).

In [151], the authors review the properties and applications of quintessential dendrimer varieties such as polyamidoamine (PAMAM), poly(propylene imine) (PPI), polylysine (PLL), and polyester dendrimers (see Figure 15b). The focus of the work lies in the discussion of the essential properties and applications of these types of dendrimers, which are widely used today.

### 2.4. Polymers in Chemistry: Quantum Mechanical Calculations and MD Simulations

Pyrolysis for polystyrene: The exuberant production and uncontrolled consumption of plastics are causing severe, apparently irreparable damages to all forms of life on Earth. Pyrolysis turns out to be a possible solution for waste management, including recycling, landfill disposal and combustion [152,153,154]. In [155], the authors report the experimental development, and mathematical modeling, of a new thermal pyrolysis process for polystyrene (PS). The theoretical model significantly improves on previously discussed approaches by solving the full MWD without ad hoc assumptions, yielding a more complete understanding of the pyrolysis process.

Oilfield scales inhibitors: The unsolicited aggregation of different materials within the main working parts in an oil or gas production system, commonly referred to as scale deposition, is a major issue in the industry of energy commodities. Typically, oilfield scales contain calcium carbonates and sulfates, including barium sulfate and iron sulfides, among which calcium carbonate (${\mathrm{CaCO}}_{3}$) is one of the main components [156,157,158]. In [159], the authors employ DFT and ab initio molecular dynamics (AIMD) techniques to study the performance of scales inhibitors, such as polyacrylamide (PAM) and its silica functionalized counterpart (PAM-Silica). The latter was found to be a suitable calcium scale inhibitor due to its high binding affinity with ${\mathrm{Ca}}^{2+}$, suggesting that it could be used as an efficient scale inhibitor.

**Figure 16:**
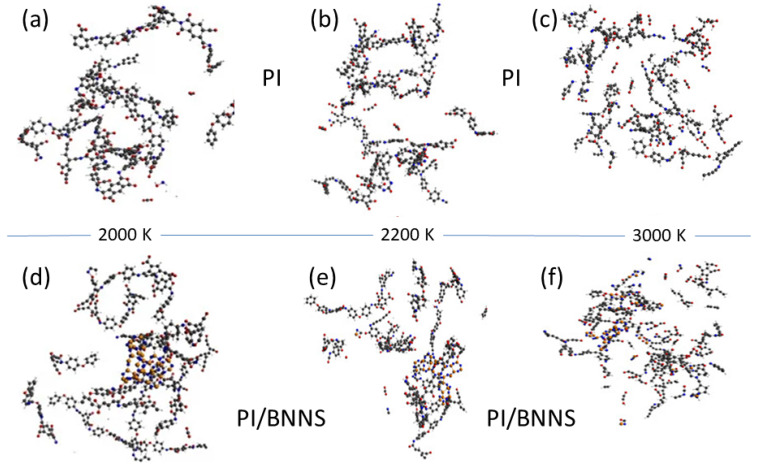
Molecular dynamics simulations of pyrolysis. (**Upper panel**) PI neat: (**a**) 2000 K, (**b**) 2200 K, (**c**) 3000 K. (**Lower panel**) PI/BNNS: (**d**) 2000 K, (**e**) 2200 K, (**f**) 3000 K (adapted from [164]).

Insulating materials: As discussed in Section 2.2, PI is widely used as an insulating material (see also [113]). In many applications, high temperatures are involved which require the material to maintain a robust thermal stability and good thermal conductivity [160,161,162,163]. In [164], the authors show that certain arrangements of boron nitride nanosheets (BNNSs) with polyimide (PI) can yield insulating materials with high thermal conductivity. They support their conclusions with extensive MD simulations showing the inhibitory role played by BNNSs on the decomposition process of PI (see Figure 16), which can be caused by different molecular entities.

### 2.5. Polymers in Chemistry: Environmental Issues, Batteries, Multifunctional Materials

Gas desorption of $H_{2}$ from polymer media: Hydrogen gas is attracting a great deal of interest nowadays as an environment-friendly energy source, because it can be produced from clean energy suppliers such as solar, wind, hydro, and nuclear technologies. The ever-increasing need for the use of hydrogen fuel requires the development of newly designed infrastructure for its handling, storage and transportation, and polymer materials are widely employed for these purposes. Indeed, a hydrogen tank liner provides a safe storage space to contain and protect the hydrogen gas stored inside a cylinder, ensuring a high level of containment preventing hydrogen to leak or escape out in the environment. Polyamide, polyethylene, and/or cross-polyethylene, build the innermost part of the liner. All these polymers display minimal permeability for the highly volatile hydrogen gas, thus providing the container with a very good barrier that minimizes possible gas losses during its storage. Since the operating conditions require high-pressure hydrogen gas, a rapid decompression event outside the liner can yield to gas desorption during which hydrogen diffuses rapidly out of the container, producing blisters or cracking that may cause a permanent damage to the polymer [165,166,167]. In [168], the authors study numerically the diffusion of hydrogen through a polymer material for storages of different geometries such as cylindrical, spherical and sheet-shaped containers. They quantify their results in terms of the desorption equilibrium time, representing the time for which $H_{2}$ diffusion loss attains its saturation level, displaying an exponentially growing trend as a function of the square of both thickness and diameter of the cylinder-shaped specimen, while it was found to be proportional to the diameter square for the sphere-shaped specimen and to the thickness square for the sheet-shaped specimen. The model calculations based on solving the diffusion equation are in good agreement with the experimental results, thus providing a useful tool for predicting the time-dependence of $H_{2}$ desorption in polymer materials.

Biodegradable polymer materials: To mitigate the burden of waste management due to the exponential growth of anthropogenic causes of environmental pollution, new regulating policies are encouraging the more extensive use of biodegradable materials [169,170,171,172]. In [173], the authors review recent developments within the topic of biomaterials. They consider in particular the prominent role played by polycaprolactone (PCL) as one of the best candidates, whose use has increased at a rapid pace in recent years. Indeed, PCL-based biocomposites, reinforced by adding NP, display interesting mechanical and thermal properties relevant to many applications.

Bio-synthesizable, biodegradable polymers: Petroleum-based synthetic plastics are designed to possess good performance and durability. Unfortunately, these properties are in hard conflict with the new requirements of biodegradability and recyclability [174,175,176,177]. In [178], the authors determine the mechanical properties of polyhydroxyalkanoates (PHAs) and their relation to chemical behavior using MD simulations. PHAs have lately emerged as very promising bio-synthesizable, biocompatible, and bio-degradable polymers expected to replace petroleum-based plastics. The work suggests rules for tailoring the mechanical properties of PHAs, opening the door to future theoretical studies aimed at identifying functional PHA polymer candidates for targeted applications.

**Figure 17:**
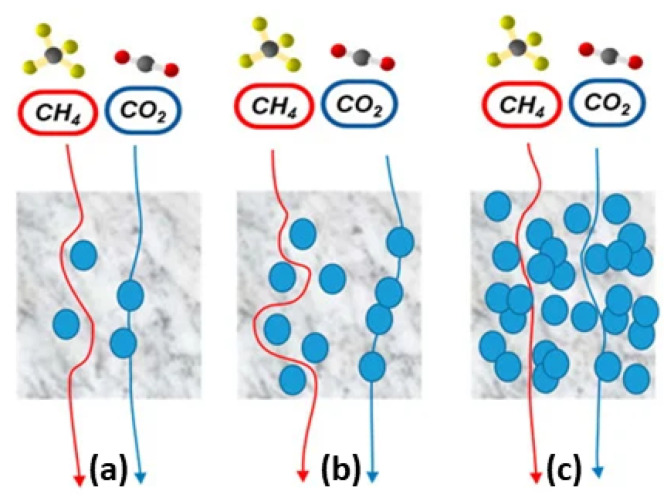
Fillers, represented by the full blue circles, dispersed in MMMs. (**a**) Very low filler contents may not achieve the desired selection effects. (**b**) At intermediate filler concentrations, one expects to find an optimal filler behavior. In many cases, very low filler contents are actually sufficient to sharply modify the polymer matrix characteristics. This aspect is rather encouraging for large-scale separations at still competitive costs. (**c**) At too high filler concentrations, one reaches a threshold value leading to particle agglomeration, where the MMMs display a reduced permselectivity [185] (adapted from [183]).

Gas separation membranes: Membrane technology has seen an upward trend in recent years due to its simplicity and lower costs than its peers, particularly regarding gas separation. Certainly, polymers occupy a prevailing role among the different materials constituting the membranes. Gas separations involving ${\mathrm{CO}}_{2}$ are an essential requirement to cope with the greenhouse effect and with the aim of pursuing a model of sustainable growth [179,180,181,182]. In [183], the authors review the use of Pebax polymers, i.e., block copolymers (see also Section 2.2 and [97,129]) made up of rigid-polyamide and soft-polyether blocks, for developing Pebax-based mixed-matrix membranes (MMMs), in which inorganic fillers are typically added in different contents (see Figure 17, and also the similar case of NP dispersed in ionic conductors [184], which may be relevant to MMMs). These promising fillers may yield new innovative membranes with improved performances.

Polymer electrolytes in proton-based batteries: Polymer electrolytes (Section 2.1 and [28]) are expected to have further impact on the development of advanced electrochemical devices like batteries, supercapacitors, fuel cells, solar cells, sensors, etc. [186,187,188,189]. In [190], the authors review the ever-expanding use of polymer electrolytes in proton-conducting batteries, which has become a prominent research field yielding the creation of new and more complex polymer-electrolyte materials, stressing the need for additional structural characterizations to further optimize these novel materials. The work reviews recent advancements in proton-conducting polymer electrolytes characterization for their use in solid-state batteries.

Polymer electrolytes for Li-based batteries: Lithium-ion batteries (LIBs) play such a dominant role in electric vehicles and portable devices applications due to their high energy density and long lifespan, but safety concerns remain, and their extremely high energy density is a limiting factor for developing new more efficient devices [191,192,193,194]. In [195], the authors review polymer electrolytes and the possibility of combining them with ionic salts, which has indeed produced significant advances in battery technology in addition to dealing with safety issues, increased capacity, and longer life cycles. The review also provides future perspectives for developing novel polymer electrolytes to be applied for high-performance Li-based batteries.

Epoxy resins: In [196], the authors review properties of epoxy resins related to their structural characteristics, chemical and electrical properties, and their modifications (see also [197,198,199,200]). Indeed, due to their multifunctional characteristics, they have become fundamental material components in different industrial sectors. The work discusses the mechanisms determining their dielectric breakdown behavior and new strategies to enhance the dielectric strength as well as the way fillers and additives have an impact on their insulation properties.

Polymer actuators: Nature-inspired polymer actuators, developed to mimic the real movements and functions of natural organisms, are expected to find applications in bio-medical engineering, soft robotics, and energy harvesting, since they can efficiently convert electrical, thermal, or chemical energy into mechanical motion [201,202,203,204]. In [205], the authors review recent progress in the development of electrospun actuators, commonly based on polymers such as stimuli-sensitive hydrogels, shape-memory polymers (SMPs), and electroactive polymers. The surveyed future polymer actuators scenarios can contribute to the search for more advanced and multifunctional systems with improved performance and new elaborated applications.

Three-dimensional (3D) printing for protheses: AM, commonly known as 3D printing (see Section 2.2 and [117,121]), has the potential to revolutionize future achievements in medical fields such as healthcare [206,207,208]. Indeed, this technology is able to reshape the manufacturing of prosthetics and prostheses, no matter how complicated, for a wide range of polymeric materials. In [209], the authors review applications of polymer and its composites in the medical sector using 3D printing technology, discussing the creation of tailored prostheses, anatomical models for surgical planning and training. The article also discusses applications for drug delivery systems (DDSs) and tissue engineering, in addition to stereo-lithography, fused deposition modeling (FDM), and selective laser sintering (SLS) techniques. It is expected that manufacturing new medical devices will lead to better healthcare services and improved patient outcomes.

### 2.6. Polymers in Chemistry: Rheological Properties, Viscoelasticity

Computational fluid dynamics: Fluids displaying viscoelastic behavior show both viscous and elastic responses to external stimuli [210,211,212]. Many industrial applications actually deal with viscous fluids, so the question arises of how to accurate characterize the latter in order to optimize the associated industrial operations. In [213], the authors implement a variety of theoretical models to describe complex rheological behavior using a recently developed computational fluid dynamics (CFD) software, which is able to simulate Newtonian, generalized-Newtonian and viscoelastic flows, based on finite differences on suitably defined hierarchical grids (HiGs). It is found that the new methodology implemented in a HiG system can successfully reproduce the rheological behavior observed in a variety of fluids of interest to the broad rheology community, in particular typical non-monotonic flow curves of micellar solutions and plug-flow velocity profiles of viscoelastic fluids with yield-stress.

Epoxy–matrix composites: Composite materials can be coveniently reinforced by adding suitable amounts of natural fibers, aimed at lowering production costs, material toxicity, and specific weight [214,215,216,217,218]. In [219], the authors study theoretically epoxy–matrix composites reinforced with randomly oriented Cuban henequen (also known as sisal or agave fourcroydes) long fibers to determine essential parameters describing uniaxial three-element viscoelasticity. Their analysis is based on a constitutive model, with both integer and fractional index derivatives, to understand the mechanical evolution of the composite at different fiber/matrix compositions, discussing several methods to estimate the parameters accurately. The predictions are consistent with the experimental data, yielding a new feasible and accurate strategy to describe the viscoelastic behavior of fiber/matrix composites.

**Figure 18:**
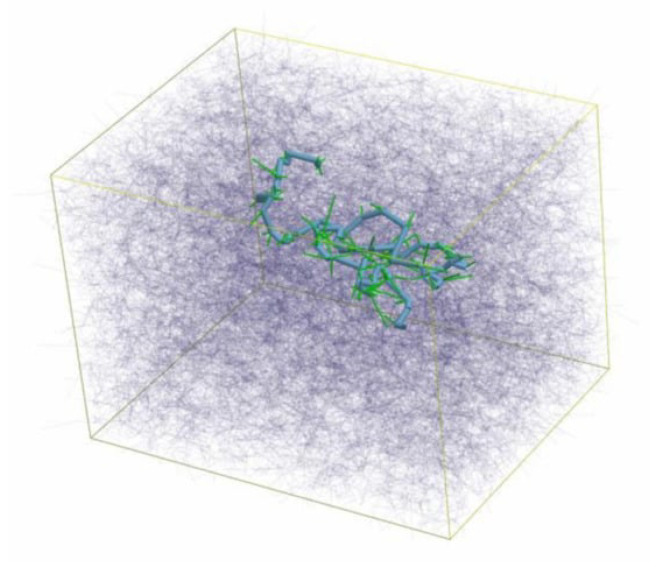
A multi-chain simulation based on the primitive chain network model to study the elongational rheology of polymers, such as polypropylene carbonate. A typical snapshot of a chain with N=88 units (green lines) is shown, while the thin black lines are the other chains. The thick green lines are segments entangled to the test chain. Periodic B.C. are used (adapted from [223]).

Elongational rheology: The nonlinear elongational rheology of polymers behaves differently than in the case of linear viscoelasticity and nonlinear shear rheology. According to tube theory (see Section 3), the elongational viscosity of entangled polymers should decrease with applied strain rate under specific elongation rate conditions [220,221,222]. In [223], the authors analyze the elongational viscosity of polypropylene carbonate (PPC) melts, using reptation dynamics for the single chains [224] (see Figure 18), and that of a polystyrene (PS) melt to estimate effects of chemistry, since the latter has an entanglement number per chain and a polydispersity index similar to PPC. The model uses slip-link networks consisting of strands, nodes, and dangling ends. Each path connecting two dangling ends corresponds to a polymer, which can go through strands and connected nodes. At each network node, one slip-link is located to connect two polymer chains in accord with the assumed entanglement rule. A 3D motion of the slip-links, the chain sliding through them, and their creation and destruction at the chain ends are considered, where the slip-link position is determined by solving a Langevin-type of equation. A constant monomeric friction overestimates elongational viscosity, therefore friction is allowed to diminish with increasing segment orientation. The effect of chemistry is studied by simulating PS, suggesting that PPC and PS behave similarly in terms of the reduction of friction under fast deformations.

Polymers rheology theories: The question how polymer chains do interact in a melt, and the ways they respond to shear deformations, specially at high temperatures, are some of the main aspects that need to be answered for understanding the behavior of polymer viscous liquids [225,226,227]. It is generally accepted that there is a characteristic molecular weight separating two different scenarios: the low polymer mass one consisting of un-entangled chains described by the Rouse model and the heavier polymer mass regime, where chains become strongly entangled and the de Gennes reptation model plays a major role (see, e.g., [228]). In [229], the authors examine both failures and challenges of this current paradigm, and they suggest possible theoretical concepts to move forward our understanding on polymer rheology.

Thermoplastic polyimides on C-nanotubes: It is known that the mechanical properties of thermoplastic semi-crystalline polyimides can be enhanced by the presence of single-walled carbon nanotubes (SWCNTs) (see, e.g., [230,231,232]). In [233], the authors perform all-atom MD simulations up to microseconds time scales to understand the behavior of thermoplastic semi-crystalline 4,4’-Bis(4-aminophenoxy)biphenyl (BAPB)-based compound polyimide R-BAPB near an SWCNT. They find an enhanced viscosity of the polymer melt in agreement with experiments. More importantly, the viscosity increase is related to the conspicuous structural ordering of the chains near the carbon nanotube, thus excluding the possible formation of new interchain links. As a result, a strong anisotropy in the rheological properties of the R-BAPB near the carbon surface emerges, which is attributed to the existence of the polyimide chains’ preferred orientations.

Orientational relaxation: Orientational relaxations of linear one-component polymer melts are well understood on the basis of Doi–Edwards–de Gennes tube-like models. The situation is, however, still controversial regarding relaxation phenomena in the case of a long linear polymer immersed within an ensemble of shorter chains (see, e.g., [234,235,236]). In [237], the authors discuss the relaxation of a self-unentangled long chain dispersed within a one-component polymer melt made of shorter chains, finding that while the long-chain relaxation is well approximated by a constraint release Rouse (CRR) mechanism, there is no unique prediction on the CRR relaxation time scale, $\tau_{\mathrm{obs}}$, representing the entanglement’s mean release time. The aim of the work is to discuss different approaches yielding $\tau_{\mathrm{obs}}$ and compare them to a large set of experimental viscoelastic data, including poly(methyl-)methacrylate and 1,4-polybutadiene blends, polystyrene and polyisoprene blends (see also [238]). It is found that $\tau_{\mathrm{obs}}$ grows as a power-law of the short chains molar mass with an exponent of about 2.5 instead of 3 as predicted by the CRR mechanism. They suggest a new description of $\tau_{\mathrm{obs}}$ implemented within a tube-like model consistent with the experimental observations.

### 2.7. Polymers in Biology: Macromolecules, Proteins, Structures and Dynamics

Large-scale molecular simulations: In [239], the authors explore the possibility of simulating molecular systems on very large scales. They remark the fact that whole micelles, bigger than 20 nm in size, formed by the self-assembly of hundreds of copolymers each containing as many as 50 units, have not been studied numerically so far. To overcome this lack of theoretical information, extensive MD simulations are performed on 900 flexible amphiphilic triblock copolymers made of 80 units each, aimed at describing both ribonucleic acid (RNA) and small interfering RNA (siRNA), the latter causing gene silencing through repression of translation in the cell [240,241,242]. Typically, siRNA is carried by a protein in the cell forming an RNA-induced silencing complex (RISC). The siRNA then unbinds from RISC and binds to its complementary messenger RNA (mRNA), silencing the gene that encodes it by inducing mRNA cleavage. It is concluded that such detailed MD simulations are possible and useful for the optimization of physicochemical characterizations of siRNA micelle complexes for improving the design of future drug delivery systems.

Actin filaments: Actin is the most abundant highly conserved protein in eukaryotic cells, participating in the majority of protein–protein interactions. More specifically, actin filaments (F-actins), resulting from the polymerization of the monomeric G-actin protein, are highly charged double-stranded rod-like polyelectrolytes, and they can be organized into higher-order structures, forming bundles and networks thus providing mechanical support [243], cell shape, and motion to the cell surface, which is essential for enabling the cell diverse functions [244,245]. In [246], the authors introduce a unique approach that combines dynamics and electrophoresis light-scattering experiments, with an extended semiflexible worm-like chain model built upon an assumed asymmetric distribution of polymer lengths, to characterize the polyelectrolyte and hydrodynamic properties of actin filaments in aqueous electrolyte solutions. The optimized models can be used to calculate other actin filaments properties such as stability, intrinsic viscosity, axial tension, elastic stretch modulus, and the force associated with the increase and compression of filaments length.

**Figure 19:**
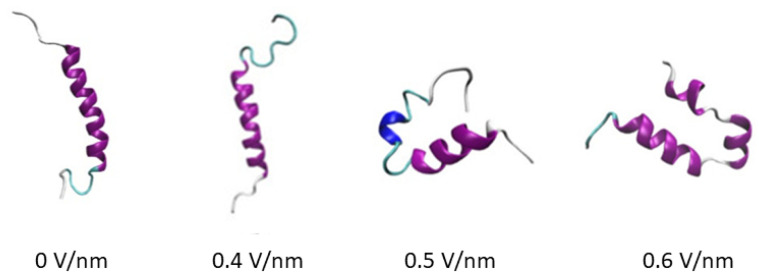
Three-dimensional structures of GLP-2 after MD simulations of 100 ns duration. Structures obtained by application of a uniform electric along the *z*-direction of intensity: (0, 0.4, 0.5, 0.6) V/nm, while for larger fields, the linear structure of the α-helix is restored (adapted from [250]).

Electric field effects on GLP-2 peptide: Incretins are hormones produced in the gut in response to food, stimulating glucose-dependent insulin release, such as glucagon-like peptide-1 (GLP-1) and glucose-dependent insulinotropic polypeptide (GIP). They are secreted by the so-called enteroendocrine K- and L-cells of the intestinal mucosal epithelium. The L-cells secrete glucagon-like peptide-2 (GLP-2) [247,248,249], which is a single-chain protein of 33 amino acids of 3.8 kDa total weight containing 264 atoms. In [250], the authors perform extensive MD simulations of GLP-2 to study the effects of electric fields on its conformation, finding that its stability is reduced by the electric field, transforming it into unstable turn and coil structural shapes (see Figure 19), but returning to the linear shape at higher fields. It is expected that MD simulations of the effects of electric fields on the GLP-2 structure can provide a useful theoretical basis for understanding the biological function of this important hormone in vivo.

**Figure 20:**
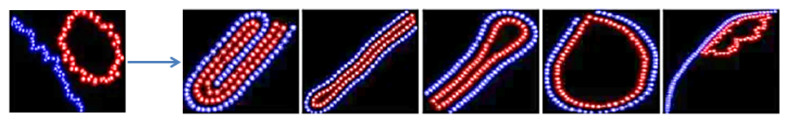
Interaction of a linear polymer chain (blue beads) with a vesicle, represented here as a polymer ring (red beads) in two dimensions, for different values of the bending stiffness (adapted from [254]).

Soft vesicle–polymer chain conformations: A vesicle is a complex biological macromolecule consisting of a liquid, or cytoplasm, enclosed by a lipid bilayer, and it is important for both biological and materials science processes, such as endocytosis via transformations of cell membrane shape, drug delivery, nanochemistry, and micro-reactors [251,252,253]. In [254], the authors employ MC simulations to investigate the interaction between a soft vesicle with a linear polymer (see Figure 20), yielding different types of induced vesicle conformational changes (see also [255]). The effects of polymer bending stiffness and strength of the assumed attractive interaction between vesicle and polymer are discussed. The results are expected to be useful for engineering future applications of such vesicle shape transformations.

Charged polymers: Our current understanding of the different thermodynamic phases of charged polymers in solution is still incomplete essentially due to the interacting multi-component nature of the system, such as long-range electrostatic and excluded volume interactions, the different components’ translational entropies, chain connectivity [256,257,258,259], etc. In [260], the authors study the different phases undergone by charged polymers in solutions containing polar molecules by developing a novel liquid-state (LS) theory with short-range interactions. Specifically, interactions between charged polymer groups with external counterions, in addition to those between neutral polymer parts and charged intrapolymer segments, are taken into account by means of an extra Helmholtz free energy within the framework of perturbed-chain statistical associating fluid theory (PC-SAFT). A computational scheme used for salt-free polymer solutions is developed, allowing easy computations of the binodal curve and critical points for different model parameters.

Chirality of polymer knots: Polymer knots are formed when a long polymer chain is looped on itself. They occur quite naturally in both synthetic and biological polymers, such as DNA and proteins. Knotted polymers are attracting increasing interest due to progress achieved in macromolecular synthesis, biology, mathematics, and molecular simulations [261,262,263]. In [264], the authors study coarse-grained DNA polymers pushed inside chiral and achiral open channels using MD simulations, and they investigate the polymer metrics in terms of their span, monomer distributions and topological changes in the channels. The compression affects the polymer topology differently in chiral channels than in achiral ones. The former yields equichiral knots with the same handedness as the channels one.

**Figure 21:**
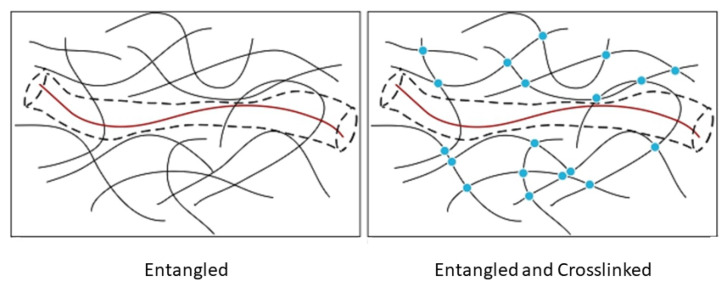
Schematic illustrations of (**left panel**) an entangled semiflexible polymer network of filaments (black lines) with an embedded tracer filament (red line). (**right panel**) In this case, the filaments (black lines) are either intertwined or connected by a crosslinker (blue circles). The dashed tubular structure indicates the space available to the tracer (red) filament, which is generally denoted as the reptation tube (adapted from [269]).

Entangled polymer networks: The tube model for polymer melts is widely seen as the reference scheme for understanding the complex behavior of flexible polymers solutions, as it reduces the many-body problem into a much simpler mean field-type approach, in which a test chain is constrained to move within a tube virtually constructed by the surrounding polymers. It has, however, a number of shortcomings such as the lack of thermal fluctuations of the tube itself, which are responsible for the observed disentanglement effects [265,266,267,268]. In [269], the authors report experimental evidence that correlated constraints release does occur in entangled polymer networks but not in their crosslinked semiflexible counterparts (see Figure 21). In their experiments, they track single semiflexible DNA nanotubes, which are very similar to F-actin filaments in both thickness and length, embedded both in entangled and crosslinked F-actin networks, suggesting that very different reptation dynamics take place in these systems. This work is of primary importance for future theoretical work on the disentanglement issue.

**Figure 22:**
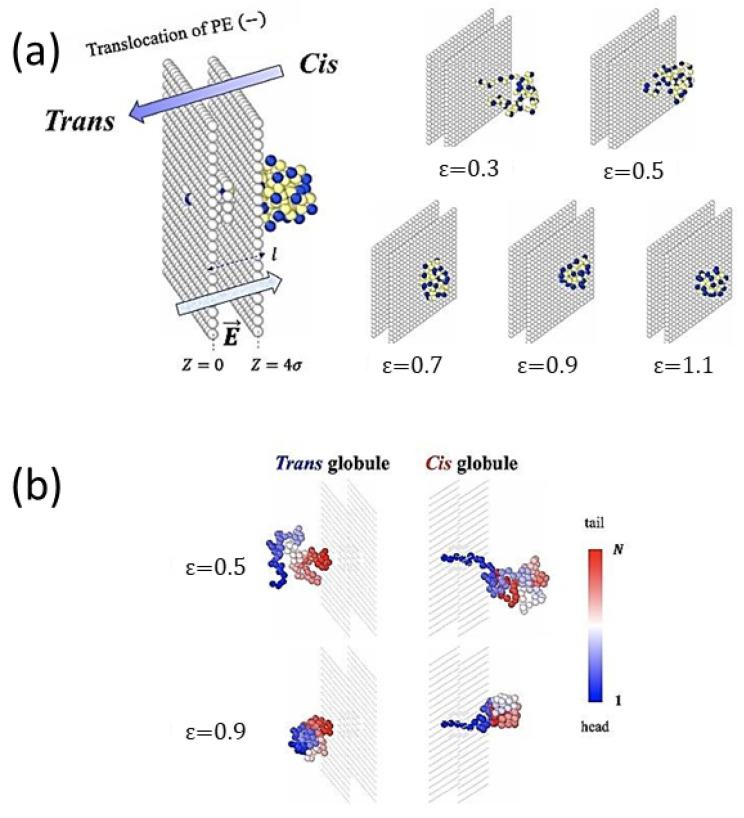
Illustration of a translocation process through a bilayer membrane (5σ wall thickness, where σ is the bead diameter), separating the cis-side from the trans-side of the membrane. (**a**) The negatively charged polymer is driven by the applied electric field from the cis- to the trans-region. Dark-colored beads represent negatively charged monomers and are considered as hydrophilic (polar), while the white ones carry no charge, representing the hydrophobic ones. Different solvent conditions can be described using different values of the attractive LJ potential strength, ε≥0 (i.e., ε=0 for a good solvent), among hydrophobic sites. (**b**) Examples showing the translocation from the cis to the trans side for a polymer of N=90 monomers and two different LJ potential strengths ε=0.5 and ε=0.9. The colors range from blue (head) to red (tail) of the chains (adapted from [270]).

Polyelectrolyte pore translocation: In [270], the authors study the translocation of a polyelectrolyte chain through a pore driven by an electric field using MD simulations based on a coarse-grained model of hydrophobic-polar (HP) monomers, where the former represent neutral monomers, and the latter charged ones uniformly distributed along the hydrophobic backbone [271] (see Figure 22).

The hydrophobic chain is initially found in the cis-side (Figure 22) in a globular form, where H- and P-type monomers are partially segregated. In order to translocate, the globule must first partially unfold, where interplay between translocation through a realistic pore and globule unraveling is discussed. The translocation dynamics of the chain is studied as a function of the solvent conditions, showing that short translocation times occur for slightly poor solvents (see also [272,273,274,275,276]). The time distribution near the minimum is rather shallow and the translocation time is roughly constant for intermediate hydrophobicities. The chain dynamics depends on the channel- and chain-internal frictions related to globule uncoiling. The results are compared with the predictions of a simplified Fokker–Planck equation describing the head monomer location in space.

**Figure 23:**
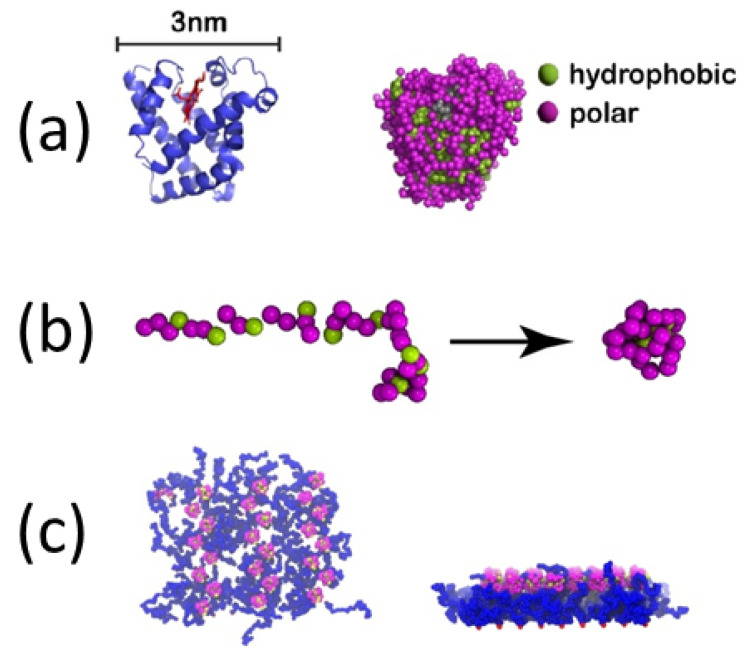
(**a**) Myoglobin protein: (a-left image) Native structure showing the α-helices. (a-right image) Coarse-grained model consisting of 151 amino acids, each one represented by a sphere. Polar amino acids are in red color, while hydrophobic (non-polar) ones are in green color. (**b**) Coarse-grained model of S25 protein consisting of 40 polar amino acids and 25 hydrophobic ones: (b-left image) Starting elongated configuration. (b-right image) Collapsed structure, where the hydrophobic sites have been brought inside the structure, leaving essentially all polar ones on its surface. (**c**) Planar polymer ‘mushroom’ in the case of 24 S25 proteins: (c-left image) Top view perpendicular to the surface. (c-right image) Side view (adapted from [277]).

Protein adsorption dynamics: In [277], the authors study universal aspects of protein adsorption dynamics by polymerized surfaces, using both a coarse-grained model for hydrophobic polar proteins (see Figure 23) and a polymer brush representing the binding surface. The proteins are uniformly placed on top of the brush whose multibead–spring chains are tethered to a solid implicit wall. They find that the most important factor determining the adsorption efficiency is the brush grafting density, while protein size and related hydrophobicity also play a role [278,279,280,281]. The rate of protein adsorption, density profiles and protein shapes, together with the respective mean binding force are compared for the different protein adsorption scenarios.

**Figure 24:**
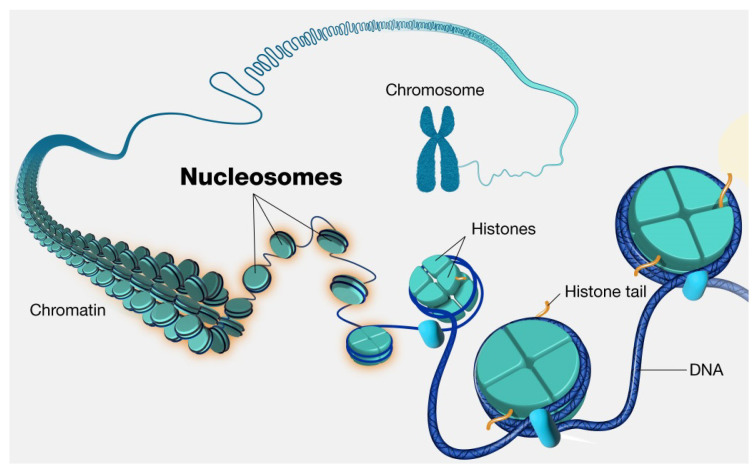
Schematic structure of chromatin showing the nucleosome units, inside which DNA pairs are wrapped around histone proteins. Close chromatin (called heterochromatin) is densely packed (**left** side of the figure) and transcription cannot occur. For transcription to occur, chromatin must be open, yielding the so-called euchromatin (**right** part of the figure). Condensing DNA into chromosomes (**top right** part of the figure) prevents DNA tangling and damage during cell division. Courtesy: National Human Genome Research Institute (https://www.genome.gov/genetics-glossary/Nucleosome (accessed on 27 November 2024)).

Nucleosome dynamics: A nucleosome is the basic repeating subunit of chromatin packaged inside the cell’s nucleus [282,283]. In humans, DNA is typically 1.8 m long and it must be packed into a cell nucleus of about 10 μm diameter, where nucleosomes play a key role in packing DNA. A single nucleosome consists of about 150 base pairs of DNA sequence wrapped around a core of eight histone proteins (see Figure 24). To allow for DNA transcription, chromatin must first be brought into its open form called euchromatin (right part of the figure). In contrast, to yield the compact chromosome structure, the nucleosomes must repeatedly fold onto themselves. However, much less clear is the picture of nucleosome regulatory mechanism, thus requiring a more systemic discussion (see, e.g., [284,285,286]). In [287], the authors review the regulatory factors of nucleosome dynamics by considering histone modification, DNA methylation, nucleosome-transcription factors and nucleosome-remodeling proteins and cations, from both computational and experimental approaches. It is suggested that integrating the latter with suitable nucleosomes packing modes, and the application of deep learning techniques, can yield promising opportunities for further discoveries.

Model polymers vs. globular proteins: Recently, the issue of linear chain molecules has been addressed using MC simulations of standard polymer chains of tethered spheres, at both low and high temperatures, and their behavior compared with experimental data on globular proteins taken from the Protein Data Bank (PDB) [288]. They discuss detailed analyses of both local and non-local structures (see also [289,290,291]), and the associated maps of their closest contacts, as an attempt to reconcile using symmetry considerations the apparently different behaviors observed for the model and real chains.

### 2.8. Polymers in Biology: Biomedical Applications

Biomedical applications of polymers: The search for materials having applications in human body-related issues is a rapidly evolving field in the so-called biomedical sector. Such biomaterials can be fully synthetic or based on natural components fulfilling strict requirements of biocompatibility. Their range of applications is broad, being used for the development of prostheses, replacement of bones or tissues, and even artificial organs. Interestingly, the use of polymers in biomedical applications has significantly increased during the last few decades [292,293,294], despite some intrinsic limitations such as their higher costs than standard materials, lower strength compared to metallic components, lack of advanced design optimization and insufficient incorporation of reinforcement components. In [295], the authors review biomedical applications of polymers related to important advances in the synthesis and modification techniques for obtaining novel biomaterials, e.g., aimed as replacements of both hard and soft tissues, and to be used in limb prostheses, dentistry and bone fracture issues. The emergence of less invasive surgery techniques, together with faster surgical sutures, can contribute to the further expansion of the use of polymers in biomedicine.

Three-dimensional (3D) polymer hydrophilic networks: Among polymer biomaterials, 3D polymer hydrophilic networks, made of crosslinked macromolecular chains known as polymeric hydrogels (Section 2.1 and [90]), have attracted a great deal of attention [296,297,298]. They exhibit high swelling in water and water retention, suitable response to external stimuli, and adaptable mechanical features. In addition to the biomedical sector, polymeric hydrogels have found applications also as hydrogel electrolytes in flexible aqueous energy storage devices. In [299], the authors discuss synthetic processes for developing polymer hydrogels using polymerization techniques, such as frontal polymerization (FP) based on a propagating self-sustained reaction across an ensemble of monomeric units in a batch reactor. A summary is presented of the main recent developments achieved related to the design, preparation, and application of FP-derived polymeric hydrogels.

Skin repair issues: The outermost layer of the human body, called the integumentary system, includes skin, hair, scales, feathers, hooves, and nails, of which the skin constitutes the largest organ. The skin has up to seven layers of ectodermal tissue, roughly described in terms of the superficial epidermis, the subcutaneous hypodermis and the deeper dermis, and it is embodied with a complex self-regulatory function. The epidermis is composed largely of keratinocytes, undergoing constant renewal due to the action of epidermal stem cells that keep producing either new or translocation expansion cells. The skin protects the body against pathogens and excessive water loss, while it is devoted to heal itself in case of damage by forming a scar tissue [300,301,302]. It has recently been shown that it can also absorb man-made per- and polyfluoroalkyl substances (PFAS), known as forever chemicals, which are highly persistent chemicals not present in nature. In [303], the authors deal with the issue of wounds unable to effectively heal via normal repair mechanisms by reviewing different healing strategies such as the possibility of inducing changes in physicochemical and biological properties. The latter are obtained by incorporating different polymers and fillers into specifically designed polyurethane dressings, discussing their applications in wound repair and regeneration. Several polymers are considered, of which natural-based ones include collagen, chitosan, and hyaluronic acid, while synthetic-based polymers are constituted by polyethylene glycol, polyvinyl alcohol, and polyacrylamide, in addition to other active ones such as the antimicrobial trifluoroacetate salt (LL37 peptide), platelet lysate, and exosomes. They conclude with a discussion of future developments and applications of novel polyurethane dressings.

**Figure 25:**
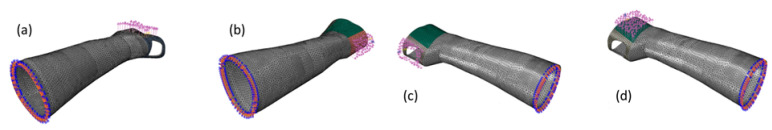
Finite elements (FE) mesh, boundary conditions, and load directions for four crucial wrist movements: (**a**) flexion, (**b**) extension, (**c**) radial and (**d**) ulnar. The Abaqus software (from Dassault Systems [307]) was employed for the FE analysis of orthoses, where the splint exhibited 11,481 node points and 37,225 linear four-node tetrahedral elements (adapted from [307]).

Material models for limb orthoses: FDM is a widely used technique in the biomedical sector (see also Section 2.2 and Section 2.5, and [117,121,209]) due to its versatility in dealing with various materials. However, the appropriate material selection for a specific application can become an issue, and the understanding of the mechanical behavior, in particular of polymeric materials, becomes essential to obtain the desired result [304,305,306]. In [307], using the finite elements numerical approach (see examples in Figure 25), the authors analyze three material models: The Bergström–Boyce (BB), the three-network (TN), and the three-network viscoplastic (TNV) models, in order to determine the mechanical behaviors of the polymeric materials, acrylonitrile butadiene styrene (ABS), polylactic acid (PLA), and polyethylene terephthalate glycol (PETG), aimed to be used in personalized upper limb orthoses. The new approach thus relies on the combination of both theoretical and experimental investigations to predict the suitability of the mechanical characteristics required for obtaining accurate personalized orthoses.

## 3. Theoretical Aspects of Polymers

The simplest polymer chains are those constituted by *N* (monomers) units, each one representing a molecule of the same type. They are typically considered to be immersed in a liquid solution (made of single molecules), which allows the chains to move around, displaying in general a quite challenging dynamical behavior. This complexity arises because of intermolecular interactions, in which the monomers are covalently bonded to their neighboring partners having bond strengths much larger than the other interactions present in the system, thus providing the chain with a good stability even at relatively high temperatures. In addition to these purely local interactions, the molecules exhibit a strong repulsion to other monomers, located beyond their nearest-neighbor ones, if they become close enough in space. In many cases, however, there is a medium-long-range attractive interaction present between such non-local partners which may become important at low temperatures. Typically, they represent van der Waals types of attractions, which may dominate the whole interaction behavior in the case of neutrally charged monomers, leading to a collapse of the chain below some critical temperature, $T_{\theta }$.

### 3.1. Deterministic and Random Fractals: Percolation Clusters and SAWs

**Figure 26:**
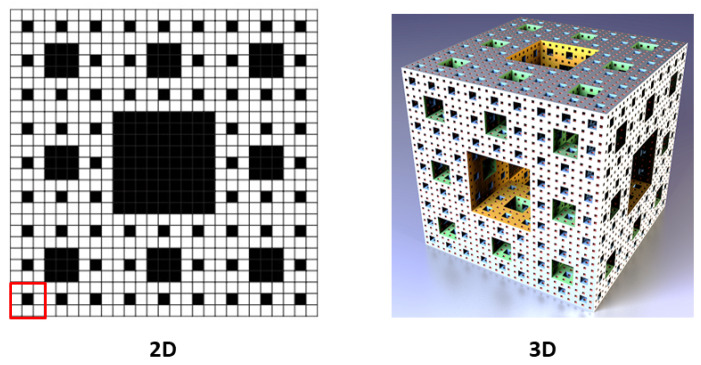
Illustrations of the Sierpinski fractal carpet in 2D and the fractal sponge in 3D [308]. Both fractals are generated according to the rule (n,k), where *n* is the length of the initiator and *k* refers to the number of subunits deleted from the generator (c.f. the red square (‘seed’) shown at the bottom-left part of the figure). The seed is made of n×n (here n=3) squares (cubes), of which the central square (k=1) is deleted for the 2D case, and k=7 cubes are deleted for the 3D fractal. The process is continued deterministically afterwards. The 2D fractal is shown up to the third iteration, and the 3D sponge up to the fourth one. The sites available to the SAW are shown as white squares in 2D and white cube faces in 3D. The fractal dimensions, $d_{f}\left(d\right)$, can be calculated exactly, $d_{f}\left(d\right)=\ln\left(n^{d}-k\right)/\ln\left(n\right)$, yielding $d_{f}\left(2\right)≃1.89278$ and $d_{f}\left(3\right)≃2.72683$.

For temperatures $T\gg T_{\theta }$, highly diluted chains attain an open structure displaying a universal scale-invariant shape whenever the single molecules in solution behave as a ‘good solvent’; i.e., they fully screen non-local interactions between monomers. In these circumstances, the so-called SAWs, which mimic the internal connectivity of a chain, can be used to model diluted polymer chains. Effects of non-local, not fully screened interactions emerge in a poor solvent and can still be treated within the SAWs models at finite temperatures. The simplest case of an SAW in a good solvent is the one in which only hardcore interactions are considered between non-local monomers. However, one can imagine situations in which SAW chains can move either on top of a solid surface or in a solution immersed within a porous medium. The most interesting materials for us are those which display some type of fractal scaling in space, since in this case the standard universality class of the SAW can be modified. Ideal fractal materials can be modeled by relying on specific construction rules yielding mathematically exact fractal shapes. Examples of such deterministic fractal objects are displayed in Figure 26, in which the lack of disorder allows us to concentrate on the effects of the fractal medium alone on the scaling properties of SAWs.

The ideal fractals shown in Figure 26 may still be built experimentally as follows. The 2D carpet could be created by deposition techniques using a fractal ‘mask’, in which the black zones are grown along the vertical direction creating a sort of barriers for the SAWs, preventing them from occupying those locations and thus forcing the chains to wonder over the white available places. The 3D case is a bit more cumbersome, since the building of the porous material would require 3D printing techniques. To study fractal effects, two possibilities can be envisaged regarding which part of the structure represents the polymer conducting medium. To be noted is that both the white cubes and the empty pores constitute connected 3D structures by their own. In the case shown in Figure 26, the polymers can be considered to slide along the white surface areas, thus effectively moving through a space of reduced dimension, $d_{f}<3$. The second possibility would be to consider the complementary set to the one shown in the figure, in which the pores are 3D printed and becoming the solid part of the object. The remaining empty part of the object would build a conducting medium of dimension $d_{f}$ once filled with a good solvent, allowing the polymers to diffuse through it. The connected solid part could eventually be made of a transparent material in order to monitor the polymer(s) motions inside the structure.

**Figure 27:**
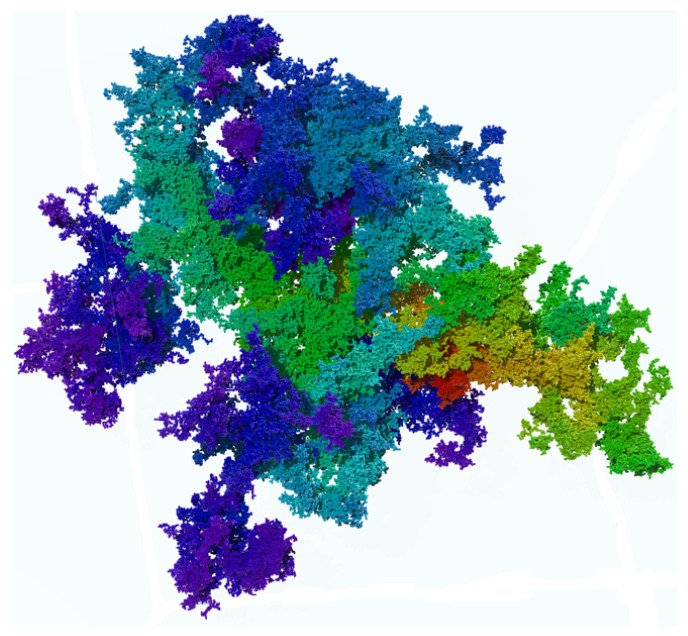
A large percolation cluster at the critical site concentration p≃0.3116 in 3D on a simple cubic lattice. The cluster has been grown using standard techniques [315,316] (see also [310,317] for a generalization of the growth rules). The fractal dimension of percolation clusters is $d_{f}≃2.53\pm 0.02$ (see, e.g., [318]). The different colors represent the length of the shortest (linear) path connecting a site to the seed of the cluster, the latter is located within the red sites and the blue sites have the longest paths (or chemical distances [310]) from the seed. The cluster contains over a million sites, and the longest chemical distance is 2000 in lattice units (courtesy of Francesco Marini). The fractal dimension of the shortest path is $d_{\min}=1.13077\left(2\right)$ in 2D and $d_{\min}=1.3756\left(6\right)$ in 3D [319]. The shortest paths are essential when dealing with the transport of matter along the connected sites of the cluster.

More realistic fractal media, closer to the case of natural shapes [309], can be modeled by different types of random fractals (see, e.g., [309,310,311,312]), among which percolation clusters are very useful, since they describe a quite general type of structural disorder [310,312,313,314] (see Figure 27).

**Figure 28:**
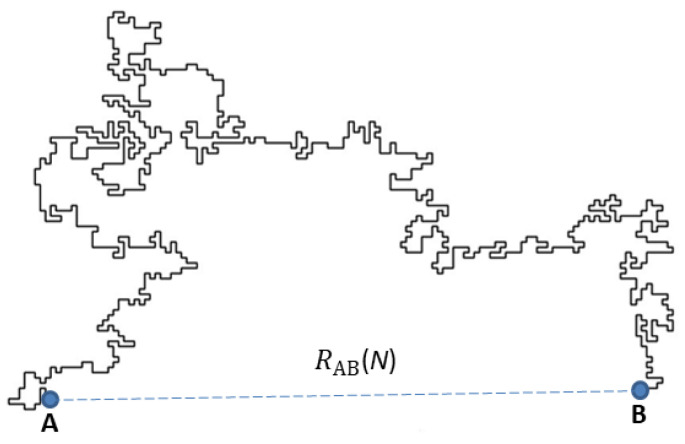
A linear polymer in a good solvent, modelled by a 2D SAW of 1000 monomers on the square lattice, generated using the reptation method discussed in Section 3.2.4. SAWs are linear fractals with a fractal dimension $d_{f}=4/3$ in 2D (see e.g., [324]). Interestingly, the shortest paths in percolation clusters are less compact than SAWs in both 2D and 3D. In the figure, we have highlighted the ends points of the trace of the SAW, denoting them with the letters A and B. The dashed line has a length $R_{\mathrm{AB}}\left(N\right)$, which upon a configurational average depends only on the number of SAW steps, *N*, between the endpoints (here N=1000). Self-trail crossing are forbidden for SAWs.

As is apparent from Figure 27, large percolation clusters exhibit a beautiful complex topology which necessitates more than one fractal dimension to fully describe it, in this case the mass fractal dimension, $d_{f}$, and the one of the shortest paths, $d_{\min}$. The latter brings us to the concept of linear structures displaying self-similarity or fractal scaling. Among them, SAWs play a very important role, since they can be used to study the statistical properties of linear polymers [310,320,321,322,323]. An example of an SAW in 2D is shown in Figure 28.

### 3.2. Structural Properties of SAWs in Regular Lattices and in Fractal Structures

Next, we review the structural properties of SAWs in Section 3.2.1 and their relation to fractal percolation clusters. Then, we consider the cases in which SAWs are embedded in deterministic (Section 3.2.2) and random (Section 3.2.3) fractals as well as the way their structural properties change with respect to their standard behaviors in regular lattices. Finally, in Section 3.2.4, we describe a simple method for generating SAWs of arbitrary length using the reptation method valid in any substrate.

#### 3.2.1. Structural Properties of SAWs in Regular Lattices

SAWs display a characteristic spatial extension as a function of the number of steps (monomers), *N*. To obtain it accurately, one considers an SAW trail for fixed *N*, such as in Figure 28, and perform an average of the end-to-end distance square, $R_{\mathrm{AB}}^{2}\left(N\right)$, over fully distinct configurations. The result can be written in the scaling form,

(1) $$R\equiv {\langle R_{\mathrm{AB}}^{2}\rangle }_{N}^{1/2}≃a\;N^{\nu },\;\mathrm{for}\;\;\;N\gg 1,$$

where the scaling exponent is given by the Flory [320] celebrated relation,

(2) $$\nu \left(d\right)=\frac{3}{d+2},\;\mathrm{with}\;\;\;\nu \left(d\right)=\frac{1}{2}\;\;\;\mathrm{for}\;\;\;d\geq 4.$$

Note that ν(d) depends on the spatial dimension *d*, yielding the exact result for d≠3, except for logarithmic corrections at the critical dimension, $d_{c}=4$, above which they become similar to random walks (RWs). For convenience, one often considers a lattice to generate the SAWs configurations, but the same result for ν(d) holds in the continuum.

Efficient algorithms have been developed over time to generate long SAWs chains, of which pivot strategies have become popular (see, e.g., [325,326,327]), including MC methods [328]. An improved pivot algorithm has been recently introduced, yielding the most accurate estimation of ν in d=3 so far, ν(3)=0.587597(7) [329], which is smaller but still very close to the value ν(3)=0.6 predicted by Flory.

To be noted is that Equation (1) can be inverted to obtain the number of monomers (or mass of the SAW) confined within a distance *R* between the end ones, yielding

(3) $$N\left(R\right)≃c\;R^{d_{f}},\;\mathrm{with}\;\;\;d_{f}=\frac{1}{\nu (d)},$$

where $d_{f}$ is the fractal dimension of the SAWs in a space of dimension *d*. Therefore, one finds $d_{f}\left(2\right)=4/3$ (see Figure 28), $d_{f}\left(3\right)≃1.70184\left(2\right)$ (see [329]), and $d_{f}=2$ for d≥4, corresponding to the fractal dimension of standard RWs. We say that SAWs are self-similar objects with fractal dimension $d_{f}\left(d\right)$.

A relevant quantity for SAWs is the total number of distinct chain configurations of *N* steps, $C_{N}$, which is expected to obey the mixed-scaling behavior,

(4) $$C_{N}≃b\;\mu^{N}\;N^{\gamma -1},$$

where μ is the effective coordination number of the lattice considered, and γ≥1 is the second critical exponent for SAWs which depends solely on the dimensionality *d* of the space, and it is denoted as the enhancement exponent. Indeed, γ=1 for RWs. The scaling relation in Equation (4) can be rewritten in the more convenient form for numerical estimations of the exponents,

(5) $$\frac{\ln\;C_{N}}{N}=\frac{\ln\;b}{N}+\ln\mu +\left(\gamma -1\right)\;\frac{\ln N}{N}.$$

This relation has been used in [330] to estimate values of μ and γ on square lattices using the exact enumeration data of all SAWs walks up to N=50 [331,332,333], yielding μ=2.641±0.005 and γ=1.30±0.05, which is in good accord with the accepted values (see Table 1). These calculations were performed as a validation of the numerical approach based on Equation (5), which has been used for obtaining unknown values of the exponents on random fractal supports (see Section 3.2.2 and Section 3.2.3). The exponent γ is known exactly in 2D, γ=43/32 [334], while recent calculations yield the most accurate value in 3D, γ=1.15695300(95) [335]. It is interesting to note that an approximate relation for γ(d) has been obtained by relying on analytical calculations of diffusion in fractal structures, such as RWs, SAWs and percolation clusters, i.e., d>1, yielding [336],

(6) $$\gamma \left(d\right)=1+\frac{4-d}{6},\;\mathrm{with}\;\;\;\gamma \left(d\right)=1\;\;\;\mathrm{for}\;\;\;d\geq 4,$$

from which one finds γ(2)=4/3 and γ(3)=7/6, which is in very good agreement with the accepted values.

**Figure 29:**
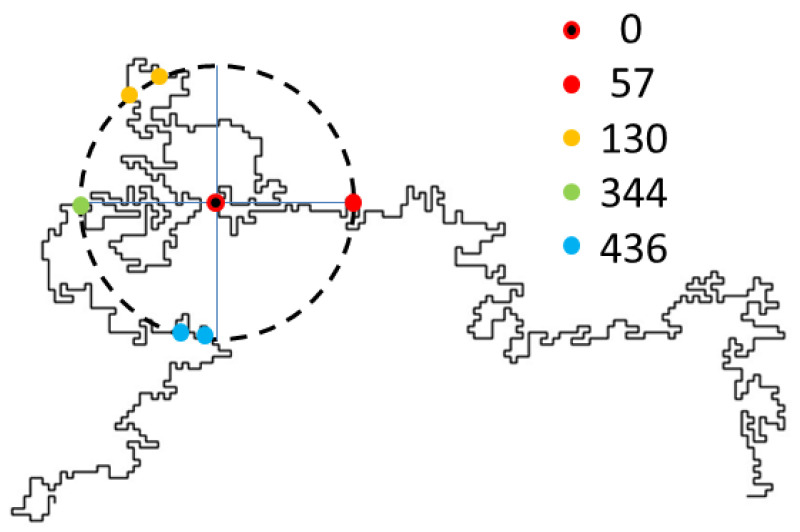
The internal structure of SAWs: The metric *ℓ*, yielding the distance along the chain between two monomers, compared with the Euclidean distance *r* in space between them. In the figure, a site is taken as the origin (black–red circle at r=0), from which one can determine the chemical distances *ℓ* (see the inset for the indicative values of *ℓ*) of those monomers located on the dashed circle at distance *r* from the origin. Then, $\Phi_{\mathrm{SAW}}\left(r,\ell \right)$ is obtained by averaging over all pairs (*r*,*ℓ*).

**Figure 30:**
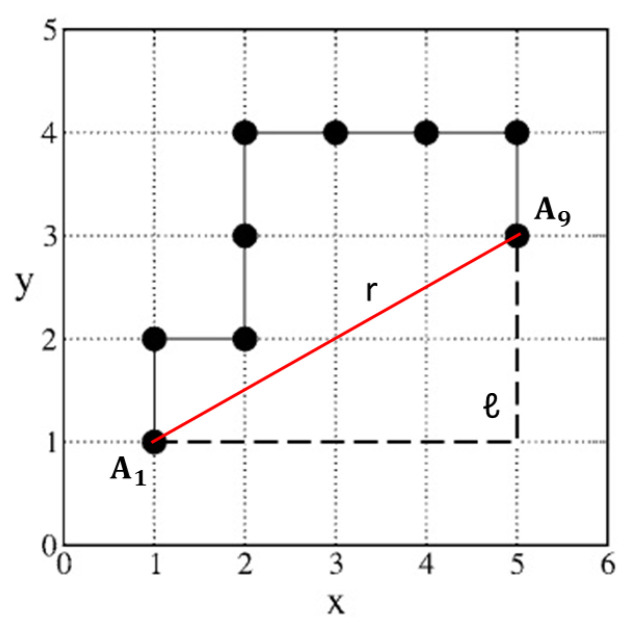
The end-to-end topological distance, ℓ(N) (dashed line), and Euclidean one, r(N) (red line), for an SAW with N=9. Here, ℓ(9)=6 and $r\left(9\right)=\sqrt{20}≃4.472$, in lattice units, which are consistent with the bounds, 4.47≲ℓ≲6.32.

In contrast to its simple one-dimensional topology, an SAW chain attains a rather complex structure in space in the sense that monomers located at the same Euclidean distance *r* from a given chosen site (see Figure 29 for an illustration in 2D) can be found at quite a different number of steps, or topological (chemical) distances *ℓ* [310], from it. The inset in the figure displays the values of *ℓ* for SAW sites at fixed distance *r* from the site chosen as the origin. This feature leads to a rather broad distribution function, $\Phi_{\mathrm{SAW}}\left(r,\ell \right)$, as a function of *ℓ* (see, e.g., Figure I.5 in [324]), which obeys the scaling form,

(7) $$\Phi_{\mathrm{SAW}}\left(r,\ell \right)=\frac{1}{r}\;f\left(x\right),\;\mathrm{with}\;\;\;x=\frac{r}{\ell^{\nu }},$$

where the normalization condition $\int_{0}^{\infty }dr\;\Phi_{\mathrm{SAW}}\left(r,\ell \right)=1$. This scaling form was originally derived for the end-to-end distance, $r=R_{A,B}\left(N\right)$ (Figure 28), where one of the ends, *A* or *B*, is taken as the origin, yielding $\Phi_{\mathrm{SAW}}\left(R_{A,B},\ell_{A,B}=N\right)\equiv \Phi_{\mathrm{SAW}}\left(r,N\right)$ [337]). Here, we use the notation Φ(r,ℓ) whenever we refer to the internal structure of a fractal substrate, while when we consider the statistics of SAWs describing the distribution of the end-to-end Euclidean distance on a given substrate (either regular or fractal one), we refer to it as P(r,N), with $x=r/N^{\nu }$. The distribution can be also considered using the *ℓ*-metric, P(ℓ,N), with $x=\ell /N^{\nu }$, where *ℓ* is the topological distance calculated on the substrate between the end sites. On regular lattices, one has the equivalence, $\Phi_{\mathrm{SAW}}\left(r,\ell =N\right)\equiv P\left(r,N\right)\propto P\left(\ell ,N\right)$ (cf. Figure 30 below).

The scaling function f(x) in Equation (7) displays the asymptotic behaviors,

(8) $$\begin{array}{cc} f(x) & ≃x^{g_{1}+d},\;\;\;\;\;x\ll 1, \end{array}$$

(9) $$\begin{array}{cc} f(x) & ≃x^{g_{2}+d}\;e^{-c\;x^{\delta }},\;x\gg 1, \end{array}$$

where the exponents ($g_{1}$, $g_{2}$, δ) are related to (ν, γ, *d*) according to

(10) $$\begin{array}{cc} g_{1} & =\frac{\gamma -1}{\nu }\;, \end{array}$$

(11) $$\begin{array}{cc} g_{2} & =[d(\nu -1/2)-(\gamma -1)]\;\delta \;, \end{array}$$

(12) $$\begin{array}{cc} \delta & =\frac{1}{1-\nu }\;, \end{array}$$

yielding $g_{1}=g_{2}=0$, and δ=2 for RWs. The expression for $g_{1}$ is due to des Cloizeaux [338], $g_{2}$ was obtained by McKennzie and Moore [339], and δ was first derived by Fisher [337]. A summary of the updated values of the exponents is given in Table 1.

The remarkable accuracy reported for μ(2) in Table 1 [340], was the last station of a long and fascinating numerical journey triggered by an interesting conjecture by Guttmann in the 1980s. Based on the exact result on the exagonal lattice, $\mu_{\mathrm{ex}}=\sqrt{2+\sqrt{2}}$, he suggested that μ(2) might be given by the positive root of $13t^{4}-7t^{2}-581=0$, yielding $\mu \left(2\right)=\sqrt{7+\sqrt{30261}}/\sqrt{26}≃2.63815853034174$. In 2001, Guttmann and Conway [341] obtained the value μ(2)=2.638158534(4), supporting the conjecture. In 2012, Clisby and Jensen [342] confirmed the previous result, obtaining μ(2)=2.63815853035(2). It was only in 2016 [340] that the final word on this issue was pronounced, yielding μ(2)=2.63815853032790(3) (Table 1), thus ruling out the conjecture. On the cubic lattice, the most accurate value obtained so far, μ(3)=4.6840401(50), was reported in [343].

To numerically determine the distribution function, P(r,N), one considers the case ℓ=N in Equation (7) and performs a configurational average of SAWs on the lattice considered. It is, however, more convenient to work with the end-to-end topological distance, $\ell \left(N\right)=|x_{1}-x_{N}\left|+\right|y_{1}-y_{N}|$, instead of $r\left(N\right)=\sqrt{{\left(x_{1}-x_{N}\right)}^{2}+{\left(y_{1}-y_{N}\right)}^{2}}$, here written for the 2D case, thus eliminating the fluctuations arising from the conversion of the real value *r* to an integer, $i_{r}=\mathrm{int}\left(r\pm \Delta r\right)$, required to obtain the histogram $H(i_{r},N)$ from which the distribution function is derived. This change of metric is possible because on regular lattices, and also on Sierpinski carpets and sponges, both the Euclidean and the chemical distances on the substrate scale the same, in the sense that ℓ≃r. An example of an SAW for N=9 is shown in Figure 30, on the square lattice. It is easy to convince oneself that $r\leq \ell \leq \sqrt{d}r$, where d=2 in this case, suggesting that indeed ℓ≃r.

**Figure 31:**
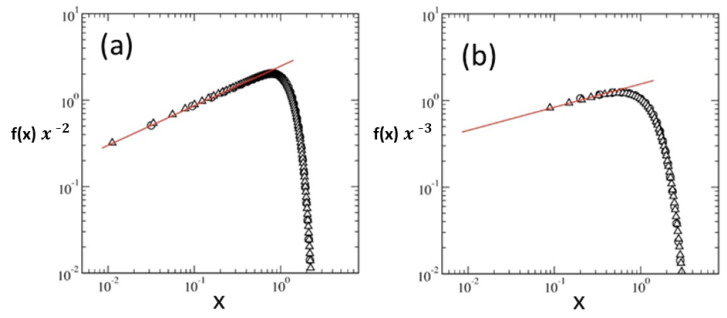
The scaling function for the end-to-end chemical distance (see Equations (8) and (9)), $f\left(x\right)/x^{d}$ vs. $x=\ell /N^{\nu }$, for SAWs on regular lattices: (**a**) square lattice and (**b**) simple cubic lattice, which were obtained using the reptation method. Here, we have used ν=3/4 in 2D and ν=0.5876 in 3D. The circles correspond to the case N=100 steps, and the triangles to N=400 steps. The red straight lines have slopes, $g_{1}\left(2\right)=0.458\left(1\right)$ and $g_{1}\left(3\right)=0.268\left(1\right)$, which are consistent with the results, 11/24≃0.45833 and 0.26711(2), respectively, as reported in Table 1 (adapted from [344]).

The knowledge of exact values of exponents for SAWs is very important for testing numerical approaches aimed at studying cases in which the exponents are not known exactly, as in the fractal structures to be discussed in Section 3.2.2 and Section 3.2.3. Indeed, the reptation algorithm (see Section 3.2.4) turns out to be very accurate to deal with long linear polymers in complex environments. A test case is illustrated in Figure 31, where we have determined the structural function, P(ℓ,N) (the equivalent to P(r,N) in *ℓ*-space), on square and simple cubic lattices, using the reptation method. The results agree very well with the accepted values reported in Table 1.

#### 3.2.2. SAWs in Deterministic Fractals

In order to understand the way in which the scaling behaviors of SAWs are modified if they are embedded in self-similar structures, it is convenient to consider first the case of deterministic fractals, where ℓ≃r holds due to the lack of any source of randomness in the substrate. In fractals in general, the polymer structural functions for the Euclidean and chemical end-to-end distances, P(r,N) and P(ℓ,N), respectively, obey a similar scaling form as in regular systems, Equations (7)–(9), with appropriate values of the exponents (ν,γ), and with *d* replaced by the fractal dimension, $d_{f}$, of the embedding fractal medium. However, the present knowledge on the associated exponents is limited, and further theoretical work is needed to attain a more accurate picture.

Deterministic fractals can be classified into two main categories, finitely and infinitely ramified structures (see, e.g., [345,346]). Finite and infinite ramifications refer to the number of cut operations required to fully disconnect any given subset of the structure. The well-known Sierpinski triangle in 2D, and its 3D counterpart the Sierpinski pyramid (known also as Sierpinski gaskets), are examples of finitely ramified objects, for which renormalization group (RG) techniques can be applied to determine the critical exponents (see, e.g., [347]). In contrast, for infinitely ramified structures, such as those shown in Figure 26, the number of cuts required grows with the length scale. As a result, RG methods cannot be applied and the statistical properties of SAWs must be obtained numerically.

Regarding the exponents, the des Cloizeaux relation [338], Equation (10), seems to hold for finitely ramified fractals, in addition to the expression for δ, Equation (12), which seems to remain valid in general. However, analytical expressions for $g_{2}$, Equation (11), are not known. For infinitely ramified fractals, the des Cloizeaux relation breaks down, since it is essentially based on an RG type of approach. Results for the Sierpinski carpet and Sierpinski sponge (Figure 26) are summarized in Table 2, indicating that the values $g_{1}\neq \left(\gamma -1\right)/\nu$. Also, notice that γ increases with the dimensionality of the embedding space.

A similar behavior for γ has been found for the triangular Sierpinski fractal [349], yielding γ(2)=1.36(3) and γ(3)=1.42(4), suggesting the lack of an upper critical dimension in contrast to the behavior on regular systems. This result is consistent with another family of Sierpinski gaskets in 3D [350], with $d_{f}=\ln\left[\left(b+2\right)\left(b+1\right)b/6\right]/\ln b$, that can be varied in the range $2\leq d_{f}<3$ when the parameter *b* is varied in the interval 2≤b<∞. For b=2 ($d_{f}=2$), they find γ=1.446(17), consistent with the above result, γ(3)=1.42(4) [349], and also that γ(b) increases with *b* (see their Figure 3).

As suggested in Figure 26, the problem of polymers deposition on a rough surface could be a possible application of the present model of SAWs on a Sierpinski carpet. One can therefore consider a heterogeneous surface in terms of an energy landscape, on which the monomer units have different interaction energies, $\epsilon_{i}$, on different locations depending on the type of the local substrate element *i*. For instance, in the case of the Sierpinski carpet, one can prepare a system in which the available fractal sites have an interaction energy, say $\epsilon_{f}$, while the remaining non-fractal sites possess a higher interaction energy, $\epsilon_{b}>\epsilon_{f}$, thus favoring the occupation of the carpet sites by the linear polymer. Indeed, at very low temperatures, $k_{B}T\ll \epsilon_{b}$, one expects the polymer to be fully located on the fractal sites. In contrast, at very high temperatures, $k_{B}T\gg \epsilon_{b}$, the chain would behave as on a regular (flat) surface. In this way, one could study the temperature dependence of the polymer structure function, say, P(ℓ,N)∝P(r,N), and the way the critical exponents can be modified accordingly. An attempt to answer this question has been suggested in [351].

#### 3.2.3. SAWs in Fractal Percolation Clusters

The behavior of SAWs immersed within a conducting network modeled by percolation clusters, ‘sufficiently’ close to the critical percolation threshold, is very different from the one discussed above for regular lattices and deterministic fractals. The essential difference relies on the existence of two distinct spatial metrics within the random structure, which can be quantified by the relation between the length of the shortest path, *ℓ*, and the Euclidean distance, *r*, between two points on the fractals, i.e.,

(13) $$\ell ≃r^{d_{\min}},$$

where $d_{\min}$ is the fractal dimension of the shortest path (see Figure 27). The shortest paths are scale-invariant and look similar to SAWs trails (see Figure 28). The relation (13) holds for all length scales exactly at the percolation critical concentration, $p_{c}$, and it crosses over to a linear regime, ℓ≃r, above the correlation length (see, e.g., [310,312,313]). In the following, we assume we are dealing with percolation systems in which the fractal correlation length is much larger than the system sizes considered. Therefore, all the quantities discussed are described by the scaling behavior expected at criticality.

There is a second important issue regarding the structure of percolation clusters, which is the presence of singly connected portions of the cluster, like a peninsula connected by a narrow bridge to the main land. Such configurations act as traps for an SAW because the chain becomes stuck if it enters them. Therefore, when discussing percolation, we need to exclude those cluster dangling ends and confine our treatment to the cluster substructure which is able to sustain an ‘infinitely’ long SAW.

The substructure of a percolation cluster we are talking about is the backbone of the cluster. It is suitably defined as the subset of cluster sites carrying an electric current when a voltage difference is applied between two sites of the cluster. The so-defined backbone depends on the pair of sites selected for the input and output of the current; however, its statistical properties are robust and well defined [352]. As explained above, it is convenient to study the statistical properties of the backbone in chemical space, since its Euclidean distance counterparts can be obtained via the value of $d_{\min}$. For instance, the fractal dimension of the backbone in *ℓ*-space, $d_{\ell }^{B}$, determines the total number of backbone sites, $N_{B}\left(\ell \right)$, within a distance *ℓ* such that

(14) $$N_{B}\left(\ell \right)≃\ell^{d_{\ell }^{B}},$$

yielding $d_{f}^{B}=d_{\min}\;d_{\ell }^{B}$ in Euclidean space. The structural function, $\Phi_{B}\left(r,\ell \right)$, giving the probability that two backbone sites, connected by a shortest path of length *ℓ*, are at spatial distance *r* from each other, obeys a scaling form similar to those in Equations (7)–(9), i.e.,

(15) $$\Phi_{B}\left(r,\ell \right)=\frac{1}{r}\;f_{B}\left(x\right),\;\mathrm{with}\;\;\;x=\frac{r}{\ell^{\tilde{\nu }}},$$

with $\tilde{\nu }=1/d_{\min}$, yielding $\delta =1/(1-\tilde{\nu })$, and the *g*-exponents are now denoted as $g_{1,2}^{B}$, but no analytical results are known. A summary of the backbone exponents are reported in Table 3.

Several studies of SAW in percolation clusters, both on all clusters and on the backbone of the incipient infinite cluster, have been performed for site percolation at criticality (see [353] and refs. therein). In [353], the authors study SAWs in 2D percolation clusters near the critical concentration, $p_{c}≃0.592745\left(5\right)$. On the backbone, they find for the end-to-end distance exponent ν the value, $\nu_{r}^{B}≃0.77\left(1\right)$, using the Euclidean metric *r*, the enhancement exponent $\gamma_{B}≃1.31\left(3\right)$, close to γ=1.34375 valid on regular 2D lattices, and an effective coordination number $\mu_{B}≃1.46\left(3\right)$, which is barely consistent with the suggested value $\mu_{B}=p_{c}\;\mu_{\mathrm{reg}}≃1.5637$ (see Table 1).

In [354], the mean distribution function of the end-to-end distance of SAWs in *ℓ*-space, P(ℓ,N), was studied on percolation clusters at criticality, which obeys the scaling form

(16) $$\begin{array}{cc} P(\ell ,N) & =\frac{1}{\ell }\;f\left(x\right),\;\;\;\;\;\;\;\;\mathrm{with}\;\;\;x=\frac{\ell }{N^{\nu_{\ell }}}, \end{array}$$

(17) $$\begin{array}{cc} f(x) & =x^{g_{1}^{\ell }+d_{\ell }^{B}},\;\;\;\;\;\;\;\;\mathrm{for}\;\;\;x\ll 1, \end{array}$$

(18) $$\begin{array}{cc} f(x) & =x^{g_{2}^{\ell }+d_{\ell }^{B}}\;e^{-cx^{\delta_{\ell }}},\;\;\;\mathrm{for}\;\;\;x\gg 1, \end{array}$$

where $\delta_{\ell }=1/\left(1-\nu_{\ell }\right)$. The scaling yields the mean value $\bar{\ell }\left(N\right)≃N^{\nu_{\ell }}$, ensuring the normalization condition $\int_{\ell_{\min}}^{\infty }d\ell \;\;P\left(\ell ,N\right)=1$. The function in *r*-space, P(r,N), where now $x=r/N^{\nu_{r}}$ and $\bar{r}\left(N\right)≃N^{\nu_{r}}$, obeys the same scaling behavior as P(ℓ,N), with the corresponding *r*-exponents. MC simulations were performed for site percolation on the square lattice at $p_{c}$ [354] to obtain P(ℓ,N) and P(r,N). Both distributions are related to each other by the convolution

(19) $$P\left(r,N\right)=\int_{\ell_{\min}}^{\infty }d\ell \;\;\Phi_{\mathrm{iIC}}\left(r,\ell \right)\;P\left(\ell ,N\right),$$

where $\Phi_{\mathrm{iIC}}\left(r,\ell \right)$ is the equivalent of $\Phi_{B}\left(r,\ell \right)$ for the whole incipient infinite cluster (iIC) (see [354] for more details). From Equation (19), one can obtain exact relations between the set of exponents in both metrics. The idea is that working in *ℓ*-space yields more accurate values of the critical exponents, from which one can obtain the results in *r*-space according to $d_{r}^{B}=d_{\ell }^{B}\;d_{\min}$, $\nu_{r}=\nu_{\ell }/d_{\min}$, $\delta_{r}=1/\left(1-\nu_{r}\right)$, and $g_{1}^{r}=g_{1}^{\ell }\;d_{\min}$, but $g_{2}^{r}\neq g_{2}^{\ell }\;d_{\min}$ [354].

It was soon realized that the very existence of structural disorder and their scale-invariant fluctuations yield a multifractal behavior for the enhancement exponent γ and the effective coordination number μ [330]. In other words, the expression in Equation (4) must be generalized to account for the presence of fluctuations, as a function of *N*, arising from different backbone configurations. To investigate the contributions from the latter, one introduces a tune parameter, denoted as *q* in the multifractal literature, where −∞<q<∞, with the help of which one can select different configurations. In the limit q→−∞, one looks at very rare, almost linear backbones, while in the opposite limit, i.e., for q→∞, one can select the most compact ones. The multifractal equation describing the total number of SAW configurations can be written as [330],

(20) $${\langle C_{N}^{q}\rangle }_{B}^{1/q}=A_{q}\;\mu_{q}^{N}\;N^{\gamma_{q}-1},$$

where ${\langle \ldots \rangle }_{B}$ represents averages over different backbone configurations, $\mu_{q}$ are generalized effective coordination numbers and $\gamma_{q}$ generalized enhancement exponents. By varying *q* in (20), one can extract the values $\left(\mu_{q},\gamma_{q}\right)$ associated with each different type of backbone structure.

In order to proceed, we write Equation (20) in a form similar to the one considered in Equation (5) for regular systems,

(21) $$\frac{1}{N}\ln\left({\langle C_{N}^{q}\rangle }_{B}^{1/q}\right)=\frac{\ln A_{q}}{N}+\ln\mu_{q}+\frac{\gamma_{q}-1}{N}\ln N.$$

Among all the values of *q*, just two of them play the most relevant roles. They are the q=1 and q=0 moments of $C_{N}$. The former yields the so-called annealed average,

(22) $${\langle C_{N}\rangle }_{B}=A_{1}\;\mu_{1}^{N}\;N^{\gamma_{1}-1},$$

which is obtained using exact enumeration (EE) calculations [330], and the second one, the quenched average,

(23) $$\frac{1}{N}{\langle \ln C_{N}\rangle }_{B}=\frac{\ln A_{0}}{N}+\ln\mu_{0}+\frac{\gamma_{0}-1}{N}\ln N,$$

yielding ‘typical’ mean values accessible in MC calculations. Therefore, the discrepancy found in previous works between EE and MC calculations can be resolved within this multifractal scheme, suggesting that the case q=1 corresponds to the EE results, while q=0 to the MC ones. Indeed, it is found that the relation $\mu_{1}=p_{c}\;\mu$, where μ is the effective coordination number in the regular lattice, holds very well in both 2D and 3D [330]. A summary of the exponents describing SAWs on the backbone of critical percolation clusters in 2D and 3D is reported in Table 4.

The problem with $g_{1}^{r}$ in percolation clusters: The results for $g_{1}^{r}$ presented in Table 4 are rather inconsistent (even in 2D) with the des Cloizeaux relation, g=(γ−1)/ν, obtained for SAWs on regular systems. Nor does it work in the case of infinitely ramified deterministic fractals (see Section 3.2.2). So, the question arises regarding whether we may gain some new insight yielding a better theoretical estimate for $g_{1}^{r}$ in the case of critical percolation clusters. A step along this direction has been attempted in [355], yielding the new relation,

(24) $$g_{1}^{r}=\frac{\gamma_{1}-1}{\nu_{r}}+\frac{\beta }{\nu }\;,$$

where β describes the probability that an arbitrary site belongs to the incipient infinite cluster, standardly denoted as $P_{\infty }$, which for concentrations $p≳p_{c}$ behaves as

(25) $$P_{\infty }≃{\left(p-p_{c}\right)}^{\beta },\;\mathrm{for}\;0<\left(p-p_{c}\right)/p_{c}\ll 1,$$

and ν describes the divergence of the correlation length near $p_{c}$, $\xi ≃|p-p_{c}{|}^{-\nu }$.

The second term in (24) describes the additional difficulty that the *N*th monomer of the SAW encounters to return close to the first one, taken as the origin, due to the topological constraints caused by the random nature of fractal percolation clusters. Indeed, Equation (24) yields $g_{1}^{r}=0.54\left(7\right)$ in 2D, and $g_{1}^{r}=0.92\left(8\right)$ in 3D, which is in very good agreement with the numerical results in Table 4. To be noted is that above the critical dimension $d_{c}=6$, β=1, ν=1/2 and $\gamma_{1}=1$; therefore, the second term in (24) yields the expected result, $g_{1}^{r}=2$ (see [355] for further details). Finally, a review of the SAWs behavior in fractals, including triangular Sierpinski gaskets, can be found in [356].

#### 3.2.4. The Reptation Method

The reptation model [357] aims at describing the dynamics of a whole SAW chain in a melt, thus describing the motion of a single linear polymer in the presence of other similar ones (see also [321,322] and more recent literature [11,12,52,358,359]). Clearly, de Gennes’ idea of an SAW attempting to move among ‘obstacles’ [357] can be easily extended to study SAW diffusion within an either deterministic or disorder fractal.

Here, we extend the method further to deal with static SAW configurations in a lattice. We have already shown that this is indeed possible, yielding very accurate results in the case of regular lattices (see Figure 31). In the following, we discuss the lattice reptation method able to generate suitable SAW configurations in a general domain that is either regular or fractal. We present MC simulations indicating that reptation yields results consistent with the SAW statistical properties in regular lattices (see Section 3.2.1).

**Figure 32:**
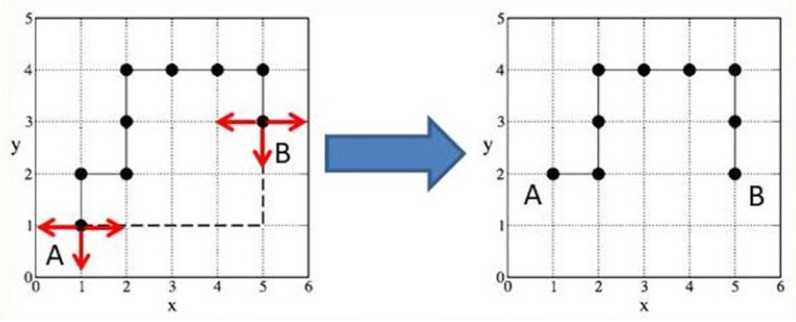
The reptation method on a square lattice. A chain of 9 monomers is shown, where the polymer ends are indicated with the letters A and B. The thick lines between the circles represent the chain links. The left panel illustrates the initial configuration, which can be changed by selecting any of the six possible jumps of the chain starting at any of its ends (red arrows). The right panel depicts the new configuration after end B has moved downward carrying the whole chain with it.

The rules for the lattice reptation method are illustrated in Figure 32 in 2D, where the lattice constant equals the intra-polymer bond length. In this example, a chain of N=9 monomers is considered, and the possible motions of the end monomers, denoted with the letters A and B, are indicated. If the selected new site for the end monomer is free, its motions carries the whole chain with it. Notice that we have taken just three possible jumps which point ‘outside’ the chain. Indeed, a fourth movement, for instance the upward one for A or B are automatically realized when B or A move, respectively. In a standard SAW chain, those movements are forbidden.

To generate an SAW configuration of say, N=100 steps in a square lattice of size L=100, one can start from an initial configuration in which the monomers are just disposed along a straight line. Clearly, there is the issue of the lattice boundary, and one can use PBC to treat cases in which the chain attempts to exit the square domain. However, one soon realizes that the initial configuration can be of arbitrary shape, for instance of an L-shape whose sides are 50 lattice units in length, thus avoiding the problem of touching the boundaries, at least in the first reptation steps. Since the span of an SAW, $R_{N}$, grows as $R_{N}≃N^{3/4}$, the chain will, on average, be of size $R_{100}\approx 32$ lattice units, suggesting that the lattice size L=100 should be sufficiently large to admit SAW configurations which do not touch the boundary.

In general, an acceptable SAW configuration will be one in which the trail of the arbitrary initial configuration has been completely left after a sufficiently large number of reptation steps has been performed. This ensures full independence from the initial configuration as required to obtain an SAW. If one takes this final configuration as the new starting one, one should implement additional reptation steps until the new starting configuration has been exited. In this way, a newly indipendent chain configuration can be obtained and used to perform accurate SAW statistics.

We suggest a much simpler method for generating an SAW configuration, consisting of growing a chain from some suitable site, $s_{0}$, of the lattice, in such a way that at every reptation step, the chain increases its length by one unit inside the lattice (imagine that the whole chain is confined at the chosen site, gradually unfolding into the lattice), and the growth stops when the length *N* has been reached. If the leading end of the reptating chain becomes stuck within its self-generated trail, the chain is moved $m_{\mathrm{back}}$ steps backwards along the trail, like rewinding it back into the site $s_{0}$. This amounts to delete the last $m_{\mathrm{back}}$ monomers of the incipient trail, which are generated again.

**Figure 33:**
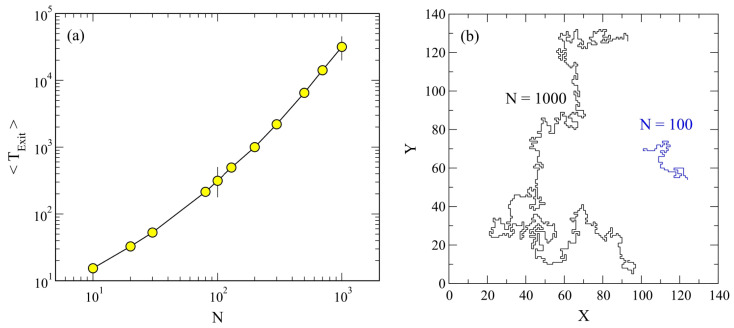
(**a**) Mean exit time steps, $\langle T_{\mathrm{Exit}}\rangle$, to generate a reptation SAW configuration vs. *N*. The continuous line is a guide to the eyes, and the vertical lines are included for illustration, representing a standard deviation. The numerical data can be well fitted with the form, $\bar{T}=0.7\exp\left(1.92\;N^{1/4}\right)$, for N>20. Averages over $10^{4}$ SAW configurations have been performed for the case $T_{\max}=10^{5}$ steps and lattice size L=2000. (**b**) Reptating SAWs on a square lattice illustrated in the cases: N=1000 and N=100 monomers.

In some circumstances, two or more $m_{\mathrm{back}}$ consecutive deletions may be needed. The parameter $m_{\mathrm{back}}$ can be adjusted so that the final chain is created within a maximum number of reptation steps, $T_{\max}$. Here, the total number of steps to fully exit the trail is denoted as the exit time, $T_{\mathrm{Exit}}<T_{\max}$. We have chosen $T_{\max}$ so as to minimize the computing time, avoiding the occurrence of rejected configurations which have not reached the *N* steps within the maximum allowed time. An illustrative example of typical values of the mean exit times, $\langle T_{\mathrm{Exit}}\left(N\right)\rangle$, is reported in Figure 33a as a function of chain length *N*. In Figure 33b, two SAW configurations for N=100 and N=1000 are shown, where one can notice an emerging self-similarity of the chain structures.

In the case of a fractal, two types of averages are required. The first one consists of generating several SAWs starting from a given fractal site, and there is a second one in which different starting locations are considered to explore the self-similarity of the structure. Clearly, for deterministic fractals, one should only consider initial points which are not equivalent by symmetry, while in random structures, any fractal site is a suitable starting point for growing SAWs.

**Figure 34:**
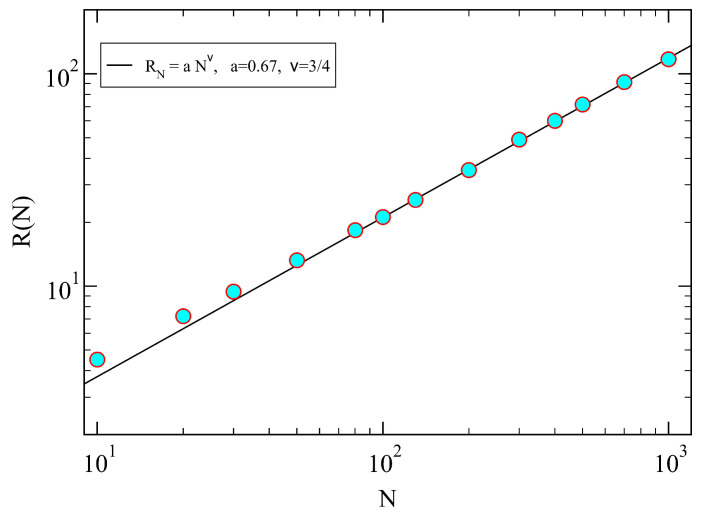
Root mean square of the end-to-end distance, $R\left(N\right)=\sqrt{\langle R^{2}\left(1,N\right)\rangle }$, vs. number of monomers, *N*, on the square lattice (full circles), for chains generated with the reptation method fulfilling the ‘exit’ condition discussed in the text. The straight line has the slope ν=3/4, which is in excellent agreement with the expected exact value. The square lattice has size L=2000, and averages over $10^{4}$ SAW configurations have been perfomed.

The question remains whether these reptating linear structures are truly SAWs. The first test consists in calculating the mean end-to-end distance, R(N), of the chains as a function of *N*. Results for R(N) are shown in Figure 34, suggesting that the reptating chains are consistent with the expected scaling behavior for SAWs with ν=3/4. Similarly good results are found in 3D (in simple cubic lattices), which are discussed in Section 5.2 when modeling the attachment of polyethylene glycol (PEG) chains to a plasma-treated surface.

**Figure 35:**
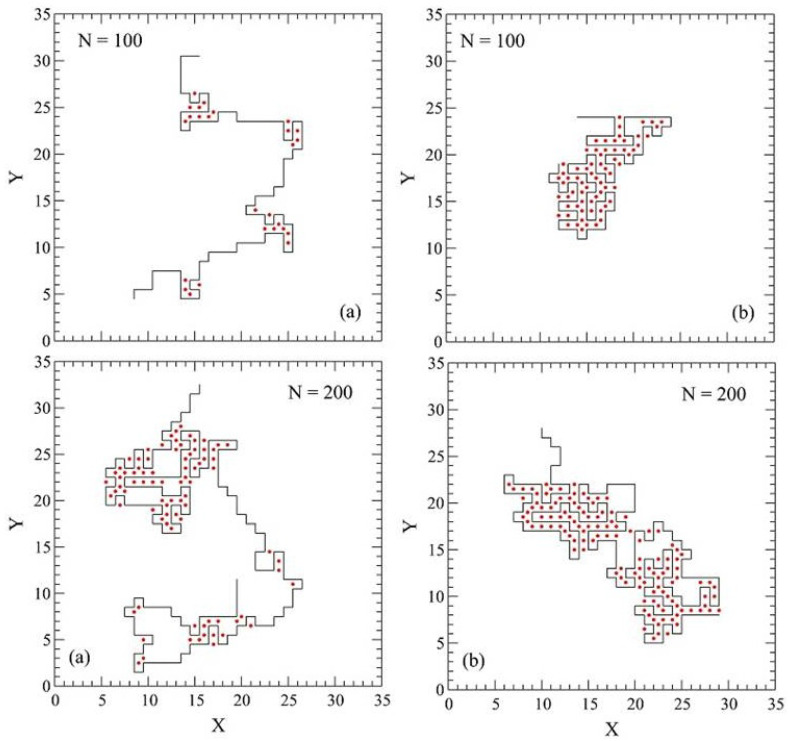
Examples of bridges, *B* (virtual non-local links between two monomers in space indicated by the red dots), for SAWs generated with the reptation method on a square lattice. (**Upper panel**) N=100 in the cases: (Upper a) Typical configuration with a low number B=26 bridges, and (Upper b) compact one with B=67 bridges. (**Lower panel**) Same as in the upper panel in the case N=200 for: (Lower a) Typical configuration with B=83 bridges, and (Lower b) compact one with B=123 bridges. A bridge is a virtual link connecting two monomers (i,j) which are nearest neighbors (one lattice unit distance) in space, but they are separated by at least three links along the SAW chain, i.e., |i−j|≥3.

**Figure 36:**
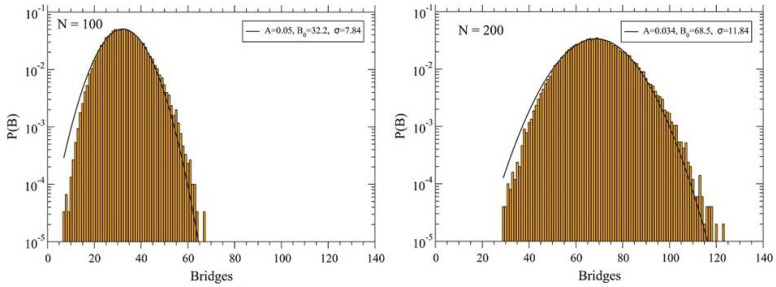
The distribution of the number of bridges, P(B) (bars), vs. number *B*, for reptating SAWs in the cases: N=100 and N=200, obtained by generating 5 $10^{4}$ configurations each. The continuous lines represent Gaussian fits, P(B)=Aexp[−(1/2)((*B* − $B_{0}{)/\sigma )}^{2}]$, yielding (A=0.05, $B_{0}=32.2$, σ=7.84) for N=100, and (A=0.034, $B_{0}=68.5$, σ=11.84) for N=200.

Another interesting quantity to look at is the distribution function of the number of non-local bridges in SAWs. A non-local bridge between monomers *i* and *j* can occur if they are located at a chemical distance ℓ=|i−j|≥3. Illustrative examples of such bridges are shown in Figure 35, in the cases N=100 (upper panel) and N=200 (lower panel). For convenience of visualization, the positions of the non-local ‘contacts’ are indicated by the red dots, which are located at the middle point between sites (i,j), which are one lattice constant apart in space. Knowledge of the total number of non-local contacts for an SAW is important for estimating values of the collapsed temperature, $T_{\theta }$, as we briefly discussed in the Introduction to Section 3 (see also [116]). The distribution function of the number of bridges, P(B), is shown in Figure 36, in the cases N=100 and N=200. Notice the asymmetric shape of P(B) displaying a positive skewness. Calculations of $T_{\theta }$ for reptating chains and the comparison with known results in the literature remain to be performed.

The use of the reptation method is not limited to the problem of constructing single SAW configurations, but it can be extended straightforwardly to the realm of a many-chain ensemble. To generate an initial many-polymer configuration, one can grow reptating chains sequentially, in which the previously created chains remain fixed at their locations. In this case, the newly generated chains experience an ever-increasing difficulty to find available sites for growth, meaning that the chains are treated differently. In some cases, however, it might be necessary to avoid this undesired growth artifact explicitly. To this end, one can also generate reptating chains from single sites, the latter distributed at random within the system. Then, one starts growing chains one after the other, but one monomer at a time. If a chain becomes stuck, monomer deletions are applied to all chains to treat them in a consistent fashion, without giving preference to any particular chain, and also to keep the algorithm as simple as possible. There is, however, a much simpler solution to this problem if one lets the chains move around when dealing with a many-chain problem confined within a finite system, such as a cell (see Section 6.2).

### 3.3. SAWs in Biology

We discuss the application of SAWs in biology with particular focus on DNA molecules (Section 3.3.1), protein structure and folding (Section 3.3.2), and protein sequence evolution (Section 3.3.3).

#### 3.3.1. The Case of DNA

**Figure 37:**
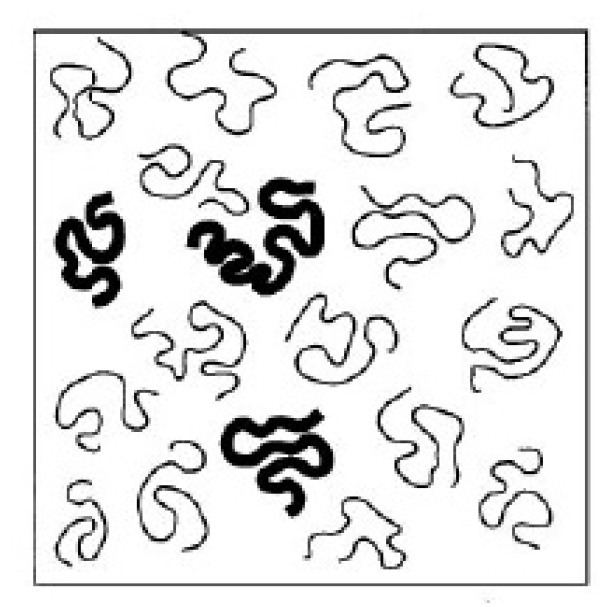
A cartoon of the type of collapsed DNA chain observed in the experiments. The thick lines represent fluorescently labeled DNA molecules, while the thin lines the remaining ones in the sample studied. The former are the actually observed DNA chains (adapted from [360]).

Experimental studies of the behavior of linear polymers on surfaces is essential to test, and eventually validate, the SAW models discussed in the previous sections. For our present purposes, we refer to an experiment in which DNA molecules are kept in contact with a fluid lipid membrane by electrostatic interactions, still allowing the long chains to diffuse on the membrane surface [360]. The work complements observations of reptation dynamics of actin filaments in solution [361]. In [360], the authors were able to vary the concentration of the deposited λ phage DNA molecules by covering a quite broad range of surface densities from a very diluted regime to one characterized by full coverage. Both the structure of DNA chains, as well as their diffusive dynamics, were observed using fluorescence microscopy. For low and medium concentrations, they find that the mean spatial extension of the chains (radius of gyration) obeys ${\bar{R}}_{G}={\langle R_{\mathrm{DNA}}^{2}\left(N\right)\rangle }^{1/2}≃N^{\nu }$, as a function of the number of base pairs *N*, with ν=0.79±0.4, which is consistent with the theoretical value, $\nu_{\mathrm{SAW}}=3/4$ in 2D. They were also able to determine the structure factor in the low–medium concentration regime, yielding the expected fractal scaling, $S\left(k\right)≃k^{-1/\nu }$. Remarkably, at high concentrations, the collapse of DNA chains was observed for the first time (cf. Figure 37), in which the molecules segregate and achieve a much smaller size, ${\bar{R}}_{G}$, than in the low-concentration SAW regime.

When looking at the distribution of collapsed DNA chains shown in Figure 37, one may wonder how the ensemble of linear polymers would behave if they were confined inside a finite spherical domain of say, radius $R_{S}<{\bar{R}}_{G}$. Actually, this question is relevant to the issue of packing DNA long chains into the chromosomes located inside a cell nucleus. An attempt to shed some light into this quest was put forward in [358], based on MD simulations of chains with N=1000 monomers each, confined within a spherical cavity of radius $R_{S}<{\bar{R}}_{G}≃a\;N^{\nu_{\mathrm{SAW}}}$, where *a* is the monomer size and $\nu_{\mathrm{SAW}}≃3/5$ (Equation (2)). It is found that that the rearrangement of the chain configurations does not cost additional free energy with respect to the case of non-confined chains. From these confinement studies, it is then concluded that within a cell nucleus, it is therefore very likely that a global reorganization of eukaryotic chromosomes can be readily achieved during most of the cell cycle. More importantly, it is then suggested that within a spherical boundary, eukaryotic chromosomes may behave themselves as they would in a semidilute polymer solution.

An extension of the simple SAW model presented here is considered in a recent review by Baschnagel et al. [359], in which the authors discuss numerical and analytical results on semiflexible chains (SFC) focused on static properties. SFCs are more involved than simple SAWs in the sense that they possess a local persistent length in which the monomers keep a preferred type of configuration characterized by a stiffness parameter, the latter responsible for the presence of correlations typically decaying with increasing relative chemical distance between monomers. SFC models admit theoretical approaches of a mean-field type, making them very attractive for analytical manipulations. A great deal of attention is dedicated to the issue of polymer interactions with surfaces, which is relevant to many experiments. Indeed, different variants of the model are discussed in detail to cope with the broad range of applications such as polymers confined in thin layers and within strict two-dimensional (2D) layers.

#### 3.3.2. Protein Structure and Folding Dynamics

Proteins are highly complex molecules that are essential for life. They are directly involved in many chemical processes, in addition of being of nutritional value for all living organisms. Proteins of one species differ from those of another species, which make them species-specific. Within a single species, they differ according to which organ they belong to, thus being also organ-specific.

**Figure 38:**
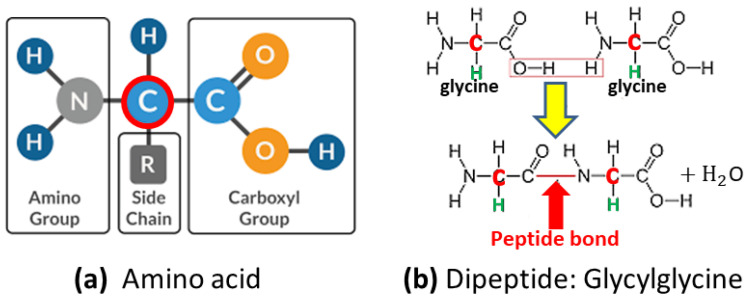
(**a**) The chemical structure of an amino acid. It consists of a central carbon atom (red circle), denoted as $C_{\alpha }$, to which a hydrogen atom and a small molecule, or side-chain residue, are attached. The two remaining $C_{\alpha }$ bonds are occupied by an amino group and a carboxyl one. Amino acids therefore just differ from each other by the type of side chain considered. (**b**) The formation of a dipeptide: the case of glycylglycine. A peptide bond arises when the O-H subgroup of one amino acid (in this case glycine) joins one H unit of the amino group of the second amino acid (the second glycine here), yielding a water molecule. The newly created C-N link is the peptide bond (red line). Note that there are (N−1) peptide bonds within a polypeptide chain of *N* amino acids. Notice that in the case of 20 different amino acids, the size of the sequence space would be $N_{s}\left(N\right)=20^{N}≃10^{1.30N}$, yielding $N_{s}\left(100\right)≃10^{130}$.

A protein is a very large molecule consisting of a series of amino acids which are joined together via the so-called peptide bonds, yielding a long chain of connected units at the very moment of its formation in the cytoplasma of a cell. Although there are more than 500 known naturally occurring amino acids, only 22 play a role in the constitution of proteins and are thus encoded in the DNA. Amino acids are organic compounds and all possess the same structure, as illustrated in Figure 38a. An amino acid is built upon the so-called α-carbon, $C_{\alpha }$, connected to an H atom, a side-chain residue, and amino and carboxyl groups. Two amino acids can be joined together, forming a dipeptide (see the example in Figure 38b in the case of glycylglycine (GG)).

Peptide bonds require about 18 kcal/mol to be formed. This energy is provided by adenosine triphosphate (ATP) hydrolysis within the cell. They are quite strong bonds remaining unaffected up to very high temperatures (above 1000 °K). However, they can be broken by hydrolysis in a water environment, releasing about 3 kcal/mol. Remarkably, the process is extremely slow with a half-life of several hundred years. In the cell, peptide bond breaking is obtained efficiently under the presence of enzymes, known as peptidases or proteases, which accelerate the transition when required.

Once the polypeptide chain has been released in the cytoplasma from a ribosome, it undergoes a spontaneous process of structural collapse, or folding, yielding a unique 3D structure of the protein in its so-called native state. The latter is very specific to the sequence of amino acids present along the polypeptide chain, and it is characterized by a suitable thermal stability within a relatively wide range of temperatures. Once formed, the protein is ready to enter the life cycle of the host body.

The native state has a rather small size compared to the original long structure of the underlying polypeptide, which becomes the internal backbone of the protein. The issue here is that side chains interact among themselves in 3D space, often via H-bonds or van der Waals links, whose interaction strengths are much weaker than that of peptide bonds, the latter being responsible for the backbone stability. These off-chain weak links remind us of the bridges between monomers discussed in Figure 35 for SAWs. The point is that such local pairwise interactions depend on the type of amino acids in ‘contact’ yielding a very complex and typically rough energy landscape, thus making the native state configuration extremely difficult to predict. The final proof that there might exist a one-to-one correspondance between the sequence of amino acids and its ground-state configuration remains so far elusive. This is known as the protein-folding problem. Recently, however, very promising results have been achieved based on the use of deep learning algorithms yielding very accurate predictions of actual native state conformations [362].

**Figure 39:**
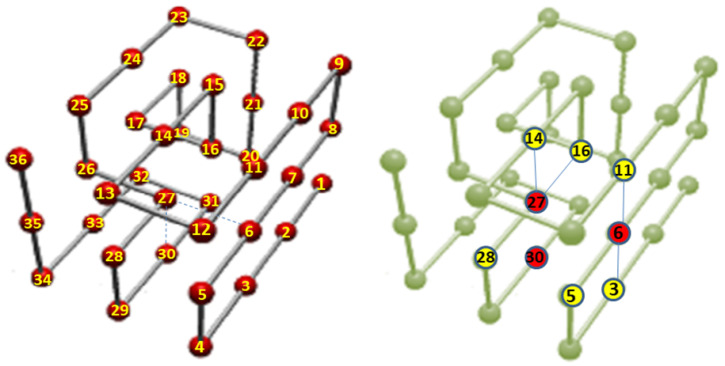
(**Left panel**) A native structure of a lattice protein in a simple cubic lattice having N=36 amino acids (red circles). The structure is chosen to be maximally compact, where the numbers indicate the index of the $C_{\alpha }$ along the chain. This structure has $n_{c}=40$ non-local contacts (or bridges) [363]. The sites (6, 27, 30) (connected by thin dashed lines) represent a sort of ‘hydrophobic core’ of the protein (see text below). (**Right panel**) The red sites (6,27,30) are denoted as hot sites, and the yellow ones (3,5,11,14,16,28) as warm sites [364]. Hot and warm sites happen to be first neighbors in the native structure either along the chain or as non-local contacts (see text below).

One way to qualitatively understand the behavior of polypeptide chains, and as a result shed some light into the mechanism of folding, consists of considering a coarse-grained model of a protein defined in a simple cubic lattice (see, e.g., [363]). A maximally compact coarse-grained native structure of a lattice protein, obtained in the simple cubic lattice, is shown in Figure 39.

In order to mimic the folding dynamics toward a target structure (e.g., the one in Figure 39), which occurs within a suitable range of temperatures, a fitting sequence of amino acids needs to be found. Indeed, if one takes a sequence at random and simulates a chain collapse, it will never reach the target in any finite amount of time. The basic idea is that only ‘special’ sequences of amino acids yielding extremely low values of the total non-local interactions will do the job. This sequence design can be applied very efficiently for any given target structure, since the rare configurations are widely separated from the energy density of states of random sequences, displaying a cospicuous energy gap [363]. The search for optimized sequences can be performed with standard MC simulations by assuming a given typical amino acids composition, in which the non-local contact interactions between, say, 20 amino acid types is taking from a 20 × 20 matrix, $\varepsilon_{n,m}$, with n,m∈(1−20), which has both positive and negative entries, yielding a vanishing value, $\langle \varepsilon_{n,m}\rangle ≃0$, on average. It is indeed found that independently of the initial configuration in space, the chain carrying an optimized sequence, which we denote here as $S_{36}$ in our example of Figure 39, successfully folds into the native configuration, which coincides with its ground state. In this sense, the folding problem admits a rather simple solution in the lattice at least for single domain (globular) types of structures and for not too large numbers of amino acids [363,364].

Once the folding issue of a sequence toward its native structure has been dealt with, the question arises of how stable the structure remains if changes in the amino acids sequence are considered. These changes represent amino acids mutations which are known to occur in natural proteins. Considering this question is also important to understand why a single native structure can admit different amino acids sequences [365].

To this end, we consider the following method for dealing with single amino acid mutations [364]. Let us denote by m(i) the amino acid of type *m* at site *i*. From the sequence of amino acids m(i), i∈(1−N), one can calculate the total internal energy of the ‘wild-type’ chain, $E_{N}\left(\left\{m\left(i\right)\right\}\right)$. To characterize quantitatively the behavior of a mutated chain carrying a single amino acid mutation, $m^{\prime }\left(i\right)\neq m\left(i\right)$, one can calculate the mean local energy variation of the protein, $\Delta E_{m^{\prime },m}\left(i\right)$, as the difference between the energies of the mutated chain, $E_{N}^{\prime }\left(\left\{m^{\prime }\left(i\right)\right\}\right)$, and the wild-type one, averaged over all possible 19 types of different amino acids $m^{\prime }\left(i\right)\neq m\left(i\right)$ at site *i*,

(26) $$\Delta E_{m^{\prime },m}\left(i\right)=\frac{1}{19}\sum_{m^{\prime }=1}^{19}\left[E_{N}^{\prime }\left(\left\{m^{\prime }\left(i\right)\right\}\right)-E_{N}\left(\left\{m\left(i\right)\right\}\right)\right].$$

**Figure 40:**
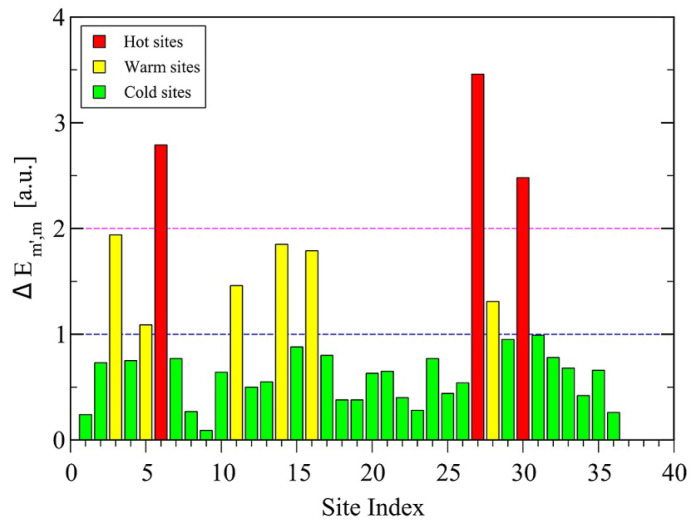
Local energy variations for single mutations at a site *i* in the native structure of $S_{36}$ as a function of site index *i*. The colors indicate sites belonging to the same subset, denoted here as hot sites (6,27,30) (red bars) with $\Delta E_{m^{\prime },m}>2$, warm sites (3,5,11,14,16,28) (yellow bars) with $1<\Delta E_{m^{\prime },m}<2$, and cold sites (1,2,4,7,8,9,10…) (green bars) with $\Delta E_{m^{\prime },m}<1$.

The quantity $\Delta E_{m^{\prime },m}\left(i\right)$ contains essential information about the way the different amino acids become arranged in space within the native configuration. Indeed, there is a great deal of information that one can obtain from this simple approach. An example is reported in Figure 40 in the case of mutations on the optimized sequence, $S_{36}$ [364,366].

The results shown in Figure 40 suggest that one can classify different sites along the chain into roughly three groups: (1) cold sites (green bars in the figure) characterized by mean values $\Delta E_{m^{\prime },m}<1$; (2) warm sites (yellow bars) with $1<\Delta E_{m^{\prime },m}<2$; and (3) hot sites (red bars) where $\Delta E_{m^{\prime },m}>2$. Although these numbers can depend on the actual values of interaction energies employed, a more general picture can be drawn. It is found that for the first set (cold sites), the mutated chain always folds into the target. This is very important, since the cold sites are the majority in the sequence, which explains the fact that a ‘wild-type’ chain may actually admit many different sequences, as observed in natural proteins. In the second group, the mutated chain still folds to a stable configuration, which differs from the target by a small amount, with Q≲1, where *Q* is the fraction of the same contacts present in the target. In some cases, it still folds to the target, yielding Q=1. The third group is critical in the sense that some chains still fold to a state with Q≃1, but many of them just end into a misfolded state with Q≪1, representing a sort of twilight zone for single mutated chains [364]. In addition to these findings, it has been shown that $S_{36}$ also admits more than one mutation at a time [366], which are allowed when the mutated chain energies fall within the energy gap [363] between the wild-type target energy and the onset of the energy distribution for random sequences.

Another interesting problem that can be studied using lattice protein models is the issue of protein aggregation of similar polypeptides [367]. In the presence of a sufficiently high concentration of (denatured or non yet folded) chains, the folding processes do not follow straightforwardly as for isolated chains, but rather, interactions between particular monomers located on different chains can yield to misfolding. The basic mechanism for the occurrence of protein aggregation can be identified by looking at the hot sites of $S_{36}$ (Figure 39), for which the simulations have been performed. A careful examination of the folding path followed by a pair of closely interacting chains provides a clue about what is happening. One can conclude that if one or more hot sites of a protein come in contact with any of the hot sites of the second chain, then the formation of an interchain link takes place and, as a result, none of the chains can fold to the native conformation. They just remain locked in a misfolded state. Remarkably, this is due to that fact that a protein has a (small) number of local partially folded intermediates that are responsible for the control of both protein folding and aggregation. It turns out that for designed lattice proteins, such intermediates are also the elementary structures constituting their folding nucleus [367]. Similarly, heteropolymer interactions within the cell in the form of intrinsically disordered proteins (IDS) display a quite complex behavior (see, e.g., [368,369]).

**Figure 41:**
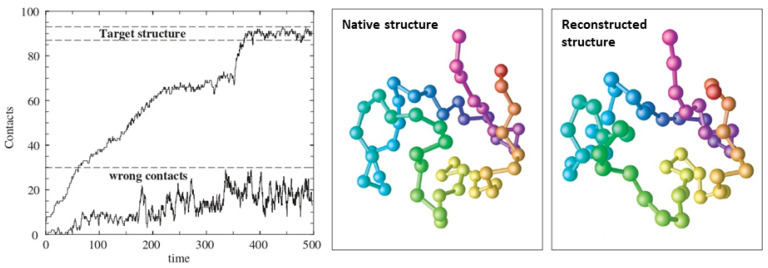
Off-lattice simulation of the reconstruction of Rubredoxin native structure using holonomic constraints for the intrachain bonds. (**Left panel**) The evolution of the number of emerging native contacts as a function of time. For comparison, also the number of new contacts not present in the native structure (denoted as wrong ones) are displayed for illustration. (**Right panel**) Comparison between Rubredoxin native conformation with a reconstructed one. The colors are to facilitate the location of the sites in space (adapted from [370]).

Coarse-grained protein models can be studied in the continuum, in which, in addition to the non-local interactions, one needs to specify how to deal with $C_{\alpha }$–$C_{\alpha }$ interactions along the backbone. In lattice models, this question does not arise because there are no dynamical degrees of freedom associated to such ‘peptide’ bonds; they just keep a constant length all the time. In the continuum, one possibility is to introduce harmonic forces between nearest-neighbor sites along the chain, which must be treated simultaneously with the motion of the chain in space. There is one way to go beyond this traditional approach if one treats the peptide links as rigid without the need for treating any internal dynamics. The price for this simplification consists of rewriting the equations of motion using a Lagrangian approach with holonomic constraints [370]. An illustrative example is reported in Figure 41.

The equations of motion of the chain are solved by assuming a coupling to a thermostat which requires the use of friction coefficients, whose equations of motion are coupled to monomers momenta (for each spatial coordinate) and to the global temperature of the system. No other forces, such as due to the presence of water molecules, are considered in this case [370]. This method allows for reaching the long time-scale dynamics of folding, and of polymer chains in general, which cannot be accessible with traditional, more standard methods. It could also be employed to generate a suitable chain configuration to be used as the starting guess for full-atom MD simulations. The results thus suggest its possible utility in the context of protein structure predictions as well as for dealing with the modeling of polymeric materials at the nanoscale [370].

#### 3.3.3. Protein Evolution in Sequence Space

The lattice protein model discussed in Figure 39 opened the door to the study of the general problem of protein sequence evolution by relying on a solvable model of folding [371]. The basic idea is to search for suitable single amino acids mutations which can be accepted if the resulting new sequence obeys the following three conditions. The first one is that the ground state of the mutated chain must be identical to the reference target structure (conservation of the phenotype). The second one regards its thermodynamic stability, and the third one its kinetic accessibility [371].

These conditions can be monitored in terms of the contact matrix, $C_{ij}$, with (i,j)∈(1−N), defined as $C_{ij}=1$ if a (non-local) contact between sites (i,j) is present in the structure and $C_{ij}=0$ otherwise. To compare two structures, one can rely on the fraction of contacts that both structures have in common (see also the definition of *Q* above). Specifically, the structural overlap is given by $Q(C^{\prime },C^{0})$, where $C^{\prime }$ is the contact matrix of the ground state of the mutated sequence and $C^{0}$ the reference one, defined as,

(27) $$Q\left(C^{\prime },C^{0}\right)=\frac{1}{\max(n_{c}^{\prime },n_{c}^{0})}\sum_{i<j}C_{ij}^{\prime }\;C_{ij}^{0},$$

where ($n_{c}^{\prime },n_{c}^{0}$) are the total number of contacts within each structure, i.e., 0≤Q≤1. Notice that with the help of $C_{ij}$, the configurational energy $E_{N}\left(\left\{m\left(i\right)\right\}\right)$ discussed in Equation (26) can be also written as

(28) $$E_{N,C}\left(\varepsilon \right)=\sum_{i<j}^{N}\varepsilon_{m(i),m(j)}\;C_{i,j}.$$

The first condition amounts to taking $Q\left(C^{\prime },C^{0}\right)=Q\left(C^{0},C^{0}\right)=1$. The second one requires the stability of $C^{\prime }$ along the folding path taking place at a fixed temperature *T*. It is quantified by the similarity between $C_{ij}^{\prime }$ and $C_{ij}^{0}$, which should remain above a predetermined threshold, $Q_{\mathrm{thr}}$, that is,

(29) $${\langle Q\left(C^{\prime },C^{0}\right)\rangle }_{T}>Q_{\mathrm{thr}},$$

where the symbol 〈〉 indicates an average over the different chain configurations at the fixed temperature *T*. Finally, the third condition imposes a maximum number of attempts to reach the ground state, which is based on the idea that a suitable amino acids sequence should proceed smoothly and swiftly toward its ground state (fast folder), thus mimicking the funnel scenario expected for real protein folding [372].

The most important consequence of these results regards the emergence of ‘neutral networks’ in sequence space, representing sets of sequences, $S^{\prime }$ associated with $C^{\prime }$, having the same ground state, $C^{\prime }=C^{0}$ as the original sequence $S^{0}$, which is connected by point mutations. Therefore, a typical pair of distant sequences along the neutral network is indistinguishable from the behavior of just randomly chosen sequences. In other words, sequences that originated from the common ancestor, that keep the same ground state, can diverge in sequence space, yielding sequence similarities consistent with those found for random sequences. It is concluded that the thermodynamic stability of the ground state plays a crucial role for the emergence of neutral evolution in sequence space [371].

Based on this structural approach, a new model denoted the structurally constrained neutral evolution (SCNE) model has been developed [373], in which conservation (say $Q_{\mathrm{thr}}≳0.95$) of the thermodynamic stability of the wild-type native structure is imposed. Results from the SCNE model help understand the nature of neutral evolution trajectories in sequence space, suggesting new universal features for proteins. A prominent aspect is the existence of a broad distribution of the number of neutral mutations from one sequence to another, which is responsible for the breakdown of the traditional assumption of a Poissonian substitution process. Second, a much less known feature is the presence of strong correlations between the number of neutral mutations along an evolutionary trajectory, which cause the breakdown of self-averaging of the substitution process [373,374]. The correlations can be calculated using a so-called fluctuation analysis introduced within the framework of time series fractal analysis [375,376].

Further insight into the issue of predicting the native conformation from its sequence of amino acids has been achieved by realizing that for globular proteins, the contact map can be fully reconstructed from its principal eigenvector (PE) [377], in keeping with the fact that reconstructed contact maps allow in turn for an accurate representation of the 3D native structure (see, e.g., Figure 41). The results suggest that the single vectorial representation contained in the principal eigenvector of *C* is equivalent to the protein structure itself. Furthermore, it is also possible to map the sequence of amino acids to an hydrophobicity profile (HP), using a generalized hydrophobicity scale (interactivity scale), from which an optimal HP of a protein fold can be obtained. The latter is predicted to be strongly correlated with the PE of *C* [378]. This vectorial representation thus provides a sequence (HP)-to-structure (PE) map to be used in bioinformatics algorithms for structure comparison and sequence alignment and, more importantly, as an additional tool for protein structure predictions. Additional results and reference papers related to the issue of protein evolution and structure predictions are available in the literature [379].

## 4. Experimental Aspects of Polymers

Here, we deal with some experimental characteristics of polymers relevant to our discussions. In Section 4.1, we briefly review the chemical and structural properties of polymers, such as polypropylene (PP), and PTFE, and in Section 4.2, we explore the behavior of polymeric surfaces, such as polycaprolactone (PCL) and PTFE, after plasma treatments.

### 4.1. Chemical and Structural Aspects of Polymers

**Figure 42:**
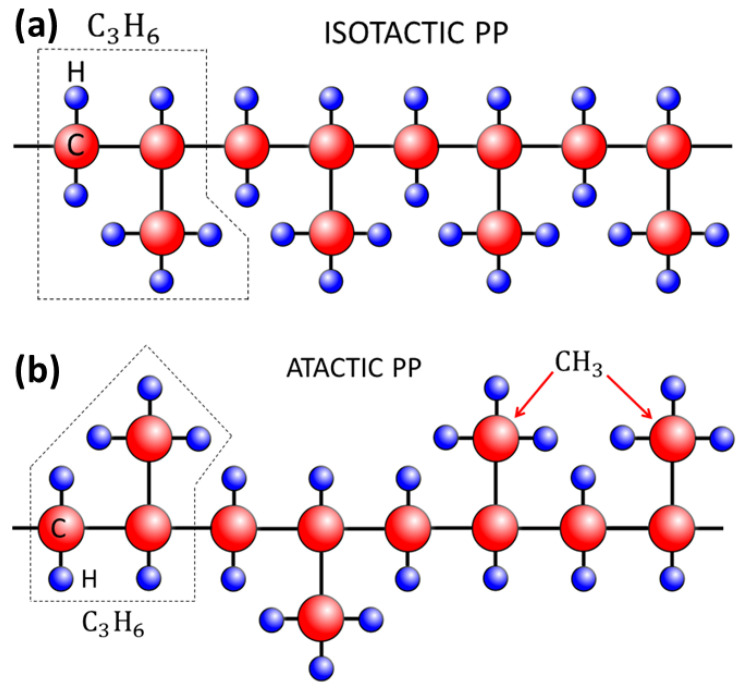
The two main forms of polypropylene of unit [$C_{3}$$H_{6}$]: (**a**) isotactic PP in which all the chiral carbons (${\mathrm{CH}}_{3}$) have the same configuration; i.e., they are on the same side of the chain. (**b**) Atactic PP, where the chiral carbons have random orientations. In syndiotactic PP, the chiral units alternate regularly along the chain (see, e.g., [384] for more details).

During the decade 1955–1965, significant developments to the then rapidly evolving field of polymer chemistry were achieved. They regard the polymerization of ethylene ($H_{2}$C=${\mathrm{CH}}_{2}$) by K. Ziegler [380], yielding polyethylene, and that of propylene (${\mathrm{CH}}_{3}$CH=${\mathrm{CH}}_{2}$) by G. Natta [381], in the isotactic and atactic forms of PP (see Figure 42). These discoveries lead to the method known as Ziegler–Natta catalysis (see, e.g., [382]), for which they shared the Nobel Prize in Chemistry in 1963. A third form of PP, known as syndiotactic PP, was also discovered [383]. More details on these systems can be found in the book by Roberts and Caserio [384].

The two PP configurations shown in the figure have very different behaviors when forming a polymeric material. Isotactic PP displays a conspicuous degree of crystallinity, while its atactic counterpart is amorphous (i.e., not crystalline). As a result, the physical properties of these materials are quite different. The isotactic one is a strong crystalline polymer which melts at 175 °C, being also practically insoluble in all organic solvents at room temperature. The atactic one is much softer, elastic, and rather soluble in suitable solvents. In contrast, polyethylene melts around 100 °C. Polypropylene is a very versatile polymer with many applications in almost all of the plastics employed today.

**Figure 43:**
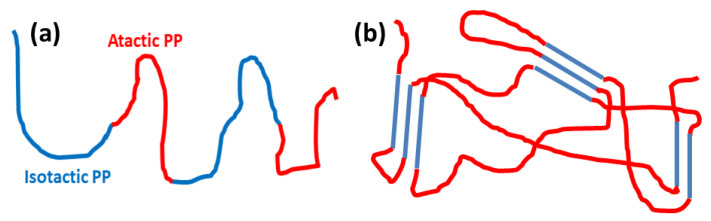
(**a**) Mixed polymerization connecting an isotactic PP string (blue lines) followed by an atactic one (red lines). (**b**) Illustration of a piece of material formed by mixed PP chains shown in (**a**). The blue straight lines represent isotactic PP crystalline zones, giving some rigidity to the structure, while the chosen proportion of atactic PP pieces provide the newtowrk with a desired elasticity.

Atactic PP polypropylene is not of commercial use, however under the presence of metallocene catalysts (see, e.g., [385]), it is expected that block polymers can be made containing alternating strings of both isotactic and atactic PP, as illustrated in Figure 43. Such mixed PP chains can eventually condense in such a way that isotactic parts meet each other, yielding little crystals inside the structure, thus creating sort of crosslinks that bring a partial rigidity to the network. In contrast, the atactic regions play the role of the elastic component of the structure, making it softer and amenable for applications as a good elastomer. Typically, plastic containers and bottles are made of a (50–60)% mix of isotactic–atactic PP.

To complement this experimental section, it is also useful to briefly review few theoretical papers which deal with polypropylene on both chemical as well as structural features.

Crystallization of isotactic PP via MC simulations: In [386], the authors model the temperature dependence of structural changes of isotactic PP near its melting temperature, using a coarse-grained model in a lattice with up to second nearest-neighbor interactions (see also [387,388,389,390]). At equilibrium, two different behaviors are found separated by a temperature $T_{\mathrm{PP}}=450$ K. For $T>T_{\mathrm{PP}}$, the chains display Gaussian disorder, while for $T<T_{\mathrm{PP}}$ helix formation becomes favorable even in the presence of environmental residual disorder. Below a second temperature, $T_{\mathrm{crys}}=385$ K, the system crystallizes. It is suggested that the polymeric material behaves similarly to a liquid crystal due to the long-chain helix configurations, which are locally emerging just above $T_{\mathrm{crys}}$.

MC simulations of interlamellar isotactic PP: In [391], MC simulations are reported to investigate the semi-crystalline interphase for isotactic PP, in which the $\alpha_{2}$ crystal phase of isotactic PP is used to model the structure of crystalline lamellae, and it is brought to equilibrium together with the remaining disordered domains [392,393,394,395]. The interactions between the different groups of the PP molecule are considered in detail via a united atom force field software. The work may shed some light into the quest of how crystal-amorphous interphases play a role in semi-crystalline polymer materials.

MD simulations of solvent effects on polyethylene oxide (PEO) and PP oxide (PPO) chains: In [396], the authors study using MD simulations the structural and dynamical properties of PEO and PPO chains at room temperature, both in a melt and at very high dilutions in different substances (see also [397,398,399,400]). The results are in very good accord with experiments, and conclusions can be drawn about the structural units which are formed in the solutions as found in nuclear magnetic resonance (NMR) studies.

MC simulations on effects of chain tacticity on polyethylene/PP blends: In [401], the authors search the mechanism responsible for the demixing of polyethylene/PP at equal concentrations, for the three PP tacticities at its melting temperature based on a coarse-grained chain model using MC simulations [402,403,404,405]. The model consists of both short- and long-range interactions, which is able to describe the transport behavior of the chain quite accurately. Evaluation of the degree of blend immiscibility was possible by calculating the interchain pair correlation functions, suggesting different tendencies of demixing according to the type of PP tacticity involved.

Single-chain folding of an isotactic PP chain: In [406], the authors study the folding behavior of a single isotactic PP chain via MD simulations, estimating the local rigidity strength of the folded chain. Care must be exercised in order to keep the underlying tacticity of the PP chain in its folded state. The study supports the expectation that angle and torsional potentials contribute to the speeding up of folding (see [407,408,409,410]).

Modified polyvinylidene fluoride (PVDF)–hexafluoropropylene (HFP) membranes: In [411], the authors report experiments on PVDF–HFP flat sheet membranes added with PEG, of different molecular weights and concentrations, which act as a pore former aimed at improving vacuum membrane distillation (VMD). The latter is a hybrid technology possessing several advantages over traditional desalination processes [412,413,414,415]. A 3D computational fluid dynamics (CFD) model is proposed for the validation of several properties relevant for comparison with experimental data.

MC simulations for complex polymer systems: In [416], the author reviews the Metropolis MC method to study complex polymer systems, showing that it remains a valid tool when compared with more elaborated full atoms MD simulations. The review describes applications to several systems in soft matter in which nanoscale structures play a central role. Examples include polymer nanocomposites and soft nanostructured materials, confined polymer chains, polymer rings and knots, crystalline systems, hydrogels and networks [417,418,419,420]. The extension of the method to non-equilibrium systems is also discussed together with some remaining open questions for future studies.

**Figure 44:**
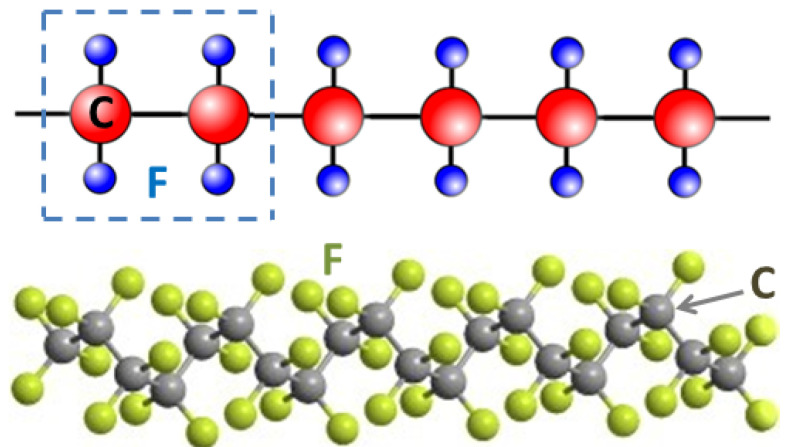
(**Upper panel**) The PTFE (commonly known as teflon) is built upon the tetrafluoroethylene (TFE) unit [$C_{2}$$F_{4}$] (dashed square). (**Lower panel**) The typical PTFE helix configuration.

Fluorinated polymers, such as PTFE (see Figure 44), are very popular due to their low chemical reactivity, suitable wettability and adhesion properties, making them very attractive to be employed in coating. PTFE is a thermoplastic polymer, insoluble in water, solid at room temperature, having a density of 2200 kg/$m^{3}$ and 600 K melting temperature. It is produced by the free-radical polymerization of tetrafluoroethylene (TFE), the fluorocarbon $C_{2}$$F_{4}$. It is the simplest perfluorinated alkene. PTFE is hydrophobic, exhibits low electric polarizability due to fluorine, and has an extremely low friction coefficient suitable to be used as lubricant. PTFE typical chemical inertness comes from the high strength of carbon–fluorine bonds, finding also useful applications in surgery and as a coating on catheters. Initially, PFAS were used in the production of PTFE, but they have been replaced by less toxic substances having a much lower impact on human health.

The crystal structure of PTFE: In [421], the author reviews experimental studies on the four crystal phases of PTFE, which are based on electron diffraction patterns recorded on glass photography plates, providing a unique overview of reciprocal space that improves on the limitations from X-ray data. The paper is a useful reference for highlighting unexplained features of the PTFE crystal structure, resolving some conflicting issues remaining from previous works (see also [422,423,424,425]).

Crystalline lattice strains measurements in phase IV PTFE: In [426], a discussion is presented on neutron diffraction measurements of crystalline lattice strains as a result of applied deformations on PTFE crystals [427,428,429,430]. The hexagonal unit cell can be identified from the observed six primary diffraction peaks of the corresponding plane deformations of the cell. It is possible to determine the associated chain compression moduli in different directions, resulting in values which exceed those of the bulk by (1–3) orders of magnitude, in agreement with the theoretical values for the PTFE chains moduli.

Optical and mechanical properties of thin PTFE films from gas phase deposition: In [431], the authors produce PTFE thin films by gas phase deposition, using electron-enhanced vacuum deposition (EEVD), which was also combined with a cold plasma technique. The experimental probes include IR spectroscopy, AFM and X-ray photoelectron spectroscopy, allowing the study of the film structure, morphology and composition. Several quantities are obtained such as the film surface roughness, which depends on gas pressure and plasma parameters, the anisotropy of the refractive and extinction indices measured by spectral ellipsometry, hardness and Young moduli, and the thermal stability of the films [432,433,434,435]. The film contact angle equals that of bulk PTFE. It is concluded that PTFE molecules are mostly oriented perpendicularly to the substrate surface.

### 4.2. Fractal Aspects of Polymeric Surfaces

Polymeric materials surfaces can be modified, or altered in a well-defined fashion, by using chemical methods. This chemistry often requires the use of toxic elements, and a way to alleviate this issue relies on the application of gaseous plasma techniques (see Section 2.2 and Figure 14). Different plasma configurations and plasma setups are used, and the treated surfaces can develop a prevailing wetting character ranging from super-hydrophilic to super-hydrophobic, depending on the type of polymer considered. The resulting chemical composition of the new surface can be studied for instance by means of X-ray photoelectron spectroscopy. In some cases, the hydrophilic behavior may be due to the surface polymer bond breaking induced by the presence of ultraviolet radiation rather than the chemical functionalization of some reactive species (see, e.g., [436,437]).

**Figure 45:**
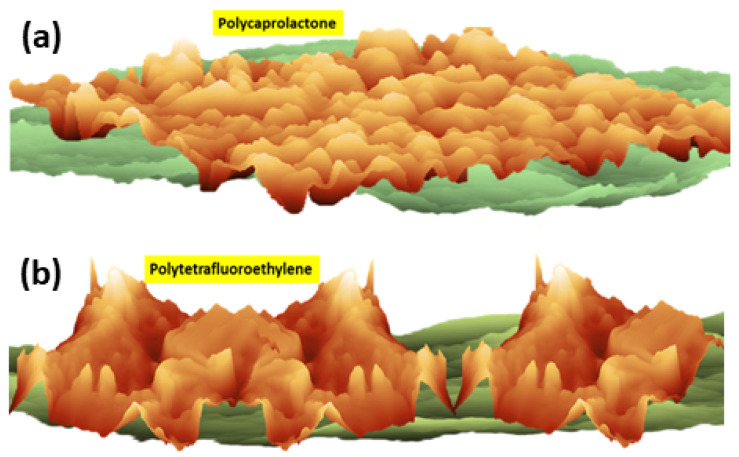
Illustration of non-treated (NT) (green color) and plasma-treated (PT) (red color) samples of polymeric surfaces, depicted roughly on scale. (**a**) PCL, the vertical scale for the NT sample is 60 nm, while for the PT one, it is 120 nm. (**b**) PTFE, the NT scale is 70 nm, and the PT one 790 nm.

Plasma techniques have become very popular for their versatility and reproducible results. Some examples are shown in Figure 45 in the case of PCL (upper panel) and PTFE (lower panel) (see [438] for more details). In the following section, we discuss the effects of plasma treatment to the wetting properties of some polymeric materials’ surfaces.

## 5. Applications in Materials Science

The applications to materials science we discuss here regard plasma treatments of polymeric surfaces, examples of which concern PTE, PTFE, PP, and PCL. The effects of plasmas, typically cold ones, are described in terms of modifications in the wettability properties of the surfaces, which find many useful applications in industry (Section 5.1). A second theme of interest is the fabrication of new thin films by polymer attachment to a surface, which has been activated using plasma techniques, allowing for the presence of suitable sites on which a polymer chain, in this case PEG, becomes locked (Section 5.2).

### 5.1. Plasma Treatment: Wetting Properties and Surface Fractal Scaling

**Figure 46:**
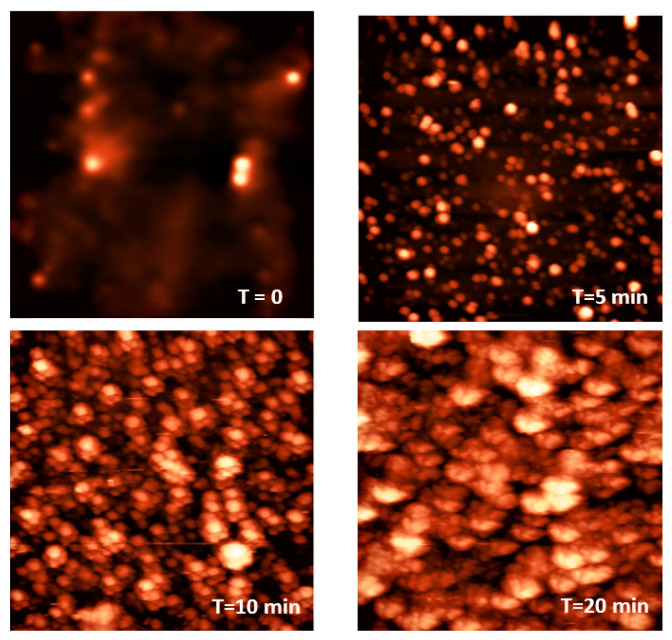
AFM images of PET surfaces after oxygen plasma treatment of different time durations, T=(0,5,10,20) [min] (adapted from [439]).

Plasma-treated polymeric materials surfaces are illustrated in Figure 46 in the case of PET, where oxygen gas is used at working powers of a few hundred Watts [439]. Based on these experiments, it is possible to draw conclusions about specific properties of the modified surface, in particular at the nanoscale, regarding wettability and surface new morphology. Typically, AFM and scanning electron microscopy (SEM) techniques are employed to extract relevant structural information on the treated surfaces. Remarkably, in most cases, such modified surfaces present fractal scaling behavior over the (10–100) nm range, which is however found to be already present in the non-treated surface too but having lower amplitude fluctuations extending on a smaller range of length scales [438].

**Figure 47:**
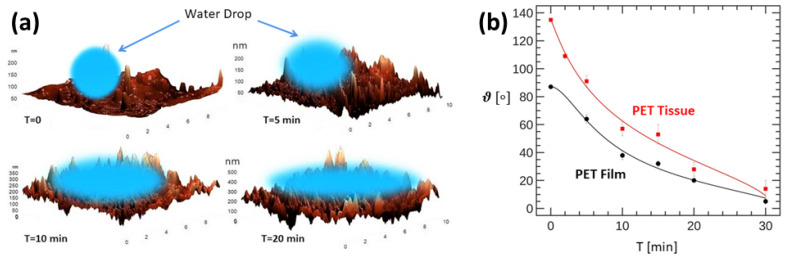
(**a**) The treated PET surfaces shown in Figure 46 illustrating the hydrophilicity effects on a virtual water droplet positioned on top of the material for different treatment times *T* [min]. The original surface is at T=0, and the vertical scales are: (200, 200, 350, 500) nm. (**b**) The variation of the contact angle with treatment time expected theoretically (lines) and compared with experimental results (symbols). The images correspond to films of size 10μm × 10μm. In the case of the PET film, the fractal parameters are H=0.82±0.02 and $d_{S}=2.18\pm 0.02$, while for the PET tissue, they become H=0.46±0.02 and $d_{S}=2.54\pm 0.02$ (see Equation (31)) (adapted from [439]).

In the following, we consider the wetting properties of material surfaces by briefly reviewing a fractal surface model aimed at describing the dependence of the contact angle, $\theta_{c}$, on the plasma treatment duration time, *T*. In the present notation, *T* should not be confused with temperature, since the latter does not play a role in our discussions here. The wetting behavior of surfaces is quantified by measuring the contact angle between a little water droplet gently deposited on top of the material. According to the Young equation, the contact angle $\theta_{c}$ between a fluid (L) and a solid (S) interface, immersed in a gas phase (G), can be written as [438,439]

(30) $$\cos\theta_{c}\left(T\right)=A\;\left(1-B\;F(T)\right),$$

with $A=\gamma_{\mathrm{SG}}/\gamma_{\mathrm{LG}}$, $B=\gamma_{\mathrm{SL}}/\gamma_{\mathrm{SG}}$, where $\gamma_{ij}$ represents the interfacial surface tensions between phases *i* and *j*. The function F(T) has been introduced to account for the variation of $\cos\theta_{c}$ with treatment time *T*. Notice that the original Young equation corresponds to taking F(T)=1. Qualitatively, Equation (30) can be interpreted in terms of chemical effects between the interacting surfaces via the surface tensions $\gamma_{ij}$ and physical ones due to the varying surface roughness as a function of *T*. This separation appears reasonable at first sight, but it needs to be carefully validated with experimental data. Illustrative examples of the observed behavior of a water droplet located on the PET-treated surfaces are reported in Figure 47a.

Interestingly, the form of F(T) is rather simple, containing explicit information about the fractal scaling of the surfaces as a function of time *T*. The continuous lines shown in Figure 47b are obtained if one defines the roughnes function F(T) as follows,

(31) $$F\left(T\right)=\frac{1}{1+R(T)},\;\mathrm{with}\;R\left(T\right)={\left(\frac{\sigma (T)-\sigma (0)}{\sigma (0)}\right)}^{d_{S}}≃{\left(\frac{T}{T_{0}}\right)}^{d_{S}H},$$

where σ(T) is the standard deviation of surface amplitude fluctuations, $d_{S}=3-H$ is the fractal dimension of the surface, *H* is the Hurst exponent of linear profiles of the surface, yielding the behavior $\sigma \left(T\right)≃\sigma \left(0\right)+\sigma \left(0\right){\left(T/T_{0}\right)}^{H}$, and $T_{0}$ is a characteristic time for plasma treatment [438,439]. According to its definition, R(0)=0, and $\cos\theta_{c}\left(0\right)$ is given by the surface tension values alone. In applying Equation (30), it is assumed that the latter (i.e., the chemistry of the process) remains unchanged upon plasma treatment. In other words, within this approximation, the γ values do not change with the plasma treatment. This assumption needs to be verified in future experiments, but in the cases discussed so far [438,439], it seems to hold quite accurately.

It is useful to make contact with the standard approach to wetting based on the Cassie–Baxter equation for the contact angle, $\theta_{c}$, between a liquid droplet and a two-component solid surface, characterized by displaying fractional surface areas $f_{1}$ and $f_{2}$, with $f_{1}+f_{2}=1$. Each surface component has its own contact angle denoted as $\theta_{1,2}$, respectively, yielding the relation,

(32) $$\cos\theta_{c}=f_{1}\;\cos\theta_{1}+f_{2}\cos\theta_{2}.$$

Now, for a fully hydrophobic second component, i.e., when $\theta_{2}=180^{\circ }$, one has the known result $\cos\theta_{c}=f_{1}\;\cos\theta_{1}+f_{1}-1$. Finally, in the case of a single component, $f_{1}=1$, we arrive at the Wenzel regime, where the surface is characterized by a roughness r≥1 [440,441,442], so that $\cos\theta_{c}=r\;\cos\theta_{1}$. The Wenzel limit is appropriate for rough hydrophilic surfaces where $\theta_{c}$ decreases when *r* increases. For instance, in the examples shown in Figure 47, one has for non-treated samples, i.e., r=1, $\theta_{c}≃89^{\circ }$ for PET films, and $\theta_{c}≃135^{\circ }$ for PET tissues (see [439]), suggesting that NT samples are moderately hydrophilic.

To make contact with Equations (30) and (31), notice that the roughness parameter *r* can be written as the ratio between the surface area of the treated material, S(T), and the non-treated one, S(0), i.e., r=S(T)/S(0)≡1+aR(T), where *a* is a parameter. Thus, one finds $\cos\theta_{c}=\cos\theta_{1}+a\cos\theta_{1}\;R\left(T\right)$, which is denoted as the fractal-Wenzel model [439]. The model in Equation (30) can be expanded up to the first order in R(T), yielding $\cos\theta_{c}≃A\left(1-B\right)+AB\;R\left(T\right)$, which coincides with the fractal-Wenzel result when $A\left(1-B\right)=\cos\theta_{1}$, and $AB=a\cos\theta_{1}$. Thus, a fractal-Wenzel equation describes a hydrophilic homogeneous fractal surface in the limit of low roughness, corresponding to short treatment times in the more general expression Equation (30). Further generalization of the latter to the case of hydrophobic surfaces, as in the case of PTFE, is possible yielding a very good agreement with the experimental results [438].

### 5.2. PEG: Thin Film Deposition via Reptation Dynamics

**Figure 48:**
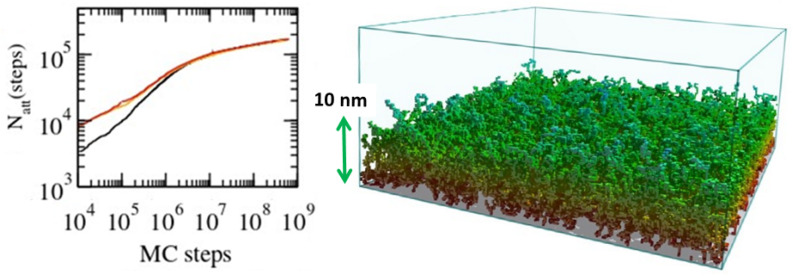
Monte Carlo simulations of SAW chains deposition on a flat plasma-activated substrate. (**Left panel**) Number of attached monomer units, $N_{\mathrm{att}}=N\;N_{\mathrm{chain}}$, with $N_{\mathrm{chain}}$ being the numer of attached chains of *N* monomers each, as a function of MC steps. Here, N=68 (black line), N=114 (orange line) and N=200 (red line). The data collapse above $10^{6}$ steps suggests that $N_{\mathrm{att}}$ becomes independent of *N* asymptotically. (**Right panel**) An example of a simulated PEG thin film, on a substrate of 200 × 200 sites and system height 100 sites, with a lattice constant $a_{0}=0.36$ nm, and SAW chains of N=200 monomers. The total number of attached chains is 834 in this example. The color scale is proportional to monomer height (adapted from [444]).

Thin films of linear polymers deposited on a surface have many useful applications in the biomedical sector. Among them, PEG plays a prominent role as a biocompatible coating displaying reduced protein adsorption and cell adhesion [443]. The deposition of thin films of PEG chains on a substrate necessitates the presence of active sites on the surface on which one of the ends of a PEG chain can become attached. The active sites are carboxyl (COOH) groups randomly dispersed onto a silicon wafer by means of chemical or physical processes. Plasma techniques are suitable for efficiently achieving this so-called pre-functionalization of the surface. Although this phenomenon was known for quite some time, the questions of how the PEG chains diffuse in solution and become finally attached to a surface have remained unanswered. In [444], thin films of PEG have been created on carboxyl-activated silicon wafers using chains of a fixed number of monomers, *N*. Different experiments were performed for samples with N=68 and N=114 monomers each. In order to understand the mechanism of PEG chain deposition and thin film formation, MC simulations of reptating SAWs in a simple cubic lattice were performed (see Figure 48), where the substrate has a density of active sites in accord with experiments [444].

It is found that less than 20% of total active sites (≃2 sites/${\mathrm{nm}}^{2}$) are effectively occupied by attached chains, corresponding to less than 5% of the total available substrate sites. Scaling arguments suggest a universal power-law dependence of film density, ρ(N), as a function of the number of polymer units *N*, i.e., $\rho \left(N\right)≃\rho_{0}/N^{\nu }$, with $\rho_{0}≃5$ g ${\mathrm{cm}}^{-3}$ and ν≃0.6, the latter consistent with the Flory exponent for SAWs in 3D (see Section 3.2).

The theoretical predictions of the dependence of film density on PEG length *N* and the prefactor $\rho_{0}$ on the density of carboxyl active sites [444] are important for the development of suited fabrics for specific biomedical applications. Further experimental research is still required to clarify several open issues such as deposited PEG film mass and density, and the polymer attaching dynamics to validate the model predictions of the film growth time scales determining the PEG deposition processes.

PP films on silicon wafers: In [445], the authors consider PP film growth on silicon wafers based on a simple and versatile method favoring a covalent attachment to produce ultrathin films [446,447,448]. The attachment sites are created by employing perfluorophenyl nitrenes produced by the thermolysis or photolysis of perfluorophenyl azides. A careful development of film–wafer covalent bonds allows the fabrication of both crystalline and amorphous PP films and, as an extension of the method, an elastomeric PP copolymer is also successfully realized.

## 6. Applications in Biology: DNA Chains Within the Cell and Aminocyanines

We consider applications to the DNA chain structure in particular regarding the occurrence of ‘i-motifs’ (Section 6.1). The question of DNA transcription is discussed in some detail in Section 6.2, where contact is made with the reptation method for generating SAWs configurations in a lattice discussed in Section 3.2.4. Finally, recent results on the promising use of aminocyanines are discussed in Section 6.3.

### 6.1. DNA Chains: I-Motifs

**Figure 49:**
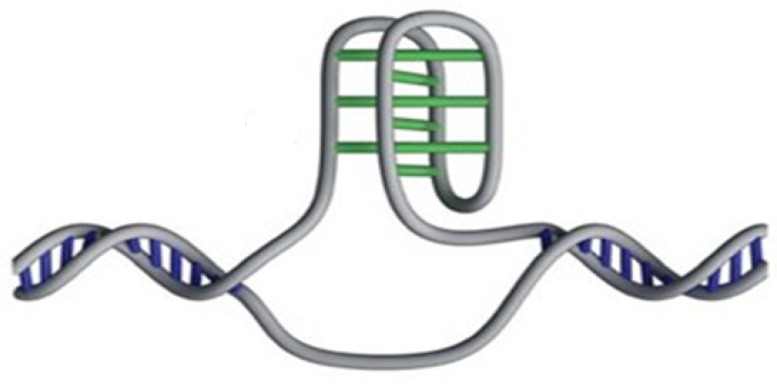
Schematic illustration of an i-motif: a piece of DNA with a knot inside its structure. Such openings make the transcription process possible, providing key regulatory functions (adapted from [449]).

The DNA consists of two connected chains in space typically forming a perfect closed double helix. Each chain of DNA consists of a backbone upon which a sequence of nucleotides or bases (A = adenine, T = thymine, and G = guanine, C = cytosine) are attached. The two chains are connected by bonds between pairs of opposite bases, (AT) and (GC) pairs. Thus, the sequence of bases on one chain corresponds exactly to the one of the second chain, in which A is replaced by T and G by C. Remarkably, often C bases pair with each other, i.e., forming a (CC) link instead of a (CG) one, yielding a deformation and partial opening of DNA, thus creating a protrusion denoted as an i-motif. This is likely to occur within cytosine-rich regions of the genome. Therefore, DNA is prone to contain i-motifs or knots along the chain (Figure 49), leading to the suspicion that they could be important regulators of the genome in relation to which genes are on or off [449]. However, both the locations and number of knots were not identified.

Recently, a thorough examination of DNA led to the conclusion that such i-motifs are much more common than presently believed. Some 50,000 knot-type structures were found along DNA strands [450]. This work is relevant for improving the predictions about the exact locations of i-motifs within a DNA strand, which in turn can help the development of new strategies for the treatments and perhaps even the prevention of diseases.

### 6.2. DNA Chains: Transcription Process Within the Cell

The problem of DNA transcription has also attracted a great deal of attention among theoreticians. As a good starting point to this problem, one considers coarse-grained polymer models studied by either MC or MD simulations. One popular approach is the so-called facilitated diffusion model, taking place within a closed volume, mimicking the nucleus of a living cell, within which a long chain, representing a strand of DNA, is confined. In facilitated diffusion, one considers a point-like transcriptor factor, representing a protein that binds to DNA in appropriate locations (targets), that mimics the important task of genetic information transcription from DNA to messenger RNA. Such a particle performs, alternatively and randomly, both a 3D random walk in the free cell space and a random walk along the DNA chain until it reaches the target. The latter represents the finite coded sequence to be read. The MC simulations in [451] performed in the continuum suggest that facilitated diffusion is most efficient in the presence of an elastic DNA chain, which appears relevant for in vitro studies and diffusion on yeats chromatin.

**Figure 50:**
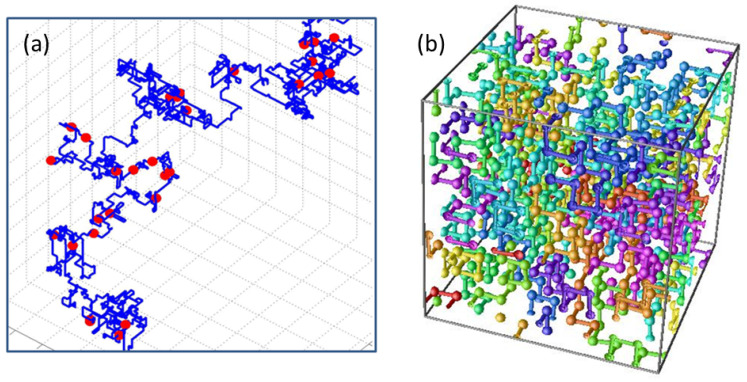
(**a**) Transcription process: Simulation of a long DNA (10,000 units) in a cubic lattice of linear size 100 lattice units, using the reptation method. The red circles represent the transcription targets. A single transcriptor executes a 3D random walk inside the free lattice space and can perform a 1D random walk along the blue path. Once moving along the DNA chain, the transcriptor can leave its 1D walk and move to the 3D free lattice if the target has not been found. Its remains along the DNA for a finite number of steps only, in keeping with the faster diffusion in the free space that accelerates the target-finding process. (**b**) Simulation of 20 SAW chains with N=20 monomers each, using the reptation method, within a cubic lattice of linear size L=10 lattice units with PBC, yielding a total chain occupation of 40% inside the confining cube. Courtesy of Francesco Marini.

An illustration of a long chain inside a cube defined in a simple cubic lattice is shown in Figure 50a. The chain configuration was obtained using the reptation method in 3D by letting a polymer chain grow inside the finite volume consisting of hard walls. When the leading SAW chain end touches the wall, it retreats as explained in Section 3.2.4 and performs another growth attempt. In 3D, the growing of SAW chains is much easier than in 2D due to the less attrition present in the system. Notice also the exceedingly large fraction of cell space left unoccupied by the single SAW chain. Indeed, the method allows for simulating many SAWs at the same time, making the model much more realistic (see example in Figure 50b).

In [452], the authors describe the process of gene transcription target, by means of MC simulations in a simple cubic lattice, using many proteins that start their motion from a single lattice position, $\vec{\ell }$, simultaneously. They study first-passage times of proteins touching a single DNA chain and average them over several configurations. The mean first-passage time is then studied as a function of the distance from the starting position $\vec{\ell }$ and a single DNA target position and the resulting concentration of proteins employed. The results suggest the possible relevance and importance of colocalization of the initial protein search inside real biological cells. Additional research has been performed on a simpler 1D model of DNA in which facilitated diffusion occurs in 3D space undergoing anomalous diffusion to mimic the complex transport behavior of a many-protein system [453].

### 6.3. Aminocyanines: A Physical Method to Kill Cancer Cells

**Figure 51:**
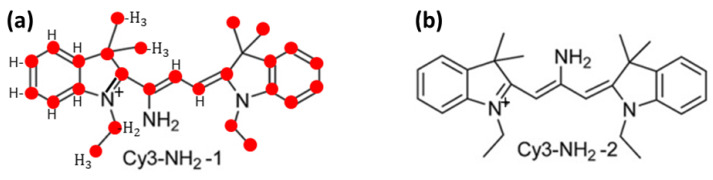
The structure of aminocyanine dyes Cy3-${\mathrm{NH}}_{2}$-(1,2). The red circles represent C atoms, while few H atoms are indicated for illustration. (**a**) Cy3-${\mathrm{NH}}_{2}$-1: The unit ${\mathrm{NH}}_{2}$ is close to $N^{+}$. Here, a 3C-polymethine chain is transformed into a 2C-polymethine chain in the center of the molecule, and the third C is attached to the amine group ${\mathrm{NH}}_{2}$ rather than to a H. (**b**) Cy3-${\mathrm{NH}}_{2}$-2: The unit ${\mathrm{NH}}_{2}$ is at the center of the polymethine chain (adapted from [454]).

Polymethines are compounds made up from an odd number of methine groups (=CH–) bound together by alternating single and double bonds, as, e.g., in piperylene or 1,3-pentadiene ${\mathrm{CH}}_{3}$[–CH=CH–CH=]${\mathrm{CH}}_{2}$ indicated within square brakets. A chain with alternating single and double bonds can form a conjugated system, which is characterized by the presence of connected π-orbitals carrying delocalized electrons along the molecule. Methine groups can become closed into a single ring as in benzene (3[=CH–CH=]≡$C_{6}$$H_{6}$), having an aromatic character. For even number of methine groups, the compounds are called polyenes. Finally, cyanines (Cyn-Residue) are synthetic dyes and belonging to a polymethine group. In case the residue is the amino group ${\mathrm{NH}}_{2}$, the compound is called aminocyanine (see the example of Cy3-${\mathrm{NH}}_{2}$ in Figure 51 and [454] for more details). Such polymethine–carbocyanine dyes, known also as aminocyanine fluorophores, are widely utilized as fluorescence probes specially for obtaining images of biological targets in vivo due to their remarkable photophysical properties.

In [455], the authors study a remarkable application of aminocyanines to kill cancer cells in vitro using near-infrared light (NIR) in the 650–900 nm wavelength range. The basic idea is to exploit the significantly high electron–phonon couplings typical of these dye molecules. The NIR light produces the excitation of plasmon modes, which are collective electronic states extending over the whole molecule [456]. In the case of aminocyanines, the strong coupling between plasmons and collective atomic motions called phonons result in a mechanical response of the molecule which is used to destroy the membrane protecting a cancer cell. The authors called this effect vibronic-driven action (VDA), and the mechanical disruptive behavior of the aminocyanine is referred to as a molecular jackhammer. It is expected that a cell cannot avoid necrosis by developing resistance to such jackhammers, suggesting that they can be used with success to treat cancer cells which have developed deep inside the skin, perhaps reaching several cm in depth. In addition to experiments in vitro, they obtained a 50% tumor-free efficacy in mouse with melanoma, thus opening the door for future studies aimed at treating cancer cells in vivo by exciting aminocyanines with NIR light.

Recently, a new discovery on unexpected double bonding in carbon composites has been reported on a class of geometrically distorted ϕ-bonded molecules [457]. These are known as anti-Bredt olefins (ABOs), the latter were theoretically known since 1924 but expected to be difficult or even impossible to realize. The authors discuss a new strategy to deal with these elusive compounds displaying significant distortion due to the presence of geometrically constrained ϕ-bonds, offering a method to produce geometrically distorted alkenes to be used in future synthetic applications.

## 7. Concluding Remarks

We have reviewed theoretical aspects of linear polymers modeled by SAWs in regular lattices, and in both deterministic and random fractal structures, which should provide the reader with the state of the art on SAWs in those environments. The use of square and simple cubic lattices simplifies the numerical studies to a large extent, in keeping with the universality class of SAWs in 2D and 3D cases. Fractal structures are of central interest here, since they may play a prominent role in many important applications, in particular when some type of complex structural disorder yields an unusual behavior of the polymeric system. By studying simple fractal models, one can understand how the statistical properties of SAWs are modified in the presence of scale invariance. Clearly, the easiest cases to consider are deterministic fractals which lack structural disorder before entering the realm of fractal disordered structures as in the case of critical percolation clusters. These studies provide a benchmark for the fractal behavior of polymers, which can be used as guidelines to build realistic models aimed at describing experimental observations of real polymeric systems.

Specifically, we have discussed in detail the geometrical aspects of SAWs in regular and fractal lattices with emphasis on the two types of metrics required to determine the structural functions of single chains, such as the distribution functions of the end-to-end distance of *N*-steps SAWs, both in Euclidean space, P(r,N), and in topological or chemical space, P(ℓ,N), where *ℓ* is the topological distance between the chain ends (see, e.g., Figure 30). While in regular lattices, and deterministic fractals modeled by Sierpinski fractal carpets in 2D and the fractal sponges in 3D (see Figure 26), both metrics are equivalent, i.e., r≃ℓ, in critical percolation clusters they scale differently, $r≃\ell^{d_{\min}}$, where $d_{\min}>1$ is the fractal dimension of the shortest path. The fact that $d_{\min}>1$ yields a multifractal behavior of SAWs in such disordered systems. However, one can study the statistical properties of the chains in *ℓ*-space, which offers the possibility of reducing considerably the fluctuations of the measured quantites of interest, while the behavior of SAWs in real space can in most cases be obtained from their counterparts in chemical space.

The question arises regarding whether the deterministic fractals discussed here (as those in Figure 26) are just of theoretical interest, or they may have some experimental realization. We have suggested that their structures may become experimentally accessible as in the case of the 2D carpet, which may be created by deposition techniques using a fractal mask. The 3D sponge could be built using 3D-printing techniques, the latter representing for instance a regular type of porous material. From a theoretical point of view, they offer the possibility of studying statistical properties of linear polymers in scale-invariant systems accurately, thus allowing for the determination of critical exponents which may differ from their standard values in regular lattices. In particular, much effort is still needed to improve on the values of the exponents in the case of percolation clusters (see Section 3.2.3). Furthermore, future work is envisaged to have a complete picture on the transport properties of diffusing chains within fractals. To this end, applications of the reptation method (Section 3.2.4) should be explored, as it may yield accurate and interesting results. In Figure 31, we have provided an example demonstrating the excellent performance of the method in regular lattices regarding static properties. We have used reptation also on deterministic fractals [344] (Sierpinski carpet (2D) and Sierpinski sponge (3D) in Section 3.2.2) and the numerical results were summarized in Table 2.

The reptation method is suitable for generating SAWs configurations in arbitrary environments. Yet it can be easily extended to a many-chain system, also in the presence of geometric constraints inside the host’s structure. The problem of creating an ensemble of reptating chains, briefly discussed in Section 3.2.4, has not been studied systematically, and future work on this issue is certainly welcome. The applications are countless due to the many different types of polymer melts one can consider, including heteropolymers where different types of monomers are present, yielding a complex interaction-energy landscape. The method can be applied to study a melt at thermal equilibrium using MC rules and the outcome compared with more standard simulations methods of a many-chain system (see Section 2 for examples). The method can be used to find a suitable initial configuration of a many-polymer system, as we briefly discussed in Section 6.2 in the case of periodic boundary conditions. Indeed, homopolymer chains can be taken as the backbone of more complex polymers and use their location to start integrating the equations of motion of the more realistic polymers within an MD scheme.

Protein folding and protein sequence evolution have been discussed in Section 3.3 based on simple SAWs heteropolymer models of proteins in the simple cubic lattice, where the basic concepts of folding and the characteristic structures of proteins are illustrated. The last two sections of the work are dedicated to applications in materials science and biology. The former deals with a discussion of the important role that fractal features play for determining the wetting properties of plasma-treated polymeric materials surfaces. The basic concepts underlying the main equations used for describing wetting are explained, and contact is made with experimental data. Further experimental investigations are welcome to extend or modify the range of validity of these theoretical predictions. In addition, the interesting issue of modeling the growth of thin films by polymer deposition is mentioned, but many questions remain open such as a detailed numerical study on the scaling effects of the density of active sites created on the substrate on which the chains become attached. Experiments should be designed to investigate other polymers by exploring possible modifications of the reptation approach used in the case of PEG. Finally, applications in biology are discussed dealing with long linear polymers, such as DNA, confined within a finite domain, such as a cell. The material examined here is just a short introduction to the subject, and much more work can be conducted to establish a full picture on the issue of a high-density polymer ensemble confined within closed boundaries. We hope that the present review can be of help to explore new avenues in polymer science in different areas of physics, chemistry and biology.

## Figures and Tables

**Table 1:** The exponents describing SAWs in 2D and 3D in regular systems. The values for the coordination numbers μ(d) are for the square and simple cubic lattices, respectively. For comparison, the exponents in 4D are reported, without taking into account logarithmic corrections, and the coordination was taken as μ(4)=2d−1=7. The values for ($g_{1}$, $g_{2}$, δ) have been calculated from the exponents (ν, γ, *d*) using Equations (10)–(12).

	2D	3D	4D
ν	3/4	0.587597(7)	1/2
γ	43/32 (1.34375)	1.15695300(95)	1
μ	2.63815853032790(3)	4.6840401(50)	7
$g_{1}$	11/24 (0.45833…)	0.26711(2)	0
$g_{2}$	5/8 (0.625)	0.25664(5)	0
δ	4	2.42481(4)	2

**Table 2:** The exponents describing SAWs embedded in a Sierpinski carpet (2D) and Sierpinski sponge (3D) of fractal dimensions, $d_{f}≃1.89278$ and $d_{f}≃2.872683$, respectively. The values of the exponents are from exact enumeration results [348] except for $g_{1}$, which have been taken from the MC simulation results based on the reptation algorithm [344]. The values for μ can be estimated quite accurately using the relation, $\mu =\left(d_{f}/d\right)\mu \left(d\right)$ [348], where μ(d) is the coordination number of the regular lattice. This relation yields, 2.50(2) in d=2 and 4.26(2) in d=3, which are in very good agreement with the measured values.

	2D	3D
$d_{f}$	ln8/ln3	ln20/ln3
ν	0.75(5)	0.58(3)
γ	1.23(4)	1.36(3)
μ	2.515(15)	4.26(2)
$g_{1}$	0.52(5)	0.33(5)
$g_{2}$	1.41(8)	0.10(5)
δ	4.00(30)	2.65(50)

**Table 3:** The exponents describing the structural properties of the backbone of critical percolation clusters in 2D and 3D [352]. Values of $d_{\min}$ are from [319]. Above the critical dimension, $d_{c}=6$, structural loops are irrelevant, and the backbone behaves like random walks, yielding $d_{\min}=2$, $\tilde{\nu }=1/2$, δ=2, $d_{\ell }^{B}=1$, and $d_{f}^{B}=2$.

	2D	3D
$d_{\min}$	1.13077(2)	1.3756(6)
$d_{\ell }^{B}$	1.45(1)	1.36(2)
$d_{f}^{B}$	1.64(1)	1.87(2)
$\tilde{\nu }$	0.88435(2)	0.7270(6)
$g_{1}^{B}$	1.30(20)	1.02(20)
$g_{2}^{B}$	1.97(20)	0.59(20)
δ	8.6468(2)	3.662(6)

**Table 4:** The exponents describing the structural properties of SAWs embedded in the backbone of critical site percolation clusters in 2D and 3D, obtained using EE calculations [330]. The relation $g_{2}^{r}=g_{2}^{\ell }\;d_{\min}$ does not appear to hold in this case, at least within the accuracy of these EE studies, and the values reported here have been obtained directly from the simulation results. Also, the des Cloizeaux relation, $g_{1}^{r}=\left(\gamma_{1}-1\right)/\nu_{r}$, shows significant departures from the reported numerical results, in particular in 3D. The numerically obtained values of $\mu_{1}$ are, however, consistent with the relation, $\mu_{1}=p_{c}\;\mu$ (see Table 1 for μ), with $p_{c}=0.5927$ (2D), and 0.3116 (3D).

	2D	3D
$\gamma_{1}$	1.34(5)	1.29(5)
$\gamma_{0}$	1.26(5)	1.19(5)
$\mu_{1}$	1.565(5)	1.462(5)
$\mu_{0}$	1.456(5)	1.317(5)
$\nu_{\ell }$	0.890(5)	0.910(5)
$\nu_{r}$	0.778(15)	0.66(1)
$\nu_{r}=\nu_{\ell }/d_{\min}$	0.787(10)	0.662(6)
$g_{1}^{\ell }$	0.45(10)	0.66(15)
$g_{1}^{r}$	0.51(10)	0.91(15)
$g_{1}^{r}=g_{1}^{\ell }\;d_{\min}$	0.51(11)	0.91(20)
$g_{2}^{\ell }$	1.60(16)	1.95(17)
$g_{2}^{r}$	1.26(40)	2.96(18)
$\delta_{\ell }$	9.5(5)	12.0(5)
$\delta_{r}$	4.85(20)	3.10(20)

## Abbreviations

ABS	acrylonitrile butadiene styrene
ACN	acrylonitrile
AI	artificial intelligence
AIMD	ab initio molecular dynamics
AFM	atomic force microscopy
AFMIR	infrared (IR) laser-based AFM
ABO	anti-Bredt olefin
AMSilica	acrylamide functionalized silica
APS	ammonium persulfate
ATP	adenosine triphosphate
BB	Bergström–Boyce model
BCP	block copolymer
BNNS	boron nitride nanosheet
BPADA	4,4′-bisphenol A dianhydride
BAPB	4,4′-bis(4-aminophenoxy)biphenyl
CFD	computational fluid dynamics
CNT	carbon nanotube
CFD	computational fluid dynamics
cPAM	crosslinked polyacrylamides
CROW	chemical retrieval on the web
CRR	constraint release Rouse
DNA	deoxyribonucleic acid
DDS	drug delivery system
DFT	density functional theory
DPD	dissipative particle dynamics
EE	exact enumeration
EEVD	electron-enhanced vacuum deposition
FCC	face-centered cubic
FDM	fused deposition modeling
FP	frontal polymerization
FRP	fiber-reinforced polymer
GG	glycylglycine
GIP	glucose-dependent insulinotropic polypeptide
GLP	glucagon-like peptide
HCP	hexagonal closed packed
HDDMA	1,6-hexanediol dimethacrylate
HFP	hexafluoropropylene
HiG	hierarchical grid
HP	hydrophobicity profile
HSP	Hansen solubility parameter
IC	interaction chromatography
iIC	incipient infinite cluster
IDS	intrinsically disordered proteins
IoT	Internet of Things
ISP	in situ polymerization
KMC	kinetic Monte Carlo
LC	liquid chromatography
LG	liquid wood
LIB	lithium-ion battery
LL37	trifluoroacetate salt
LPI	liquid phase infiltration
MBB	molecular bottlebrush
MC	Monte Carlo
MD	molecular dynamics
MMM	mixed-matrix membrane
MPCD	multiparticle collision dynamics
MPD	m-phenylenediamine
mRNA	messenger RNA
MWD	molecular weight distribution
NBR	nitrile butadiene rubber
NIR	near infrared
NMR	nuclear magnetic resonance
NP	nanoparticles
NT	non-treated
PA	phthalic anhydride
PAM	polyacrylamide
PAMAM	polyamidoamine
PANI	polyaniline
PASA	poly(aspartic acid)
PASJD	plasma-assisted supersonic jet deposition
PBI	polybenzimidazole
PBC	periodic boundary conditions
PCF	pair correlation function
PCL	polycaprolactone
PCSAFT	perturbed-chain statistical associating fluid theory
PDB	Protein Data Bank
PDMS	poly(dimethylsiloxane)
PE	principal eingenvector
PEEK	polyetheretherketone
PEG	polyethylene glycol
PEI	polyetherimide
PEM	proton exchange membrane
PEMFC	polymer electrolyte membrane fuel cell
PEO	polyethylene oxide
PES	polyethersulfone
PET	polyethylene terephthalate
PETG	polyethylene terephthalate glycol
PEV	polyethylene vanillate
PFAS	per- and polyfluoroalkyl substances
PGA	poly(glutamic acid)
PHA	polyhydroxyalkanoate
PI	polyimide
PLA	polylactic acid
PLL	polylysine
PMM	polymeric metamaterials
PMIA	poly-m-phenyleneisophthalamide
PP	polypropylene
PPI	polypropylene imine
PPC	polypropylene carbonate
PPM	porous polymeric material
PPO	polypropylene oxide
PRISM	polymer reference interaction site model
PS	polystyrene
TFE	tetrafluoroethylene
PT	plasma treated
PTFE	polytetrafluoroethylene
P3HT	poly(3-hexylthiopene)
PVDF	polyvinylidene fluoride
PVDF-HFP	polyvinylidene fluoride–hexafluoropropylene
rHCP	random hexagonal close packed
RISC	RNA-induced silencing complex
RNA	ribonucleic acid
RW	random walk
SAW	self-avoiding walk
SCFT	self-consistent field theory
SEC	size-exclusion chromatography
SFC	semiflexible chains
SEM	scanning electron microscopy
Silica	${\mathrm{SiO}}_{2}$
siRNA	small interfering RNA
SLS	selective laser sintering
SMP	shape-memory polymer
SCNE	structurally constrained neutral evolution
SPAES	sulfonated polystyrene grafted poly(arylene ether sulfone)
SWCNT	single-walled carbon nanotube
TAPE	tris[4-(4-aminophenoxy)phenyl] ethane
TENG	triboelectric nanogenerator
TGA	thermogravimetric analysis
TN	three-network model
TNV	three-network viscoplastic model
TPMS	triply periodic minimal surfaces
VDA	vibronic-driven action
VMD	vacuum membrane distillation
XLPE	crosslinked polyethylene
